# Supplementary material for: Comprehensive Analysis of Rodent-Specific Probasin Gene Reveals Its Evolutionary Origin in Pseudoautosomal Region and Provides Novel Insights into Rodent Phylogeny
Source: Biology (Basel). 2025 Feb 27;14(3):239. doi: 10.3390/biology14030239 (PMC11940140; doi:10.3390/biology14030239)
Supplement: Supplementary file 1 [file biology-14-00239-s001.zip › Figure S1.pdf]

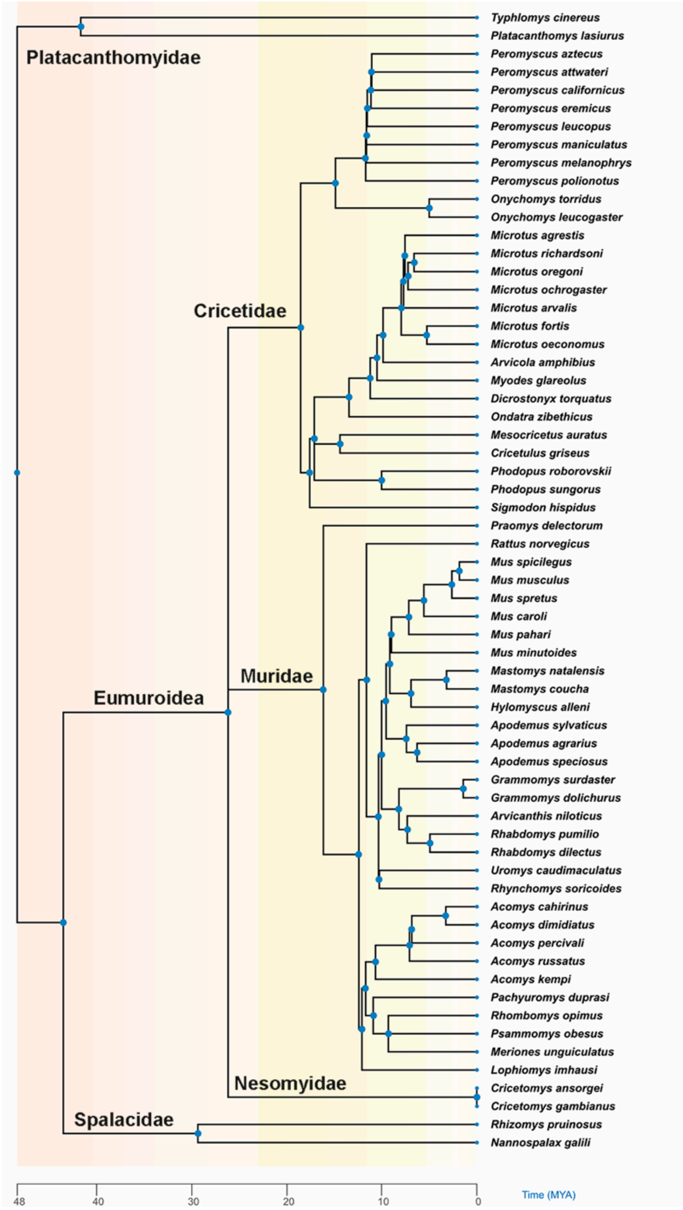

Figure S1. Evolutionary relationship of the different species depicted in Time Tree
A Time Tree generated by Mega X [1,2] has been calculated using the species names which have been subject to phylogenetic analysis. The following four species could not be identified by the program and, hence, not be included: *Meriones psammophilus*, *Peromyscus nudipes*, *Phyllotis vaccarum*, and *Rattus rattus*. Major branches within the superfamily Muroidea are added for orientation, i.e., Cricetidae, Muridae, Nesomyidae, and Spalacidae. Mya, million years ago

References

[1] Kumar, S.; Stecher, G.; Li, M.; Knyaz, C.; Tamura, K. MEGA X: Molecular Evolutionary Genetics Analysis across Computing Platforms. *Mol. Biol. Evol.* **2018**, *35*, 1547-1549.
[2] Stecher, G.; Tamura, K.; Kumar, S. Molecular Evolutionary Genetics Analysis (MEGA) for macOS. *Mol. Biol. Evol.* **2020**, *37*, 1237-1239.
