# Supplementary material for: Comprehensive Analysis of Rodent-Specific Probasin Gene Reveals Its Evolutionary Origin in Pseudoautosomal Region and Provides Novel Insights into Rodent Phylogeny
Source: Biology (Basel). 2025 Feb 27;14(3):239. doi: 10.3390/biology14030239 (PMC11940140; doi:10.3390/biology14030239)
Supplement: Supplementary file 1 [file biology-14-00239-s001.zip › Suppl Data Files/gPBSN/gPBSN_Acomys percivali.docx]

>CAJRSG010005723.1:c7341-1 Acomys percivali genome assembly, contig: Contig232813_ctg1, whole genome shotgun sequence

TGTGTGTGTGTGTGTGTGTGTGTGTGTGTGTGTGTGTGTGTTTCTCTGTTGTTCAAAGTACCTTTCCATAATTTTCTTTCTTCGACTTTATTAAATTTTGTCTCTTTCCTTCTTACACTATCTCTTTCCTACTTTCTCTTTACCTCTTCAAATAATGTGTTTCTTTGATATATTTTTTCCTCTTTGGTCTGTGTTGCATACAAGTGTAGAGTTCAAGCAAGTGGTGTGAAATAACATGAATCTCACTGTCAGGGACAAGAGGAAAAACTACATGATTCTAAAAACCTTCATACTTTCCATACAACAAATTTGGCAAAATTATGGTTCAGTGGCCAAATTCACACTGATTCCTCAGCTGAGTAACTGACAAAAATAAAATCTTATCAATATGTCAAGTTTAAATGTTGAGTCATCTATGGGGAATATTGAGAAGTAGAAGAAAAAAGAATGTAAGAAAATAACATTGAGCCACTCTCCATTACAATGTGGTTCATGAGTCCTACCCTTAAATATTGGTTTCTGGTGAAGGGAGAACAAATTTTGAGGTGTGTGGACCTTAGGAGATCCCACAACTGTGAGTATGTAACCTAGACAAATATGACTCTGTGGGTTATTTAAAAAAGAAAAGAGGGAGAAAGTAGAGGAATGAGAGAGCAGCTGGAAGGATATGTATGAAAATTTCAAAAACTTAATAAAAGTATTACATTCATTAGAGTAACCTTAGGCATGCCCATTGTGAACAAGTCATTGTGTAAATGAGGCATTGAACAACTAACATAAAGTAAAGATTCTTCCTCAGAAGGCTTCATATAGATTCTTCTCTTTTGTTTATTAGAAATCGTATATCATCCTTGTGCAATCCTGATGGGAGGAGCACAAGAAGGAGCTCCCAGATGCTTCTGTAAAATTCTCAGAGCTCTACAGAAAGTGCTTGAAGAGGTAGAAGACCATGTCTGCCAAAGAAAAGGGAAAGTTTGATGACATGGCAAAGGCAGACAAGACTCATTATGAAAACGAGATGAAGACCTACATCCCTCTCCATGCCACCCCACCCACACCCCCACTAAGGGGAGACCAAACAGAAGTTCAAGGCCCTCAATGCACCCAAGAGGCCTCTTTCAGCCTTCTTGTTCGGTTCTGAGTACTTCCCCCAAATCAAAGGAGAACACCCTGGCTTATCCATTGGTGGTGTTGCAAAGAATCTAGGAGAGATGTGGAACATCACTGCTGCAGATGACAAAAAAAAAAACCCGCTGTGGATGACAATCAGCCTCATGAGAAGAAGGCTGCCAAGCTGAAGAGGTACAAAAAGCATATAGCTGCCTAGAGAGCTAAAGGAAAGTGCAGTGCAGCGAAATGAAGTGGTTAAGGCTGAAAAGAGCAAGAAAATAAGGGAGAGGAAGATTAGAAGGATGATGAAGAGTTTGAGGAAGAGGAGGAAGAGAATGATGAGGAAAAAAGGATGATGATGAATGAGTTGGTTCTGGCACACTTCGTTTTCTTATCTAGAAGGCATTTATCCCCATGTACACAACTCACTCCTTTTAAAGAATGAGTGGAAATGTAAGGCTCTGCAAGATTTGTTTTTTTAACTTTACAGTGCCTTTTTTTCCTTTGTGAACAGTTAGCACAATACCGAATGTGTCTTTAGTTTGTCCTGTCCTGGTGGTGTTTTCAATGGTCACTAAGTTTCCTGGTACATTCCATGGGTTGTAAATTGCCATGGAAATTTAAAGCAAGTTCTTGTTGGTGCACAGTATAAATTAGTTATATATTGGTATGGTAACGGTTTTTGTTGTTGTTGTTGTTGTTGTGTTTTCTTTTCTTTTTGGTTTTGTATTTTCTTTTTTCTCTTTTTCCTTTTTTTCTTGAGTTATCTGTGATGCAGCTCATACAAAGATAATTCTCCTGTTATCTGAATACCACTCCCCAGTTGCAAAAAAAAAAAGTGGCTGTTTTGTAATCATTCTGAGTGCTTCTGAGTAAATACACGTTTTTATTTAAAAAAGAAAAAAGACCTCATATCTCCAACTTCACGTAAACTTTATTTAAAACCAAAACCATTTGTAATTAATACAACACAAAAGCCCCTCCATCCTTATTTTGAGGATATGAATATTGTCACCTGAGATAGATTGACCACTTACTTTCACTTCTTTGATATGGCATGTGCACGCCTTTACTCACAGGAACATAAACGTGTATGCACACACACACAGGAATTGTAAAAAGCAGACACATTTTCCTGAAGAATATGTTACTTATTTGTTGCTTGGTTGGTCAGTTGGTTGGTTACTTTTCTTGAGGCAGGGTCTCATTAAGTCACCCTGGCTCCCCTGGCACTCAGTATGTAGACCAGGCTAGCCCAGAACTCACAGAGGTTCCCCCTGCCTCTGTCTCCAGAGTGCTGGGATGAAAGGTGTGCATCACCATGCCTGGTTGAGGACATTTTTTTATTCAAAGCAAACGTCATTGTCCTGTGGGGAATTCTCACAAGTGTAGCTATAAAAGCAGAAAAATAGCATCATGAACTTCCTGTATCAGCGATGGTTCTGGAACAGAATGAGTAAGTGTATCCAGAAATTGCGTGTAGGATGAGTGAAATTGCTGTTTTATAATGTTTCAGATTCCTGCTTTACTTCTCAAATATATGCTGAATTTTCTTAATTGAGAATTCCATGCGTGCACATAATATGCTTCAATTAATCCCACCCTAGCCCCTTCAATACTTCAGTAGGTGGCATTGTTTGAGGATGCGAAGGAGAGCTGGCCTCCTTGGAGGAAGTATACCACTTTGGGGAATGGGATTTGAGATTTCAATGCCTTGCATAATTTCCAGTTTGCTCTGTGTTTCCTACTTATGGACCAAGATGTGAGCCCTTGCCTCTGGCTCCAGCTGCTGACATGTTGATGGTGATGATGATGGAAGCTCATGCCTCCATAACAGTGATCTTGGATAAGCATTTACTCCTGTAGGTTGCCATTTATACTTTCTTTTTGTAGTGCTCCAAAGACCACCAGAAATATCAAAATTAAAAGGAGGGAGACATGTTTACTTGAATAATTTTTGCACTTTAGAGTAGGCTTTGATGATTTCCAAATTGTTTTGTATTTCAGAACTACCTTCTTGAGATTTGAGACATCTCAGTGGGTAATGGTGACTGAGTACAATCTTTATAACCCACTTTGCAGAAGGAGAGAGCAGATAGACACCAATTATCTTCTGACCTCCACACACATTCCATGGTATGAACATATCCAGTCCTATATACAAAATAAATAATAAAGATTTAATACTTGCTACACAAGAAATCTAATAAAGGTGCTAAGTATAAATTCAGCTGAGAATCAAAGTAATTGTAAAACCAACTAGTTTCGTGATAATTTTATTGCCAAGTCCATGTGGATACAGAAGCCAGATCTGTCTCTCTAGCTGTTTTTAATATGCCACATGGTTCTATGCAATAAAAAATGATACTGTCTTTTAAAGAGAAAAAACAACTGTATGCATGCATTATTTTATATTAAAAGCTGTCAGGGAGGCACGAGTTCACATGTTTAATTTCAACTTTCAGGTTGTTGGATTCTCACAGGTGGAAGGGTAAAATTTACCAAAACTTTCATAAGCACATGCACATGGACACACACACACACACACACACACACACACACACACACAATTAAATAAATAATAAAATATAAAAGTAATTGAAAATGTAACCATATATATCATTATTACCCAGTAAGTGTTCTGGATGTTACCTGGGTTACATGGCTCCCAATTGATGGGCTGCTAAATTTGAGTGATGGTATGTAAAGAAACAACAATAACTTATATCTACAAAAGCAAAACCTTATCTTTGGAATCAAGACAGCATGTTCCACATTATCACAGTTGTTTAAACAAAGGCTAGAACCTCAAGTCACACAAACAAATATGAATTCTACTCAAGCCAGTTTACTGTCTTGTCCTAGGTCATGTAAACAATGTGGTTTGGGCCAAATCAGAGCAGTGTCCACAAGTGCATTCTAGCGTCCCCTCTATTCCTGATATTACATCCATGGTTCACGTACATGGGTGTTAAAAAGTTGATTGATTGAAAATGGTAACACTTGACATTCTTCCCCTAACATCTGATTGGAGGAAGAGAGACTAGGAATTGATAAATGAGAAGGCTCAATTGAAAGAAATAAACACAACGATTTTCCAATTGTGTAGGAATCAGTTGTGCCCAAAAAATAAACCCAAGTAGGGATTAGAAATTGTATTTTTATTTAACCACCAGCTATGCGAAAGTTTTAAGTTTAGACATTAGGTTTAAACAGCCTTCCTTCCAGTTAAAATAATATGATAACATCTGGTTTTAGTCTTCTGTTTTCTTAATACTAACATAAAGCCAATGAATAAAAATCTGCCTGAAACATGGGCCAGGCATTGAGCATCAGAATTGACAATAAAAGTTAATTGTCCATGCCTAGTAAAGTTCTCCAGGAACATATTTGTATAATAAATGACCTGATGTCAATGTCAGTGCTCAAGTGCCAACTGAGATGCAGGACACTGCTCAGGCAACCATCTTGAAAGGCAGCTATAAAAAGCAGGGAGAGACTCTGAGCATTGCCAATCAGTGATCTCCGGGCACCTGCAGGACGGACAGCCGCTCATGAACAATGAGGGTCATCATCCTCCTGATCACACTGGATGTACTTGGCGTCTCCAGTGTGATGATGGATCTGAGTCTCAAAAAGAAGGTAGCAGGCCTGCATGGGAGGGTGCTGGGTAGAGTAATTGTGCTGAACAGAAACAGACACAGAGAGAGGCTTGGGAGGAAGCTGTGCAGGTGGCTGTATGGGGCAGCCAGAGACAGAGAAGCAGATAAAGACAAAGGGAGGAGAAAGAATGGTCTAGAAGTTGGCATTCTCTATGTCTGGCAATGGAGAGTAGCAAAAGGGGTGGGAGTAGCCTAATGGATGGTTTTCTAAGAGAGAGAAACAGAGAGACAGAAATCAAGAGATACAGACCAGAGAGGTAGAAAGGAGGGAATGCTCTGAAACTTGGCATTCTCATGGGAGAATGGGGAAACAGAAGGATCCAGAGGGTCCTAGAAACCTACAAGAAGAACATTATGCGGGCAGATCTGGTCCCAGGGGTCCTGCTCAAACTATGGCACCAGCCAAGGACAATATGTGCAGTAAACATCAAACCCCTGCTCAGATCTAGTCAATGGACCGGACATAATACACAGTTGAGTGGAGAGTGGGGGCTGACTTTCACAAAAACTCTGGTGCCCCATATTTGACCACGTCCCCTCGAGGGGGAGGCCTGATGGCACTCAGAGGAAGGATAGCAGGCTACCAAGAAGAGACTTGATACCCTATGAGCATATGCAGGGGGACGAGGTCCCCCTCAGTAACAGTCATAGAGGAGGGGAGTAAGGGGAAAGCGGGAAGGAGGGAGGAATGGGAGGATACAAGGGATGGGATAACTATTGAGATGTAATATGAATGAATTAATTAAAAAAGGAAATTGGCATTCTCTACATCTTGGAGTGGAGGGAGACAGAGAGAGAGAGAGAGGATGGTCTTAAGGGTGGTTACTTCAGATAGAGACATAGCGAGAGGCCAAGAGACAAAGAAAAAGATACACACAGAGAATAATGCTTCTGAACGTAGGTTTTCTGTGTGTCTTTGTGTGTCAGAGAGAGATGGGAGAGAGGGAATAATGTTCTCAGAGTGTTCTATATGTCCTAGAGTGGAGAATGGCAGAGAAAGAGAGAGAGAGAGAGAGAGAGAGAGAGAGAGAGAGAGAGAGAGAGANNNNNNNNNNAGAGAGAGAGAGAGAGAGAGAGAGAGAGAGAGAGAGAGAGAGAGAGAATTGTCTAAGAGGTGGTTTTCTGAGAGGCAGAGACTAAGATTGAAAGACACAGAGGCAAACATAAAGAGAGACATGGAAAGAGAGAGTCAGAGGAAGAAATATCCTTCAGTCTGGTACTAAGACTGAGTCTAGCTTTGCCATTAAGTCTGCATACCTACTTCGTTTCCCATGTTTCCACTTCTGCCCCCACACCCTGTGAAGAAAAGGAAGCCATGATGATGAGTATGATGGAGTAATTTTAAGTAGATGGATCATACTAATGTTTCTGCTTGCAGTGGCTACTGTCTGTCCACCTTGGCTTGTTATCCAAAAGATTAAAAGTAAAGACCCGCACACATTTGCAGAAAAAGTAAAATGACTCCATTCAAGGTGAAGCTAGATTGCAGATTACAGACAGACACCCTGCCAGGATTGACAGCTGGCCACATGACTGACAGGACTCAAGGTCCCCAAGCTATTATTTGTGTCTCTTCTTGGAGTCCCAAGGATGGATTAGCTGGTGACTGAGGGGCCTGATTTATTTTGATTGACAGGTTAAATCGTTCCTTGGTCCAACTGCACATGTCCTTCTCATAGCGCGTCTGATTTTAATCATACATATGCCTGATTACACAGATTCATAAAGGTATCAAACAGAGTAAGGTCATCTGAAGAACATATTTTTGTCTCACTATGCATAACGCATACACCTCAAACCAGTCGGGCCATGTCAGTGTTTCTGTTCTTGCTACCTGCATACATTGAGAATATACCTGAGTGGAAACTTGTCTGGACTTGATCATTGCTATGGGTGTATGAAACCCATCATTATTCTTCAGAGACGGCTGTGTGGCTAGCCAGATGCAGTGGGGAGGGAGTTATGAAGGGATTCTTATGTGGGAACCCCTACAGCATTGAAAACAGAGTGTCCGAGTAACCTGATGTAGGTGGGGAAGGGGAGGAAGGGGTGTTATATAGGGAACTCTACACCATTCTTCAGAGAGGGGTGTGTGAGTCACCTATGCAGGTAGCTGTTGGACGGGTCTGAAATCATTATTAGGTCAATTGCTCAGAGTATGCATTAACATAAAGAAGGTAGCCTGCCTAATGCCTTATTACTAAATCAGGTTGTGAAGTGCCCCCACCCAAACAGAGTCCCTGGTTACTAGTCTTACCTGCCTTGTTTGCAAAGCCACTTTTTTCTTCCAGATTGAAGGGAATTGGAGAACTGTTTACTTAGCTTCCAGTACCATAGAGAAGATAAGTGAAGGCTCACCCTTGAGAACCTATTTCCGTCGCATTGAGTGTGTGAAGAAATGCAAGGAAATCTACCTCTATTTTTATATCAAGTAAGATATACAACAAAATAGACAGAACAATCTATGTGATGGGCTGTGCTCAGGGAAATGAGATGTTTTCTGTCAACACTCATGCCACTGAACACCCACAGCAGTAAATCAGGATTCCTACACCACACTGTTCTGTGT

>CAJRSG010010506.1:c45000-28000 Acomys percivali genome assembly, contig: Contig228462_ctg1, whole genome shotgun sequence

NNNNNNNNNNNNNNNNNNNNNNNNNNNNNNNNNNNNNNNNNNNNNNNNNNNNNNNNNNNNNNNNNNNNNNNNNNNNNNNNNNNNNNNNNNNNNNNNNNNNNNNNNNNNNNNNNNNNNNNNNNNNNNNNNNNNNNNNNNNNNNNNNNNNNNNNNNNNNNNNNNNNNNNNNNNNNNNNNNNNNNNNNNNNNNNNNNNNNNNNNNNNNNNNNNNNNNNNNNNNNNNNNNNNNNNNNNNNNNNNNNNNNNNNNNNNNNNNNNNNNNNNNNNNNNNNNNNNNNNNNNNNNNNNNNNNNNNNNNNNNNNNNNNNNNNNNNNNNNNNNNNNNNNNNNNNNNNNNNNNNNNNNNNNNNNNNNNNNNNNNNNNNNNNNNNNNNNNNNNNNNNNNNNNNNNNNNNNNNNNNNNNNNNNNNNNNNNNNNNNNNNNNNNNNNNNNNNNNNNNNNNNNNNNNNNNNNNNNNNNNNNNNNNNNNNNNNNNNNNNNNNNNNNNNNNNNNNNNNNNNNNNNNNNNNNNNNNNNNNNNNNNNNNNNNNNNNNNNNNNNNNNNNNNNNNNNNNNNNNNNNNNNNNNNNNNNNNNNNNNNNNNNNNNNNNNNNNNNNNNNNNNNNNNNNNNNNNNNNNNNNNNNNNNNNNNNNNNNNNNNNNNNNNNNNNNNNNNNNNNNNNNNNNNNNNNNNNNNNNNNNNNNNNNNNNNNNNNNNNNNNNNNNNNNNNNNNNNNNNNNNNNNNNNNNNNNNNNNNNNNNNNNNNNNNNNNNNNNNNNNNNNNNNNNNNNNNNNNNNNNNNNNNNNNNNNNNNNNNNNNNNNNNNNNNNNNNNNNNNNNNNNNNNNNNNNNNNNNNNNNNNNNNNNNNNNNNNNNNNNNNNNNNNNNNNNNNNNNNNNNNNNNNNNNNNNNNNNNNNNNNNNNNNNNNNNNNNNNNNNNNNNNNNNNNNNNNNNNNNNNNNNNNNNNNNNNNNNNNNNNNNNNNNNNNNNNNNNNNNNNNNNNNNNNNNNNNNNNNNNNNNNNNNNNNNNNNNNNNNNNNNNNNNNNNNNNNNNNNNNNNNNNNNNNNNNNNNNNNNNNNNNNNNNNNNNNNNNNNNNNNNNNNNNNNNNNNNNNNNNNNNNNNNNNNNNNNNNNNNNNNNNNNNNNNNNNNNNNNNNNNNNNNNNNNNNNNNNNNNNNNNNNNNNNNNNNNNNNNNNNNNNNNNNNNNNNNNNNNNNNNNNNNNNNNNNNNNNNNNNNNNNNNNNNNNNNNNNNNNNNNNNNNNNNNNNNNNNNNNNNNNNNNNNNNNNNNNNNNNNNNNNNNNNNNNNNNNNNNNNNNNNNNNNNNNNNNNNNNNNNNNNNNNNNNNNNNNNNNNNNNNNNNNNNNNNNNNNNNNNNNNNNNNNNNNNNNNNNNNNNNNNNNNNNNNNNNNNNNNNNNNNNNNNNNNNNNNNNNNNNNNNNNNNNNNNNNNNNNNNNNNNNNNNNNNNNNNNNNNNNNNNNNNNNNNNNNNNNNNNNNNNNNNNNNNNNNNNNNNNNNNNNNNNNNNNNNNNNNNNNNNNNNNNNNNNNNNNNNNNNNNNNNNNNNNNNNNNNNNNNNNNNNNNNNNNNNNNNNNNNNNNNNNNNNNNNNNNNNNNNNNNNNNNNNNNNNNNNNNNNNNNNNNNNNNNNNNNNNNNNNNNNNNNNNNNNNNNNNNNNNNNNNNNNNNNNNNNNNNNNNNNNNNNNNNNNNNNNNNNNNNNNNNNNNNNNNNNNNNNNNNNNNNNNNNNNNNNNNNNNNNNNNNNNNNNNNNNNNNNNNNNNNNNNNNNNNNNNNNNNNNNNNNNNNNNNNNNNNNNNNNNNNNNNNNNNNNNNNNNNNNNNNNNNNNNNNNNNNNNNNNNNNNNNNNNNNNNNNNNNNNNNNNNNNNNNNNNNNNNNNNNNNNNNNNNNNNNNNNNNNNNNNNNNNNNNNNNNNNNNNNNNNNNNNNNNNNNNNNNNNNNNNNNNNNNNNNNNNNNNNNNNNNNNNNNNNNNNNNNNNNNNNNNNNNNNNNNNNNNNNNNNNNNNNNNNNNNNNNNNNNNNNNNNNNNNNNNNNNNNNNNNNNNNNNNNNNNNNNNNNNNNNNNNNNNNNNNNNNNNNNNNNNNNNNNNNNNNNNNNNNNNNNNNNNNNNNNNNNNNNNNNNNNNNNNNNNNNNNNNNNNNNNNNNNNNNNNNNNNNNNNNNNNNNNNNNNNNNNNNNNNNNNNNNNNNNNNNNNNNNNNNNNNNNNNNNNNNNNNNNNNNNNNNNNNNNNNNNNNNNNNNNNNNNNNNNNNNNNNNNNNNNNNNNNNNNNNNNNNNNNNNNNNNNNNNNNNNNNNNNNNNNNNNNNNNNNNNNNNNNNNNNNNNNNNNNNNNNNNNNNNNNNNNNNNNNNNNNNNNNNNNNNNNNNNNNNNNNNNNNNNNNNNNNNNNNNNNNNNNNNNNNNNNNNNNNNNNNNNNNNNNNNNNNNNNNNNNNNNNNNNNNNNNNNNNNNNNNNNNNNNNNNNNNNNNNNNNNNNNNNNNNNNNNNNNNNNNNNNNNNNNNNNNNNNNNNNNNNNNNNNNNNNNNNNNNNNNNNNNNNNNNNNNNNNNNNNNNNNNNNNNNNNNNNNNNNNNNNNNNNNNNNNNNNNNNNNNNNNNNNNNNNNNNNNNNNNNNNNNNNNNNNNNNNNNNNNNNNNNNNNNNNNNNNNNNNNNNNNNNNNNNNNNNNNNNNNNNNNNNNNNNNNNNNNNNNNNNNNNNNNNNNNNNNNNNNNNNNNNNNNNNNNNNNNNNNNNNNNNNNNNNNNNNNNNNNNNNNNNNNNNNNNNNNNNNNNNNNNNNNNNNNNNNNNNNNNNNNNNNNNNNNNNNNNNNNNNNNNNNNNNNNNNNNNNNNNNNNNNNNNNNNNNNNNNNNNNNNNNNNNNNNNNNNNNNNNNNNNNNNNNNNNNNNNNNNNNNNNNNNNNNNNNNNNNNNNNNNNNNNNNNNNNNNNNNNNNNNNNNNNNNNNNNNNNNNNNNNNNNNNNNNNNNNNNNNNNNNNNNNNNNNNNNNNNNNNNNNNNNNNNNNNNNNNNNNNNNNNNNNNNNNNNNNNNNNNNNNNNNNNNNNNNNNNNNNNNNNNNNNNNNNNNNNNNNNNNNNNNNNNNNNNNNNNNNNNNNNNNNNNNNNNNNNNNNNNNNNNNNNNNNNNNNNNNNNNNNNNNNNNNNNNNNNNNNNNNNNNNNNNNNNNNNNNNNNNNNNNNNNNNNNNNNNNNNNNNNNNNNNNNNNNNNNNNNNNNNNNNNNNNNNNNNNNNNNNNNNNNNNNNNNNNNNNNNNNNNNNNNNNNNNNNNNNNNNNNNNNNNNNNNNNNNNNNNNNNNNNNNNNNNNNNNNNNNNNNNNNNN

ACACCCACAGCAGTAAATCAGGATTCCTACACCACACTGTTCTGTGTGGTCCAGAAAAGGACATACAATATCTGTACATTTAATTTAGAATGAGTAGATATATTTAAAGCCAAAGTAAAATAATAAATAGCAAGATATAGATGATATTTATATACAGATGATAGATAGATAGATAGATAGATAGATAGATAGATAGATAGACAGACAGACAGACAATACATATACACACCTTTAGATAGATAGGTAATAGATAATTAGGTAGACAGATGATACATATATACATAGATATATAGGTAGTTAGATGAAAGAGAGATAATAGATGGATTAGTAGATACATATATACATAAATACAAACATAAGGAGGTTGGTAGATATAGATAATAGATATAATGGATAGATGACAAATATACCTGACTATATGGAGTGATCAGAGAATACCATTAAAGAAACGGGATTGGTTGTAAAATGCATCTGGGAAACACAATGTTCAAAATCAGCTCCTGGTTGAAGTCAAATAGCACAAGACAATTTACCAAAGTAATATACATTAAAATCACTTAATTTTCTATTTATCACTCCTCAAAAACCCAGGTAGGAGAGAAGTTATATCTAAAAATTTGGGACAATTCCAGTGCTTTTAAACTATCTGGACATGAGTCAAATTATCCATTGAGGATATATTTCTATCTAGAATCAAAGCTTTTGTTAATTACATGCTGATACTTGAACTTTTTCAATGATTATTTATAGTTTTAGTTTTCTGAGACAGTCTCTGTACATCGTGATGGCTTTGAACTGAATACAATGTGAAGAATAAACTTTTTAATTTTTATTTATTATATATACTTATATAATAAATAACATAAGAAATATATCAGTTTTCTGGACAGTGTTTCTCTGTGTAGCCATGGCTGTCCTGGAACTCACTCTGTAGACCATGTTGGCATCAAAATCAGAGATCCACCCATCTCTGACTCCAGAGTTCTGGGATTAAAGGTGTGTGCTACTGCCACCACTTGGCTAATTATGCATTTTTTGAGATAGGTTCTCATATATTCCTGGCTGGCCTTGAATTCTTTATGTACACAACGCTGGCCTTGAAATCATAGAGATTAACTTGCCTCTACCTCACATGTATGGGGATCAAAAGTGTGCATCATCATATCAAGCCTATATTTTATGTTTTTAAATTTACTTTCATTTTTCATTATTTTTTTCAGAATGACAGGGAACCTAGCCTCCTGCATCAGTCTGCTAAGTATTGGGATGAAAAGAGATGACCCCAAGCTTGATTTGATATTTAGACTTAACATGCCTAAAATTATTTTTAAAGTAATAGTTCAAAATCAAGTAGGTATACTTCAACACTAATATGCTGAAGGGGATATTAAAATTTTTAATTTATAAATACTCTTTTTGGAAATAAAACATAATTACACCATTCCTCCTTTCCTTTCTCCCTTCAACCTTTCCGATAGATTTTCTTTTAGTTTCTTTCAAATTCTAGGGTCCCTTTTTTAAATTATATATATATATATATATATATATATATATATATATATATATATATGTTCATCTATATACATATATATAAACTATACATTATATTCCATATAAGTTATTTATATAAATATGATTTCATGTCATCCACTTGGTATTTGAAAAACCAATGAGGAAGTAGGGCTTTTCCTCAGGGAAGACTATAAAATATCCTAAAATTCATATGGAACCAGAAAAGACCCTAAGGTAACTAAAACAATCCTGAACAAAAAGAACAGTGCTGGGGAGATCACCATTCCATACATGAGTGATATATTTTAAGCACAGAGAATATACCCTAAGGCATGAGATACATCCCTGGCCTAAACTGTTATTCCAAATTTTTTAATTTTTATTTTATTTTTAGTTGTGAGCCTAACCTTTAATGGCAGAGACATCTCTCCAGCTCAGTTATTCCCATGTTCTTAAAGCCATAAAGACAAATATAAACTGTAGCTGTCACTATTAGTCACATGCTCCTCTGGTAAGTGAATACAGATATGAATACGTGACCATGCCTGCAGCTCTAAGAATCTCCTGATCTGTATGTACATTCCAATTTAAATCTTCCCAGAAAACAATTGTAGGCTGAATTTGAAGCCATTTAAGTGCACAAAGCAATTGCTGTTGTAGAACTTTTAAGATTTGTGACATTTATGAAAGGCAAAAAAACCATTAATACAGTGTACATATGGAAGTTAGACTAGAATGTACAGTAGACATGGCAATATGCACTGCACCAGGTGACCTTGCACCCTTTTAACCCAATGTCCACAGACATTTCTTATATCAGATTGGGATAGAATCTTTGTGCACCCTTTTAACCCAATGTCCACAGACATTTCTTATATCAGATTGGGATAGAATCTTTGTTCAATACTTTTTTTTCTTTTATCAATAGTTGTCATTGGGGGCTGGGGATGCAGCTGTGCTGGGAGAATGATTACCCAGCATTCACCACAGAGCCCAGGGCTACATCATCAGCTTGAAGGTGTGGTCCTACATTCCCATCATCCCAGCACTTGCTCCATAGAGGCGGGAAGATCAAGGGGTTCAAGGCCATTCATTGGCCAGAAGTCAGGCATAGGCAACAGTTGTCACACACAGGAACTGACTCTGTCCTATCAAAGACACCTAAAAAGCCATAACAGGGTGATTGATGACATATTAGGATGCTATTGATTATATCTAGGGATGATGTCTTATAATAACATGTGCTTATGACACATCTATATATGAAAATGTGGTTTGCATTAAAATGTCTTTCATTCGTATGGAAACAGGGCCCTGAAGTAAATCATAAGAAGACAACATCTTTGCATCTGTTTTTCAGGAGAAGGGCCAAGTGCCAACAGTATAAAATCATAGGAAAAAGAAAACAAGAATTTTACCAGGCAAAATGTGAGTAGAAGATGCTGGGGTGAATGCATGACCTTATTTTTTTTTTTCAAATTCATGGTCTTAGTTGAATTTTGCATATCTGGATATCTACATGCCAGTGCAATCATTTCTACAAATTACAGACGCAATCTTGTAGAAAGACCACCACTTTTCCTAGTCATTATTGGATCTAATAAGAGATGTGATTTGTTTGTTTGTATTTTGACTTTTTAAAACTTTGTTATTTATTTAGTGTGTGTTTTTACCAAACACACTTCTTCCTTCCTGTAACTTTAGGCCTAAACTAAATCACAGGCAAAGTCTGAGATTGGTTAAAGGAATCAAAAAGTGATTTGAACATTTAAAAAAAATAGCTTGATGCAAAATAGGAACTAGCATTTTGTCATAGATTAAACTGACTCCCAAAAATTTTCAAACATGAGTCTTGTATGTGGAATGAATAATGATCTAAATTTTACAATTTTATCAGGGAGAAAAATTGTTTACAATATGTAATTATAATCTTCATGAATAGTCCTATCTTTTTGGTTCTGACAGAAATGTGACTTAAAACCCACCATGATCCATTTATAAATAACAGAGACATCAGTTTAATTTGTCATGAAACAAGTAGCAAGGACACTTGCATTATTTCCTATTCCACTATAAGGGTCTATTTAACCCAAGACTGATAGACCAAACATGTCACCAACTATACCTTGGTTTATCAATAAGTTTATTGGATTTAAATACAGGAGCAGACTCAACTCATAAACAGATATCTCACCAAAGAATCCCAGCCCCTCCTGTGTGTTATATAGTTTAGTCTGAGGCCAACCAGGTCTATACAGCAAGTTTCAGGCCAGCCAGGGCTACATAGTGAGACCCATCTTAGAAGGAATATGATGTACCACTGACATGAGAAAAGAACAGTCATCACATCGTTGGGGAGGGTCTGGTGCCTGCCCCTTACCCTCTATAAGGAGAAATGACAGTGAGCCAGTGAGTGTATTGGAGTCATTTACAAGAGCATGTGTGACACAAATATGACTGCATTCATAAAGAGACCACCTCAACTTGGGTGACACACCTGAAAGCTGTATCCCTGGAGCTTTGTGTAAGGCTTACAGACAGATGATCTAGTGAGAGAGTCTCTTTTTCAGGCATTGTTTACTGTTTCCCATTACTTTGTGGAGTGGCCTACTAAAAGTTGTTATTCTCAAAAATTGCCCTAGCAGGGTGTGGTGGTGCACACCTCCCAGCACTCGGGAAGCAGAGGCTGGTGGATCTCTGAGTTCAAGGCCAGCCTGGTCTACAAAGCGAGTCCAGAACAGCCAGAGCTGTTACACAGAGAAACCCTGTCTCAAAAAAACAAAAAAATAATGTCCTGAGATTTGTGGGTTATTTACTTCTTGAATCTCATGCACTTCCAAGAAAGTTCCAGTTTGTAGAAAATTGCTATGTGGTACCTTCTCTTATTTTTCTGTCACATGTCTGAGGAAGAATTAGTAAAGTTAGATAATTGCACCACGAAATGTAAACCAGATCCATACAAATACCTCCATATATGTGTGTATGCTAATATATTATCATACAGCATTCTTTAATGTATGTGCATATATGTGTGTGTGTGTCTAGATTATTCCACCATTAAATGTAATAAGATGCTTTAAAAAACTTATATATGTACCTATATACATATACATATCATACAGCTTTCCTCAATATGTGTATAGGTATGTGTAAACAATTGTATCAATAAATTTACTAGAATCCATTAAAATATATATGTATATACATATATATGTATGGGTGTGTGCTGTTCTCAGCAAGTAAGAGAATTACAATGTTCCATCTTCTATGTACCTATACATCCAGGTATCAGGACCATTACTATCCCAAATCAATATATGTTCTACATACTTCTAGGTAACTGTGGTATACTAGATATGTTTTATATCTTACAACAGGAATATAAACTTGCTTTCTTAATTCCTTGTCTGTCTTGACAGGTTTCAACACATGGAGCAGAATATTATGGAGCAGAGACTGTGTAATCTATGTACATATCCATAAGGGTATAGTTAGAATGAGCCTGAAAAATCATTTTGTTGACTTTATACATATTTCTTATCTATCTCAGATGAAGGGAAAACGACATTCATGGTAAAGATGGTGAATGAGAAGATATTGCTGTTTCATTATTTTAACAAGGACAAGAGACACAAAGTCACACGTGTGGCTGGAATTTTGGGTGAGCACCACAAGTGGAACTTGTCATCTGAGTGTGTAGTTCAAGGACATGGGTGCATGTGCATCACTGTGCATGAAGTTCACAGTGTGCTCATGCATCTGACTCTGTTCTTCATGTTGATAAACTGCTGAAATAGTATTGAGAATCAACGTGGCACAACCCAGAATCCCCTGGGAAGAGTCTCAGTGGGGAATTTCCCACAGCAGACTGGCCTGTGGGCATGTCTTAATTGATGATTGACATAAAAAGGCTCCACCCACTGTGAGTGGAACCATTCCCTAGAAAGAAGAAGATGTGCAGTAAACTTCAACTGTCAACAGACACAAGTCAGCATCCTCTGGGAAGGGTCTTAGGAATTGATTTCTCTCATCAGACTGGCCTGTGGGTGTGTCTGGGGGAGTATATTAAATGATGATTGACATGGGGGTTTCTGCCCACTATTATCAGCACCATTCCCTGGCCAGATCATCCTGGACTGTATCAGACTGGAGAAAGCTAGATAAAGAATGCACATTCATACATTCTTTGCTCTCTGTTCCTAACTATGCATGTGGTATGTCATGTCCCCCAAACTTCTCTCAGTTGAAGTTCCCCAAGTCAGGGAGCTATAACTGGGAAATTACTACAGAATTAAACCCTTTTCTGCAATAAATTAGTTTTGTCAAAGTGTTTAGTCAGAATATTGAGCAAAGAAGTTGAGATATTGAAAGCTTGTTTGTTAAAAATAAGTACCTGATTATTCTTTTGGGACTTGGGAGATGTTAGTAAAACGTCAAGATATCTTTTTCCAGCATTTCTGAAGTATACATCAAGCAATTATCATGTGCCTCATGCCAATGTAGTCCTGGATTTCCATCTCCTAACAAAGCAAGAATTGACATATTACTTGACCACAGTAAATAGGTTTGTTGATTGAATTCATGTCAATAGCAAATCCTGGATTTCAGTCCTGTTGTTTTGAGCAAACTTTACAACTCCTTCCTTGTAAATATGAATTATCATGTAGCCTGAGGTCTGACTATACTGGACCTGTCCAGAAAACTCAATTTTCCATACATACAAACTCTCTTGAATTGACAAATTCTACAACTCTGTCATCAAAGGCAGAATTAAGCATTTATTTCTGAAATTAATAGGAATAATTTGTATTTCATGGGTGTATGTTCCTAATTAGCAGAGTTTTGTGTGTACTAGTGTTTTACCTGCAAGTATGTGCACCTTGTGTGTGTATGGTGCATACAATTGTCACAATATGGCATTGGATCCCCTGGAACTGGACTTAGAGATGGTTTAGCACCACCATGTGGGTGCTTGGAGCTGAATACGGGTCCTTAAGCATTCTTAACCAGTGAGTCAAATGTCATTCCTCTCATCTTTATGAACTTGAATCAGTTTGTTCTGTCATCTGAGAAGCATCCAGAACCCTTAGAAACAGATTCTAGAACATACACAGAAACACCTTATGACATACATTTATAATAGTAAAAACATTAAGTATCAATCATCATTTTCTGGAATTGATTTGGAGGCTGTGTAGGAATTCTATATATTCAGATCCACAAAAAATTGTCAGTGTTACAAGAATAGGAGGCAAGGTATGTTAAATACTGGAATCCACTACTACTTTTATTTCTCATGAGAAAGATGTAGTTTTTGACTGTCAATATACTGGAAGAAACAAGAGTGATGCTTTGTGTTAACAGTTTTTAGGAGACTAGGCAATGGCAAATATTTATTAATGTGCTGGGTGCTAATGTTGTGACTTCAACTTCTTTATAAATCCTGAAATGCAGCAAAATCCAAACAACTGACTAAGGATGAGATGACGCAGTACATGGACTTCGTGGAGGAAATTGGCATTGAAGATGAGAACGTACAACGTGTCATGGACACAGGTAAAAAAGCGAACTGTGTGTGCAACTCTTTACTTTGCATATTTATAATAAATGTTATATTTTAAATATCTGAGGCATTTGGAATGTGGTTCAGTGTGCAAAGTGCAAAGTGCACACAAACATGAAGACCCAAGTTTGGATCCCAGCACCCATTTAGAAAGCCAAACACCTCCAGAGCTAGGAAGGAATAAGGACAGGAGTAACCCTAGGGCTTTCTGGCAACAGATTTATCTGAAAGATCTCTTCTCAAAAATAGGAATGTGTATAATGAATAAGGAAGATGTTAAAAAGTCAACCTCTTGCTTAAAAGCAGCATGGGTGCACACATATCACTTGCATGAGTATGGACACACCAATGAACCATCAGACAGATCCATACACATAAAATATGCCTGGAATAAAACTCACCTCCTAAGATCCTCCGTTTGATATTCTGTTTAAAACCTAGCCTTTTATCTGCATTTTTATGAAAGTTGTTCATATGTAGTAATGATACTATTTTGTTAAAATAATATATTACTTAATATTATATCAAAATAATCCATTTTAAATAATAGTATTTTCCATGTTCCCTCTTTTCTAGATACCTGTCCAGACAAGATCAAGGCTAAGTGAGTTAAAGCTTGTTTATTTTTACCTTGATATTTAATTTTAATTTATCTATGCTTTTTAAATAATATTTATTTTGTCTTTAACCTTTTCATATATGGATATATGGATATTTTGATCTTACCCACTCACCCACATTTGTCATTCCCCCAGCTATCCCAGAGACCCACCAACACAACTCCCTCTATACTTCAAGACTTTTAAAATTATTATTATTATTAACTCAGAATACAATTGGTGCTGGTTGTCAATGAGGATGTGGCCATTTCTAGAGTCAAAGACAATGTAATGTACCAGGGGCCACACACTTATCAAACCCTGACCGCTGGGAGGTAGTTGGTACTCATATTTTATCTAGTACTGGGGAGACAAAGGCAAGGAGATCTCTGGGTTTGAGTCCAGCCTGGTCTACAGAGGAATTCCAGGACAACCAGAGCTACAAAGAGAAAGCTTGTCTCAAAAAACCAAAAAGAAAAAGAAAAAAAGAAAGAAAAGAGAAGAAATAGGAAAGCAAAAAGAATCCTCCTTTCCCTAAATCCATCAACTGTTGATAGTTCTTTGGTTAGGGGTGGGTGTTCCTGTCTCCCTCTCCGTTCCATTTTAGAATGTTGAATAGCTTGTTCTGGCCAGGGCCTTGTGCAAATGAACACAGCTGCTGTACGTTCATGGTTGTAACAGCTGAGTCATGTTCAGAAAACAAAATTTCATTGATCTCCTCCCCACCCAGATGACCATCTATGGCTACGCATCCACAGTCACTTAGCCTCAGCACACTGACTAGCTGTGAATTTCTGCATGAACCGCTCCCCACTGTGAAAGTTGACTTGAGGCCAGCTCTGGAGGCATACACCTTTAATCCCAACACTTGGAAGGCATAGGTATCTGAATCTCTGTGAGTTTGAGGTTAGTCTGGTCTATAGTGTGAGCTCTAGAACAGCCAGGATACATAGTCTCTATCTTTAAAAAAAAAAAAAGATAAAATGTGACTTGACTTGACCAAAGTTGAGAGCAAGATAAATCTATTGAGTAGTTTTTTAAGATGGTAGCTTGGAAACATGACAGTTTATCACCACTAGTCTCCCACATAGGCTCCGTGATCTCCATTGTCATGGGCTTTTGACTAGAATTATGGTAGGAACTCACTCCTACCCCTGTTCTTCCCTAGAAGTGAGTTCCCTTCCATGACATCAAATTTAATCGAAAAGTGGTTGGTTTCTCCAGGACAGCCTTCAATGCACCAGTGGGCACAAGGCTGTTTTTGTAGTATGCAGGGGCAGCATGGTGACATCATTAATTTCTTATCCCACATACACAGCCTATGCAACACTTGCAGGCACTGTAGAAGAGTTTCCTTGTCCATTTCAGATTGATTTCTTGGTGCCCTGTGGCCACAGCATGTGGTATCTTCAGCAATGGTGTCCTACCATTTAGCTATGGTAGACAACCAAGAGATATGGCAATAGCCATGTTGTTTTGATGATTCCAGGTCTTCCCTGGTGGGGCAGTAGCCTATGCCTGGAAGTTAAATTTTCATTGAATAACCCATGTCTTCTGAAAACAGCATTTTATCATTGCATGGATCCTCTGATGAAGCTCTTTTTAAATACTATGTTTTAAATTAGCTTACAAACTAGTGGGTTTCTGTAAGACTTCTTCATACACCTTTAGTTTTGGTTGAACCACCCTACCCATGTTCTTTCCTATACCCCAACCACATCCACACCTGCTCCTTCTTTAATCCACACTATTTTCCTTCTAATTGTCTCTGTCACCTGTGTTCAATGATATCACAGGTCTGATTATATTTTGTACTATACTCTCATTGTCATAAGTTTTCATACAAAATTTTAATAACACATTATTTTGGTCAAACCTTCCACCATGCTCTGATTTCTCCATTCCATGACTGAAACTTCCTGCCCTCAGTATTCTTCTTTATACTTTATTTTAATAGCATTTCCCTATCTCCCCTCCCTTGATGGACCCACCATGAATGATCTGTTTTTAATTACCTGGCTTCCATACATAGTCCAAATTAAACATATAAAATAGAAGATTCAAATCTAAGATCCACATTTGAGATAGAATTGCAGAGTTTGTCTTTCTGAGCCTGAGTGACCACATTGAGTATATTTTCCAGTTCCTTCTATTTGCTTGCAATTTTCATATTTCAGTTTTTTCTCTGTGGCTGATTAGTATTTTATCTTATACATTACATTTTCTGTGTCCATTCATTCATTAGTTGATGAACAGCTAGGTCAATTTCATTTTCTAGCTATTGTAAACTTAACTTCAATGAGAATGAGCATTCAAGGGTAACTGAAATAATATATAAAGCCTTTGGGTACATATCTAGAAAAGATATGTAGCAGGATCACATGGGAATTCTGTTTTTAATTTTTGGATTTGTATATGTTCATACTTTTTGTTTTTGTTTATTTTGTGAAAATTTAAAGTTTGCATATTGTAGACACTAATCCTCCATGAAATGTATAGCTGGCAAAGATCTTCTCCATTCCCTAAGATGCCTCCACATTTAATTGACAGCTTCCTTTACTGTAGTTTTTAATTCTGTGATATCTAAACAGTGTTAGTCTTACTTCCTTGCTACAAGAATCCTATTCAGAGAGCCCACACATGTGCTTATGGTAAACACACACTCTCTGCTTTCTTCTCTATCATCTTCAGACTATCAAGTCTTTCTTCAAGGTTCTTGATCCATTTGGAGTTGAGTTTTTGCACAGTGCATCTTGAGGTCCACTTTTCCCAGATTGTTTATCCTTCAGCGATTCTGTCTGTTTGTTGAACTATATATATTTAGGTTAGGTCATTTTCCTTATTTCGGTGGAGACATTGACAATATCCAGAAGAGAACTAATTCTTGCTAGTATCGACATGTCATTCTCCTCTCTGGGGCTGGTATTGAGGGCTGCAAGCTCCACTCCAGTCTGCTTCTGAGAGTCAGCTACTGTATTGGACCTTGAGACTCCTGCTGTAGGGTACATAGGGCTTGGTAAACCCCAATGTGAAAGCTGAAGCAAAAAAATACATATATAGATATAGATATATATTATAATTTTAGTTTAGATTCATAGAAACTATAGCCTCAAATAAGCACATAAGTCCATAAGTTTAAAAAAATCCCAATTTAGGTCTTGCTATGAGATCATTTATAATATTAAACTTTTCCATATATGGAAAACTTCCACACAAAATTCATGTGTAATTCCAAATATACAAAATCCTGTAAAATGTTTTTGCTTGATACATCTTGTCATAGTTTGCCTCTGTAATGGCTTGTATTTCTTTTATTTTCCACTCTCATGAAACATCATATATTATTATCTTAATTATATTAAATAATTCTGTTCCAATCTTACAGATGACAACATCTGGAATTTTCCAGTGTATTCTTCCTGAAACATCAATATGAAGATGAAGCAATTTCTCTCAGATAGCATCTTCCTATTTGCTGCAAATTACAGTTCCTGTATCCCTACTTTCTCTTCCACTCGTACTTTCCTGTGTTCTAATCAGTGTTAGTGCATCTTTGAATATTTAAATAAATTTATTTCACTTGCATACGTGTCTCTGAAGAAAGGAAACTATACTGGACCACTGAAGCCAGACAAGGCGTCTCAGCCAGAGGAAAGTGATCCAAAAGGAGGCAACTGTGTCCATGCTGGAGACAGCTCTGGTCCAATTGGTAGGTGCCCACATGAAGACCAAGCTTCAGTTCCAGTCCATGCACTCTCTGTGATTGGTGGTTCAGTCTCTATGAACCCCCGTGGGCCTAGGTTAGTTGACTCTGTTGGTCTTCTTGTGGGGTTCTTGTCACCTCAAGGTCCCTCTATCTCTTCCTCCAACTCTTCCACAGGATTCCCTAAGCTCCACATAATGTTTGGCCATAAATCTCAGCAAGTGTTTCTATCTGTTGTTGGGAGGAGCATCTCAAAGGACAGCTATGTTAGGCTCCTATCTGCAAGCATAGTGTCAGGGGTTGACTGTCTTCCATGGAGAGAGTCTCAAGTTGGACTGGTCATTCTCTCAATCTCTGCTCCATCTTTATCACTGCACATATTCTAGGTAGGGTAAATTTTGAGTCCAAGGTTTTGTGGATGGTTTGGTGTTCCCTCACTTCACTGGAAATCCTGCCTGAATAATGGAGGTTGCCACTTCAGTCTGCATATCCCTTGCTGCTAGGAGTTTCTGCTAGAGTCACCCCCATACTCTCCCAAAAGCCTACCCCAACACAGGTCTCCAACTTGTCTCAGAGATGCCCCACATATTGAGGGTAAAGTACTATATTGGTTGCTTAAAGTGGATATTAAAAACTTTTTGTGCCACTAACTTTATGACAACTTATCACAAGCCATAGTTACTTGGGAAGGGAGACACTCAATTAAGAAAATCTCTCCACCAGTTTCATCTGTGGGAAAGCCTGTGGTGTGTTTCTTGGATGATAGTTGATGGGGGTGGTCCAGCTCACTGTGGAAAACATTGCTGAGTAAACCATGAGAAGTGAGAAGTGAGTCAGTAATTGGCTTACACTTCCAGACTGCAGTCCATCACTGAGGGAAGTGGGTGCTGAGCTACCTATCAGCCATCTATCATCTATGCATCTATCTATTATCTATGTACCTATAAATCTACTATCTGCCTATGTGTGCATTCATGTATATATGTATCATCTACATACCCAAAAATTATAGAAGAAATCATTAATTTGACCATCAGTTTCTGATTCCAGGTTCCTACCTTGACTTCCTCAGGTGATGGACTACAAACTCTAAAATTAAAAGAACCCTTTTCTCCCTACAGTGTTCACAACAGCAATAGAAACCCTAAGACTCACAGTAGTGTTGGACAGCTTAGCTCTCAAATTATTTCTTAAATAAAATTGAAATCAAATGCCCAACTCTATGTAAAACATAATCTAGTACAGAACACCATAATAATTTAATTTCCATTTTCCTACTGAATAAACGAAGCTCATACCACTTCTCTTCTGGTTTCCTCATTTCTAGAAATTTTTCAAAAATATGTCTTTCTAAAATTTTTATTGGCATCCTTCATTACTGTATTTATGCATGTGAAACTATCAAATACCTAAAGTAAGCATATATGTGTGCTAGTTCTCAAATAAAATACATTACCAATGAATCAGTGACAGTCCCTGTTCCTATGAAATTACATAGATAGCACGTTGAAGAGTTTATAAAATAAAATTCTGCATGCAGAAAATAGTCTTCTTCTTTCTGTGTGAAATTCAAAGAAAAGGAAATGGGTTCTGATGAAAACAGTGTCAAAATGTGTTGTAAATGCGTTTTGAAAATGATCCAGTAGCAGAAAACCTTTGGGGGTCAGCTGATAGGTTCCCAATAGACTATCACCATCTAAACATGTAGAAAAAAGTTGGAAAATAGTACTGTATTTTGCCAAATCATTTGAGAATACCATGCATGAGTAAATTATATTAATATCATTAACACCCCTCCCTCTTTCCCTTTAACTCCCCTTGTGTTCCTCCACCACTCCTCAAATTAACAGTTTCTTCTATCATTTTTGTTTTTGGTGGGTAGATTGATGATACATACATGAACGCACACATAGCCAGACAGTAGATATATAGATGCATAGATGATAGATGGCTGATAGGTAGCACAGCACTCACTTCCCTCAGTGATGGACTGTGGTCTAGAAGTGTAAGCAGAAATAAATCTTTTCCTCCTCAAGTTGTTTTGGTCATGGTGTTTCTTCGCAGAAATAGTAACCCTAACTAAGACAGATCTTATTCTGGAAATATCTGGTTCTCCCTCTGTTACTATTGGGTGTTTCCATAGGCTTATAGATTTGAATGCTTGGTCACCAAGAGAGTGGCACTATTTGAAAGGATTAAAAGATGTGGCCTTGTTGGAGTATGTGTGGCCTTGTTGGAATAAACATGTCACTGAGGATGGGCTTTGAAGTTTCAAGAGCCTAAGGAAGACCCAGTGGTTTCT
