# Supplementary material for: Comprehensive Analysis of Rodent-Specific Probasin Gene Reveals Its Evolutionary Origin in Pseudoautosomal Region and Provides Novel Insights into Rodent Phylogeny
Source: Biology (Basel). 2025 Feb 27;14(3):239. doi: 10.3390/biology14030239 (PMC11940140; doi:10.3390/biology14030239)
Supplement: Supplementary file 1 [file biology-14-00239-s001.zip › Suppl Data Files/gPBSN/gPBSN_Apodemus agrarius.docx]

>OZ030010.1:c39524000-39507000 Apodemus agrarius genome assembly, chromosome: X

AGGTTGGGAGTTTCCAAAACCAAAACTTGCAGTGTTGATGAAAGGGAAATGACACAGAGGAGCATACTAACTCATGAATATCCATTGTCTGAATTTTTCTTCTCTCTTTTCTGTTGTGACATTTACTACTTAGTAACACAGTGACCTAAACAACCAGTGACATGGTAGGCTTGAGTCACCTCATTCAGGCTCATGTCCATCAAAGGTCTACATCACTCCCCTTGCAGATTGAGAGAAGCTGTGATATTTCCTACTGCCAGACAGCTGGTTGTTAAGTGAATATTTTAAGTCCTAAAATTTGTGTTAAATTTTATAACATTATTCATCACTTTGCATTGTTTACTTCTTTTTTAAAAGTTGAGAATTTCATGAATGCATACATGTTTTGATCAATTCCACCCTATCCCCTTCTGGATCAAGATGTAAGCTCCCAGATTCTGGATTGAGCCATTGCCATGATGAAGATGAAAATCTCTGAAACAGTGATCTCGAAGAAAAACTTCCTTCTGTAGGTTACATTTTCTACTTTAACAAATTTACTTTTTGTAGTGCTCTAATGAGTAACAGAGATGTCAATTAAAATTGGGGATGTAAGAGACATTGACTTGAATATTTTTTGCACTATAGTGTAGGTTTTAATTATTTCTACATCTTAGGGGGTAATGATGACTGAGTTTTATGACCCACTTAGTAGGATGAGGGAGAACCAGTACTCTTCTGACCTTCATACACATGCCAGGGTATGGACATACCCAGTCCCATGTACAAAATAAATGATAAAGTTTTAACATGTATTATGTACAAGCAAATTTATAAGGACTCTTATTGCATGTATATCTGAGAACCAAAGTAATTGTAAAATCAAGTATTTCCTGATATTTCTCTTTCATGTGCACGTGGATACAGAAGTCAAAACTGTCTTTCTGGATGTTTTAATATTGCACATGATTCTATGCAAAAAATACTGATACTGTCTTTTAAAAAGAAAAACTGTATATGCATACATTATTTCATATTAAACACTATTAAGAAGACATGAGTCCAAACATTCTATTTCAAATTTCAGATTAATGAATTCTGACATGTGCAAGAGTTAAATGCCCCAAATGTTTCATCAGCATAGGTTCCCAAAAACTGGGGAAACCCAGGGCTAATTAGATGGCTCTTCACATAGAGGTGGTTGCTGCCAAGCCTGGGAGCCTGAGACCAGACACCCAGATATATACAAAATAAGTAAGATAATGAAGAAATTTAAAATTTAACCATATTTATTATGATTATCCATTAAGCTTTCTGGATGGTGCTTGGGTTATATGTCATAATTGTGATTGACAGACTATTAAATTTGAGTGATATTTGTAAAGAAATAGTAATAAATTATATCTATAGAAGCAAGATTTTATCTTTGGAATCAAGACTGTATGCTCCATGTTATCACAGTTGTTTAAACAGACAAGGGCTAGATCCTCCAGTTCCACAAACAAATGTGAATTCTACTCAGGCCAGTTTACTGTCGTGTCTTTGGTCATGTGAACAATGTGGTTTGTGCCAAGAATAGAGCAGTGTTCACAAGTGCATTTAGCCTCCCCAGGATCGCTGATGAATCCATGATTCAGGTTCAAGGGTGTTGAAAACTTGATTGAAAATGGCCAGACTTGATATTCTCCCACCAACATCTATCTGATTGGAAATGGATAATAGACATCAGGTTTAAACATCTACCATTCCAGTTAAGATAATATGATAGTATCTTGTTCTTAGTCTTCCCTTTTCTTAATAGGAACATTAAGCCAATGAATAAAAATATACCCGAAACATGGGACAGGCATTGGACATTGGAATTGACAATAAAAGTCAATTTTCCATCCCTAGTAAAGTTCTCCAGGAACCTATTTGTATACTAAATGACACAATGTCAATGACAGTGCACAACTGCCAACTGGGATGCAGGACACTGCTCATGCCAACTGTCCTAAAAGGCAGTTATAAAAAGCAGGGAGCTACTCTGCACCTTGTCAGTGAGGTCCAGATACCTACAGAGCAGATACAGTTGCTCACACATGATGAGGGTCATCATCCTTCTGCTCACACTGCACGTGCTAGGCGTCTCCAGTGTGATGATGAATAAGAGTCTCAAAAAGAAGGTAGCAGGCCTGGGTGGAAGGGGGTTGTGTGTGGTGGGCATGCTGGGAAGAGACAGAAACAGAGAGATAGAGGCTTGGGAGGGGGCTTTGTGCAGGTGGGGGGGAGCAGCAAGAGACATAGAGGCAGAGACAGAAAGACCATAAAAGGGGAAAAGAATGTTCTGGAAGTTGGTATTTGCTAAGTCTGGTAATGAAGAGTGACAAATATGTTGGGGGCAGTAGTTGAGTCTCATAAGTTCTTTTCTAAAAAAAAAAAAAACCAGAGGGACAGATACCAAGAGGCAAACAAAGAGAAATGAGAGAGTTCCTGGAGAATTTTACTAGGGATGAACAACTACCTTCTAGAAGTTGCTATTCTCTACATCAGGATTGGAAAGAGATAGAGAAAGAATGGTCTAAGAGAGACCAAGAGACAAAAACAGAGATACCTACAGAGAGAATAATTCTTCTAAAGATAGGGTTTCTATGTGTCTTTGTGTGGCAGAGAGAGATGGGAGTGGGGAAGAATATTCTGGAAATATTCTCTACATCTCAAAATAGAGGGACAGGGAGCAAGAATAGTCTACAGGGTGGTTTTCTGACAGAGACTGAGAGACCAGAAACAGACACACACAGAGATCTGAAGGGAGACACAGAGAGAATATCCATCAGTCTGGCACCAAAGGGTATGCCTGACTTTCCCAGCAATTCTAAACATCACTTCCTATGTTTCCACTCATGTCCCTCAAGAAATTGAAGCCACAATGATGAATACAGTGAAATAATTTAAAGGGGTGAATTTTACTAATGTTATGCTTGCAGTGGCCATTGTCTACTTTGGTCTGTTATCCAAAACATATAAAAGTAAGGACTCAAAAAGATCTGCAGAAAAAGCAAGATGACTTCATTCAAGGCAAGGCTAGAGTGCAGATTACAGACAGACATACTGCCAAAGATGGACAGTTGCCCACTTGACTGACTGGACTCAAGGTCCCCAAAGTTATTAAGTATGTCTCTTTCAGGGGTTCCAAGGAGACTAATGAGTTGGTTAATAGGGGGCCAGATTTATTTTGATTGACAGATTAAAATATTTATTCCTCTAACTGTGTATGTCCCTTCTAGTAATACAGCTGATTTTAATCATACATATGCCAAATTACACAAAGCCATAATGTAGCTAGTAAGGTAAGATCATCTGAAGGACATGTTTTTGTCTTACTATGCATGTACCCAAAACCAGTTCAGGCCAGATGAGCACCTCTATTCTCCATACCAGGATACATTGAGAATATAGTTGAATGGAAACTGTCTGGACTTAATCATTGTTAGAGGTGTATGTCTAGCTGAATGCAGGGTTGGGGACTTTGAGGGGTTCTTTTGTGTACCTTGTTCACAAATAAGTGTGTGAGTATTCTGATGCAGGTGGAGAGGAGGGAGGAGGGATCATTATATGGGAAACTCTACACCATTGTTTAGAGATGGGTATGTGAGTAGCCTGATGCAGTTGGCTCAGGGAGAGGTCTGAAATCATTATTGGGTTCATTGCACAGAGTGTGCATGAACATAAAAGAAGCTAAGCTGCCTAATGCCTTATTACATAATCAGGTTATGAATTACCTCATCCAAGTAGGTTTTGAATCATGGGTTACTAAACTATCCCCTACTTTTACCTGCCTTGTTTCCATAGCTGCATTTTTTTCCAGATTGACGGGGATTGGCGAACAGTTTACTTAGCTGCCAGTACCAAGGAGAAGATAAAGGAAAACTCACCATTGAGGACCTACTTCCGTCACATTTCGTGTAGGAGGAAATGCAACAGAATAGACCTCTATTTTTATATTAAGTAAGATATAATACAGTAGACAGAACAATCCATGTGACATTATATGGGTAGAGAAATGAAGCATTTTCAGTCAACACTCATGTCAGTGAACACACACAGTGAGGATTTGTATATCACTTTACTCTTTATGGTCCTGTAAACAACACATGATTGCTGTACTTTTAATTTAGAATGAGTGAATTTATTTAGAGAATAGCAAGCCAAAGGAAAATAATAAATATCAAGAGATGGATGGTTATTTTATATATATATATATATATATATATATATATATATGTGTGTGTGTGTGTGTGTGTGTGTATAATAGATACATAGATAATATATATATATATAAATAGATAAATGATACATATACACAAATGTAGGTAGTTAGATAATAGATAATTAGCTAGGCAGATGATAGATATATACATAGATATGTAGTAGATAACAGATAATAGGTAGAGAGGTGATATATAAATAGATAAATAATAGATACATGCATGCATATACATACATACATGCATACATACATACATAGGTAGATATAGATATATAAATAGACATATGATAAATACATCTATCTCTGGTGATCAAAGAACACCATGAAAGAAACAGACTTAGGTGTAAAGTACAAGTGGAAAACATATATTCAAAATCAGCTCCTTGATGAAGTCAAATATCACAAGACAATTTACCAAAATAACATAATTTATAATCACTTAATTTTCTATTTAATCACTCCTCAAAAGCCCAGGTTGGAGAGAAGATATATCAAAATAAATGTTGGGACAGTTTCAGTGCTTTTAAATTATCCTGATATGAGTAATATTATCCATCAAGGCTATATTTCTATCTGGAATAAAGGAAATTTTTGTTAATTATGTGCTGATATTTGAACCTTTTCATAAATTGATTATTTATAAATCTTACCCTAACAAAGACACCTGCAAACACTTAACTGGGTGATTGATGACATTTAAGGATGTTATTACTTATGTTTAGGAAAGATGTCCTACAACAACATATACTTATGACTGATCTACAGAGGAAATGTGGTTTGCAGTAAACTGTCAAGTCATTTATATAGAATCAGGACCCTGGAGTAATAGACAATGTGACAACATGCACGCATTTGTTTTTCAGGAAAGGGAAAAAATGCCAACAGTATAAAATTGAAGGAAAGAAACAACAAGAAGTTTACTATGCAGACTGTGAGTAAACAATGCAGGGTTAATGCATGACCTTGATTGGTTTTGTTGTTGTTGTTCAAAATTCACAGTCTTAGTTGAATTTTGCATATCTGAATATCTACATTCCAATGTAATTGTCTCTACAAGTTACAGATGCAATCTTGAAAAAAGCCTCTTCATTTTCTTCCTAGTCATTTTGGAACTAATAAGAGATGTGGTGTGTTTGTTTAGTTTTTAATGATTTTAAAAACTTGTTTATTTATATAGTATATGTCTGTATGAAGCATACTTCCCTTGTTTAGCTTTAAATGGAAACTAAATCACAGACAAGAGTCTGAGAATGGTTAAAGGAATTAAAAGGTGATTTGAGTACTTCAAAAAATAACAATGCAAAATAGGAACTAAGATTTTATCAACTTTTAAAATGACTCCCAGCAATTCTGAAAGATGAGTCTTGTGTATGCAATGATGAATAATCTAAATTGTATATATTTGGGGGGAAGAAAAATTATTTACAATATATAAATTTAAGCTTCATGAATAATCTTATCTCTTTAGTTCTGACAGGCAAGGCATATGACCTAAAACACACTGAGAACCATTTATAAATAATTGAGGTATCAGTTTAACTTGTCATGAAACAAGTGGTAAAGATATTTTCATTATTTTATATTCTACCATAAAGAGTCTATTTAACCCAATATATTAAACTGATAGACTAAAATGTCATGGACTCTAGCTTGATTTATAGATGAGTTTATTGTACTTAAATACAGGAACAGAATCATCTCACAAGAATATATCCCTCCAAAGAGCCCCATATTCTCTCTTATGTGTTATGTAATTTAGTTCAAGGCCAGCCTATTCTACAAAGCACATTTCAGGCCAGCCAAGGATACATAGTGAGACCCGTCTTAGAAGGAAAAGGAGTTACCACTGACATGAAAAGGAACAGGAATAGGGAGGATCTTGGGTCAGCCTCTTCCCCTCTTCAAGAAGTCTTGCTAGTGAACCAGTGAGTGTATTAGAGTCACTTATAAGAGCATGTGTAAATATAGCTTCATTCCTGAAGAGTCCAACCCAGCTTGGATGACACTCCTGAAAGCTTTATTCTTGGAGCTTTAGCAAGGCTTACATATAGCTAAGCTAGTCAGAGTCTCCTCCACAGGGCCTGTTCACTTGGTCTTCCATAACATTGTGGAGTGACCTACTGAAAGTTATTATTCTCAAAATCATCCTGAGACTTGTGGGTTTTTTACTTCTCTAATTTCATATAATCATACAGCTATTCTCAGCAAGTAAGAGGATTGCAGTATGCCACCTTCTATACACTCACACATCTTTGTATCAGGATAATCGCTACCCCAAATCAATATAGGTTCTGCATATTTGTAGGTTAACTGTGGGATATTAGATATATTTTATATCTTAGAACAGAATTATAAACTTGCTTTATAATTCCCTGTTTGTCTCAACTAGTTTCAATATATGGAGCAGAATATTGTGGGTCAGAGATTGCATACTCCATATACCCATTCATAACTGTGCAGTTAGAATGATCCTAAATAATCATTTTGTTGACTTTGTATGTAGTTATACAAATCTCTTATCTATCACAGATGAAGGGATAACAGCATTCATGTTAAAGACTGTGAATGAGAAGATATTGCTGTTTCATTATTTTAACAAGAACAAAAGAAACGAAGTTACACGAGTGGCTGGAGTTTTGGGTAAGTGTCACACATGGAACTTATCATCTGAGTGTGTGGTTCAAGGATATTAATGTATGCATATCTCTGTATCCAAAGTCAGTGTGTTTGTGCATCTGATTCTGTTTGTCATGTTGACAAATCACTGAAGCTGTGATGGTTAGCTTTGAAGATCTACTTGGCACAATCCAGAATGCCATACGAAGGGTCTCAGTAGGAAACTTCCCACAGTAGACTGACCTGTGGATGTGTCTCTAATGATAATTAACATAAGAAGTCTCTACCCAATGTGGGTGGTACCATTCCATAGCAAGAGGACATGCTGTAAGCTTCAACTACCAACAGACACAACTCAGAATCATCTGGCAAAGTTCTTAGGGAGGGATTTCTCTCATGAGACTGGCCTGTGTGCCTGTCACTGGGAGTATACTGAAAGATGATTACACAGGTGGCTCTGCCCACTCTGTTCAGTAGCATTTGCTGGGCAGATCATTCTGACCTGCATCAGACTTGGAAAGCTGGATAAATGCAAGCACACATATGTATGCATTCTTTGATCTCTGTTCCTCACTATAGACATGGTGTGTCTGCCCCCTAAACTTGTTCTGCTGGAGTTTCCCCAATTTAATGTGCTATAACTGGGAAAATAACCCTTTTCCACCATAAATTGATTTTGTCAAAGCATTTTGTCAGAATATTGAGCAAGAAAATTGAGATATTGATAAAATTGTCAATATTGAAAAACATGAGAGATACTATGCTACACATCCTGGGATCCACCCACTACTTTGATTTCTCAGGAAAATGATGCAATTTTGCATCGTTAATCTATTGGAGAAAGGGGGACAATGCTGTGCTTTGGCACTTTTTAAGAGACTAAGCAAAAAAGGATATTTTATTAATAATGTGCTGGGTACTAACGTTGTGACATCTTCCCTATACACCACGAAATGCAGCAAAAAGTGAACGACTGACTAAGGATGAAATGACGGAGTACATGAACTTGGTGGATGAAATGGGCATTGAGGATGATAATGTACTCCGTGTCATAGAAACAGGTATAGCAGCAACCTGTGTGTATAACTTCTCACTTTGCATTTTTATAATGAATTTTATTGTTTTATTATTGAGGGTGTTGGGAAGATGGCTCAGTAGGGAAAGTGCACACATGCATGAGAACCCAAGTTTGTATCCCAGCACCCATTTAGAAAGCTAAGAATAACAGAGCTGGAAAAGGGAGGGGACAAGAGGAGCTTTCTAGTAATAGATCTATCTGAAAGATCTTGTCTCAAAACTAACAATGTGTACAATGAATAAAGATGTTAAAAAGTCAGCCTCTTACCTATGCATGTATGGGTATACACATGTCATTCAAATGACTATAGTCACACCAATGAACCATGCAAATAGATACACACACATAAAATCTACCTGAAATGAAACTCAGTTCCTAGGATCCTCTGTTCAGATACTCTATTCAAAAACTAGCTGTTTTTTATTGGATATATTCTATATTTACATTTCATTTCCCTTTTTTGGTTCCCCACCCCTCAAATGTCCCACGTGCCCTTTTCCCTCCCCCTGCTCCCTCATCGGCCCCTTCCCACTTCCCTGTTCTGGTATTCCCCTACACTGATGCTTTGATCCTTTCCAGCACCAGTGTCCACTCCTTTCTTCTTGTCAGACCTCACTTGGTATGTGATTTATGTCTTTGGTGTTCCAAGATTCTAGGCTAATATCCACTTATCAGTGACTGCATACCATGGGTATTCTTTTGAGACTGGGTTACCTCATTTAGTATGATGTTCTCCAGCTCCATCCATTTCTCTAAGAATTTCATGAATTCATTGTTTCTAATGGCTGAATAGTACTCCATTGTGTATATATACCACATTTTTTTTTTATCCATTCCTCTGTTGAGGGACATCTGGGTTCTTTGCAGCTTCTGGCTATTATAAATAAGTCTGCTATGAACATAGTAGAACATGTATCCTTATTGCATGCTGGGAAATCTTCTGGGTATATGCCCTGGAGTGGTATGGCTGGGTCTTCAGGAAGTATCACGTCCAGTTTTCTGAGGAACCGCCATACTGATTTCCAGAGTGGTTGTACCAGCTTGTAATCCCACCAGCAGTGGAGGAGTGTTCCTCTTTCTCCACATCCTCGCCAACACCTGATGTCTCCTGAGTTTTTAATCTTAGCCATTCTAATTGGTGTGAGGTGAAATCTCAGGGTTGTTTTGATTTGCATTTCTCTAATGACTAATGATGTTGAACATTTTTTAAGGTGTTTCTCAGCCATCCGAATTTCTTCAGCTGAAAATTCTTTGTTTAGCTCTGTACCCATTTTTGGATAGGGTTATTTGGTTTTCTGGGGTCTAACTTCTTGAGTTCTTTGTATATATTGGATATTAGCCCTCTGTTGGATGTGGGGTTGGTGAAGATCTTTTCCCAATTTGTTGGTTGCCGATTTGTCCTTTTGACAGTGTCTTTTGCCTTACAGAAACTTTGTAATTTTATGAGGTCCCATTTGTCTATTCTTGATCTTAGAGCATAAACAACTGGTGTTCTGTTCAGGAATTTTCCCCCTGAGCCAATGTCCTCAAGGTTCTTCCCCAGTTTCTTTTCTATTAGTTTTAGTGTGTCTGGTTTTATGTGGAGGTCTTTGATCCACTTGGAGTTGATCTTAGTGCAAGGAGATAAGAGTGGATCAATTCTCTTTTTTCTGCATGTTGACCTCCAGTTGAGCCAGCACTATTTGTTGAAAAGGCTATCTTTTTTCCACTGGATGTTTTCAGCTCCTTTGTCGAAGATCAATTGGCCATATATGTGTGGGTTCAATTCTGGGTCTTCAATTCTATTCCATTCATCCACTTCTCTGTCACTGTACCAATACCATGCAGTTTTTAACACTACTGCTCTGTAGTATTGCTTGAGGTCCGGGATATTGATTCCCCCAGAAGTTCGTTTACTGTTGATGATAGTTATAGCTATCCTGGGATTTTTGTTATTCCAGATGAATTTGAGAATTGCTCTTTCTAACACTATGAAGAACTGAGTTGGAATTTTAATGGGGATTGCATTGAATCTGTATATTGCTTTTGGCAAGATGGGCATTTTTACTATATTAATCCTGCCAACCGATGAGCATGGAAGATTTTTCCATTTTCTGAGGTCTTCTTCAATTTCCTTCTTCAGAGACCTGAAGTTTTTGTCATATAAATCTTTCACTTGTTTGGTTAGAGTCACACCAAGGTACTTTATGTTGTTTGTGGCTATTGTGAAGGGTGTTATTTCCCTAACTTCTTTCTCAGCCTGCTTGTCCTTTTAGTATAGGAAGGCTACTGATTTGCTTCAGTTGATTTTATAACCAGCCACTTTGCTGAAGTTGTTTATCAGCTGTAGGAGTTCTCTGGTGGAGTTTTTTGGGTCACTTAAGTATACTATCATATCATCTTCGAATAGTGATAATTTGACTTATTCCTTTCCAATTTGTATCTCTTTGACTTTCTTATGTTGTCGAATTGCTCTAGCTAGTACCTCAAGTACTATATTGAAGAGATATGGTGAGAGGGGGCAGCCTTGTCTAGTCCCTGATTTTAGTGGGATTGCTTCAAGTTTCTCTCCGTTTACCTTGATGTTGGCTATCGGTCTGCTGTAAATTGCTTTTACTATGTTTAGATATGGGCCTTGAATTCCTGTTCTTTCCAAGACTTTTAGCGTGAAAGGATGCTGAATTTTGTTAAATGCTTTTTCAGCATCCAATGAGATGATCATGTGGTTTTTTCCTTTGAGTTTCTTTACGTAGTGGATTACACTGATGGATTCCCATATATTGAAGCATCCCTGCATCCCTGGGATGAAGCCAACTTGATCATGGTGAATTCCAAGATGGTGGCGGGTCTCAGGGCTCTGTCTAACCACTGGATTCCCTACAGGACTGGAGGTTCCCTGGTTGGGCTGCTAGAACCTAGATGGTGGTGCTCTCAGTCCACAGGGCTCTGTCATGTTAGCTGTCTGTGGTGTCCTGAGGCAAGATGTCAGTCTCTGGCTGTACGCCAGGCACCGACCCACTAGCAGGTTGGTGCCCACGTGAGTTGGCGCAGAGACTGCCTGCCAAGCTGATCTGACTCTGTGTGCACTGCGAGCTGTTGGTGCACAGACCTCTCGCCAACCTCCTTTGGCTCTGGGCGCAGGCAAGAGCCTGACCAGGTGTTCGTTACTAGGCCAAGTCTCTGTCCACGCACCTGAACCCAGCCATCGGCAGGCAGTACACAGTGACCTGAGGGAAAATGGCAGCCGGCTGCAAGCCTGCGAGCCAGTCCCCAGCGATCAGGGACAGCCTCTGAGCTGCAGGTACACAGAACTCCTGCCAACCTCCTTTGGCTCTGGGGGCCAGCAAGGGCCTGACCAGGTATTCGTTGCTAGGCTAAGTCTCTGTCTACGCACCTGAACCCAGCTGCCGGCAGGCAGTCCGCAGTAAAACCTAGCTGTTTATCTGCATTTTTATGAAAACTGTTCATATGTAGTAATGTTATTATTAATATTATGTTGACGTGCTATATTACTTTAAAATAATACTGTTGTTTTAAAATAATGCATTACTTTAAAATAATATTATTATATTACAAATAATATTATTTTTCCATGTTTACTCTCCTTTAGACACCTGTCCAAGCACGATCAAACTTTAATGACTCTACAAGATTAGGATTATGTAAGTCAAAGCACGTTCATTTTATATCTTGAAGTTTAATTTTAGTTCAATTTTTAAAATAATATTTTATTTGTTCTTTAACATTTTCATATAGGTATATAATGTATATTGATCTTATCTACCCCCCACAAACCTTATCCCAGAGACCCAGCAACACATTTTCCTCTATATTTCATGGCTTTTTAAATTTATCATTATGATTGTTATTATTATTGTTATTATCATCATCATCATCATCATCATCATCATCATCATCTCAATGAGCCAAATTGGTGCTATTTCTGAACAGGATGTGTCAATTCCTGGAAGCATAAGCAATGTAATATACCAGGGGCCACAGCCATAGAAAAAAGTAGACTACATGGAGCTGGTGGCACATGCATTTAATCCCAGCACTCAAGGGGCAGGAAAAAAAAGCATATTTTAGAGTTCTAGATGAGCCTCATCTACAGAGTGAGTTCCAGGGCATCAAGGGTTACACAGAGAAACCCTGTCTCATAAAACCAAAAGGAATATTTTTGATAGATATATTCTTTATTTATATTTCAAATGTTGTCCCCTTTCTTGGTCCTCCCCCTCCCAAAAATCCTATAAGCCATCTCCCCTTCCACAATCAACCCTTTCCCACATCTCTGTCCTGGTATTTCTCTACACTATGGCATCAAGTCTTTCCAGGACCAAGGGCCTCTCCTCCCCACAACTTTGGAAAAGATCTTTACCAACCCTACATCCTACAGAGGGCTAATATCTAATATATACAAAGAACTCAAGAAGATAGACTCCAGAGAACCAAATACCCTATTAAAAATGGGGTACAGAGCTAAACAAAGAATTTCCACCTGAGGAATATCAAATCGCTGAGAAGCACCTAAAGAAATGTTCAACATCCTTAGTCTTCAGGGAAATGCAAGTTAAAACAACCCTGAGATTTCACCTCATACCAGTTAGAACATCTAAGATCAAAAACTCAGGTGCTGGCAAGGATGTAGAGAAAGAGGAACACTCCTCCACTGCTGGTGGGATTGCAAGATGGTACAACCACTCTGGAAATCAGGCTGGTTGTTCCTCAGAAAACTGGGCATGACACTTCCAGAGGACCCAGCCATACCACTCCTGGGCATATACCCAGAAGATTCCCCAGCATGTAATAAGGATACATGCTCCACTATGTTCATAGCAGCCCTATTTATAATAGTCAGAAGCTGGAAGGAACCCAGATGTCCCTCAACAAAGGAATGGATACAGAAAATGTGGTACATTTACATCATGGAGTACTACTCAGTTATTAAAAATTAATTCATGAAATTCTTAGACAAATGGATGGAACTAGAAATGTTTTTAAAGACAGAAAAAGAAGGAAAGAAAGAAGAGAGGGAGGGAGAGAAAAAGAGACAGAAACAGACAGACAGACAGACAGACAGACAGAGGCAGAAAGAAGAAAAAATACAGACCCCTCCTCTCCCAGAAATTATCAACTGGCAATAACTCTTTGGTTAGTGGTCCTGAGTCCTTCTCTGCTCCATTCTAGAATGTTAACTGGCTTGATCTTGTCAGGGTCTTGTGCAGCCCACCACAGTTGCTGTGAATCCATTGTTGTAACAGGTCTGCTATGTTCAGAAAACAGAATTGTATGACTCTACTCCCCACCCAGATGACGATCAATGGCTGAGCATCCACAATCACTTGTCCTCGGCCCTTTGACCAGCTACAAATTTCTGCACGAACCACCCCCCACTGTAAAAAGTTGAGCAGAAGCCAGACATGGAGGCATATGCCCTTAATCCCAACACTTGAGTGGCAGAGGGAGATGAATTTCTGTATCAGGCCAGCCTGATCTATATACTGGGCTCCAAAACAGCCAGGTACATAATTTCTTTCTAAATAAACAAAAAGATAAAGAGTTAATTTGACCAAAATTGAGAGCAGCATAAATCTATGAGTATTTTTAAGAGAGTGGTTGGAAACATGACAGTTAATTACCACTGGTCTCCTCCATAAGCTCCACGAGCTCCATTGTCATGTGCTTTTGACTAGAATTACAAAAGGAGCCCACCCCTATCCCTGTTCTTCCCTAGATGTGAGATCCCTTACACAGAGCTGGCATCAAATTTAATCACAGAGTGGTTGGTTCCCCAGTACAGCCTTTATTGCATCAGTGAATACAAGGATGCTTTTATAGTGTGTTGGGACAGGATGGTGACATCACTGATATCTTATTCCACATACACAGCCTATTATGTACATCTAAGCACAGTGGAAGAGTTTCCTAGTTCATTTGAAATTGATTTCTTGATGCCCTACATCCACAACATGTGGTGTCTTCAGCAATAGTGTGCTAAGTGGTGGTGGATAGCCAAGAGGTAGGGCAATAGCCAAGTTATTTTGGTGATCCCAAGGCCTCCCTCCACTAATAAATAATATGGTGGTACCCCTATGACTAAAAATTCGATTTTCACTGAATAAACCATGTCTTCTAAGAACAGCATTGTACCATTGCAGGGAACCTCTGCTGAAACTTTTTATTTACTATATTTTTAATTTACAAACTAGTAGATTTCTGTAAGACTTCATATACCTTCAGTTTTGATGTTCTTCCCTATGCCCAACAACATCCATACCTGCTCCTCTAACCCACAGCTCTCCCCCTCTAATCTTCCCTGTCACTAGTGCCCAATTACATCACCTGTCTAATTATATTTTACTCACACACTCATAGGGTTCCTTAAGAGTTTTTAATAACCCTTCATTCTGGTTAAACCTTCCACCACACCCTGATTTCCCATTCTACATCCAACTCATGATAGAACCTTCCTACCCCAAGTATTCTTCTTTATACTTCACGTTAATGGCATTTCACTTGATAGACCCACTCCCTTGATGGGCCCAGTTCAAACTGGTTTCTAATTACCTGGCTTCTTCACATACTTCATATTATGCAGACAAAATAAAAGATTCAAATCTAAGATCCATATATGAGATAGAATGTGCAGAGTGTGTCTTTCGGAGCCTGGCTAACCTTGTTGAGTATAAGAATTTCCAGTTGCTTCTATTTACTTGGAAATTTCATATTTCATTTTTCTCTATGGCTGAGTAACATGCCATCTTAGGCATTACATTTTCCTTATCCATTCATCAGCTGATGAACAGTTAGGTCAATTTCATTTCTTAGCTATTATGAACTTAACTGCAATGAACATGGACATCCAGGGATCTCTGTAACAGGATATAAATGCCTTTGGATACCATATCTAGAAATGGTGTAACTGAGTCACATGGGAAACCTTTTTCTAATTTTTGGTTTGTTTACTTGATGTTCATAGTCTTTGGTTTTTGTTTAGCTTTGTTCTAAGTTCTTTGTATATTGTAGACACTAATCTTCCATTGCATGTGTAGCTGACAAAGATCTCCATTCCCTGAGATACCTGTGCATTTAATTGACAGCTTCCTTTGCTGTAGTTTTTAATTCCATGATATCTGACCAGTGTTGGTTTTACTTTCTTGCTACAAGAATTCTATTCAAACAATCTATACCTGTGTCTATGGTCAACACACACTTCCTGCTTTCTCTCTATCAGCTTCAGGCTACCATGTCTTATGACACAGTCTTTGATCCATTTGGAGTTCAATTTTTTACAGTGTAACAGGGAAAAGCCCAGGTTCGTTTCTCTGTATCTTGATGTCCACTTTTTCCAATTCGGCCCATGTATGGAATTATATATTTTTATGTTAGGTCATTTTCCTTCTTTAACTGGAGACATTGACAATATCCAGCAGAGGACTAAGTCTTACTAGTGTTGGCATTCATCTCTATGGGGCTGAAATTGAGGACAGCAAGTTCTACCCCAGTCTTCCTCTGAGATTCAGTTACTATCTTGGACCTCGAGTGAGACTCTGGCTGTAAGACACATCGGGCTTGGTAAACTCCAGTGTGAAAATATCTTGAAACAAAAAGATATATAATTGTTTTAGATTCATACAAACTACATCCCCAAATAAATATGTAAGTTCTAAAAATTACCAATTTAGGTCTTGAAATAAGATCATTTGTCATATTAAAATTTTCTATATATGGAAAACTTCTATATAAAATTCATGTATATTCCCAAACATACAAAATCTTGTAAAATGTTTTTGCATGAGGCATCTTGTCATTGTTTGCCTCTTTAATGGCTTGTGTTTGTTTTATTTTCCACTCTCATCAAATATCATGTGTTACTATCCTAAATATATGAAGTAATTCTGTTCCAACATTACAGATGACATCAGGAATTTTCCAGTATATTCTTCCTGGAACCTGAAACATCAATATGAAGATGAAGCAGGCTTTTCTCTCAGATCATATCTTCCTATTTACTGTAAATTACAATTCCTGTCTCCATACTTTCTCTTTCATTTGTTCTTTCCCATGTTCTAATTGGTGTTAGTGCATCTTTGAATGTTTAAATAAATTTATTTCACTTGCATATGTGTCTTTGAAGAAAGTAAGCTAAAGTGCAATGCACATAAATATCAATTTGACTTTTTAAAAGGAGAAGAGGGTTGGAGGGGTGGCTCAGATGTTAACAGTACTTGCTCTCCCAGAAGACCTGAGCTCAGGTCCAGCCTAGACTACCTGAAATATCACAGGCCAGTGAGAGACCAGCTCAAAAAATAAGGCAGGGAACATGACAGATGGCTTAGTGGTCAAGAGCACTAGCTGCTCTTCCAGAGGGCCTGGTTTGGATTCCTAACACCTACATGGCAGCTCACAACTGTCTGTAACTCCAGTTCCCATGGATCTGACATGTATGTGGACAAACATGCAAGCAAAACACCAATCCACATAAAATTAAAAATAAATAAATAAATTCAAATAAGGCAGATTGCATGAAGAATGGCACCTGACTCTGTCCTCTGGCCTTCGATGTTCACATGCACAAAAGTGAGTCCACAAACATGGGTGCAAAATTACATTCACTCACACACACACACACACACACACACACACACAATCACAACATACATCAAAGAGTCTTGTCATTCCTGCATGATACAGACTCCAATATTATAATTTTGACTTTAGAAAGGAATGTAAATTATATTGGTTGCCTAAAGGAAACATTAAAAGCTTCGTGTGCTGGTATGTCAACTTGGCTCAAGCCATAGTCATTTGGGAAAGGAAACACTCAAGAAAATACCTCCATCAGGTTCAACTATGGGGCATTTCTTGATTGGTAGTCAATGCAAGGGCAGGTCCAAATCACTGTGGAAAACACTGCTGATCAAACCATGAGGAGCCAGACAGTAAGCAGAACTCCTCCATGGCCTCTGTATCAGTTTATGCTCCCAAGTCTCTGCCTTGACTTCCCCAGGTGATGAACTACAAGCTATCAGATGAAATAAACCCTTTTCTCCCCAAGGTGCTTTTGGCCATGGAGTTCACAAAAGCAATAGAAACC
