# Supplementary material for: Comprehensive Analysis of Rodent-Specific Probasin Gene Reveals Its Evolutionary Origin in Pseudoautosomal Region and Provides Novel Insights into Rodent Phylogeny
Source: Biology (Basel). 2025 Feb 27;14(3):239. doi: 10.3390/biology14030239 (PMC11940140; doi:10.3390/biology14030239)
Supplement: Supplementary file 1 [file biology-14-00239-s001.zip › Suppl Data Files/gPBSN/gPBSN_Arvicanthis niloticus.docx]

>JAAOMG010001595.1:c35057000-35042000 Arvicanthis niloticus voucher T564M isolate mArvNil1 chromosome X, whole genome shotgun sequence

CATTGGGCATTGGAATTGACAATAAAAGTCAATTTTCCATTCCTAGTAGAGTTCTCCAGGAACCTATTTGTATACTAAATGACACAATGTCAACGTCAGTGCACAACTGCCAACTGGGATGCAGGACACTGCTCATGCCAACCATCCTGAAAGGCAGCTATAAAAAGCAGGGAGTTATTCTGCACCTTGTCAGTGAGGTCCAGATACCTACAGAGCAGACACAGTTGCTCACACATGATGAGGGTCATCATCCTCCTGCTCACACTGGATGTGTTAGGCGTCTCCAGTGTGATGATGGAGAAGAGACTCAAACAGAAGGTAGCAGGCCTTCAAGGAAGGGGGCTGTATGTGGGGGGCATGCTGGGCAGATACAAACAGAGAGACAGAGAGAGGCTTGGGAGGGGGATTTTTGCAGGTGGGGGTGGGCAGCAAGAGACAGAGAAGCAGAGGCAGAAATACCTTAAAAGGGGAAAGAATGTTCTGGAAGTTAGTGTTTGCTAAGTCTGGTAATGGGGAGTGACAAATGTGTTGGGAGGAGAGTAGTTGGGTCTCATAAATGCTTTTCTGAGAGAGAAAAAGGGACAGATACCAAGAGATAGAGACAGAGAGAAATGAGACAGTTTCTGGAGAATTTTGCTAGGGATGGATAATTACCCTGTTGGTATTCTCTACATGAGGAGTGGAGAGAGACAGAGAAAGAGAAGGTGGTCTAAAAGGTGGGTTTCTGAGATAGAGTCACAAAGAGAGGGCAAGAGACAAAGACAGAGATACATGCAGAATAATGCTTCTGAAGATAGGTTTTCTGTGTATCTTTGTGTGACAGAGAGAAATAGGAGTGGGGAAGAATGTTCTGGACGTATTCTCTACATCTCAGAGTGGAGAATGGCAAGGAGAGAGAATAGTATAAGGAGTGGTTTTCTGAGAGATAGAGACTGAGAGACCAGAGAGGCAGACACACAGAGAGACCTGGAGAGAGACATAGAGAGAACATCCATCAGTCTGGCACCAAGGGTGTGTCCAGCTTTCCCAGCAAATCCAAACACTTACATTGCTTCCTATGTTTCCACTCATGTGCCTCACCCTATGAAGAAATTGAAGCCATTACAATGAATATGATGAAGTAATGTAAATGGGTGAATTGTACTAATATTATGCTTGCAGTGACTATTGTCTACTTTTGCCTGTTATCTAAAATATTAAAAGTAAGGATTCAAAAAGATCTCAGGAAAAACAAGATGACTTAATTCAAGACAAAGCTAGAGTACAGATTACAGACAGACATACTGCCAAGGATGGACAGCTGCCCACTTGACTGACTGGACTCAAGGTCCCCAAGATATTATGTATGTCTCTATCAGGGGTACCAAGGAGACCAATAAGCTGGTTACTAAGGGGCCAGATTTATTTTGATTGACAGATTAAAACTTTTATTTCTCTAACTATATGTATGTCCCTTCTCATAATGCATCTGATTTTAATCATACATATGCCAAATTACACAAAGCCATAATAGGTAGGGTAAGATCATCTGAATAACATGGTTTTGTCTTACTATGCATACACCCCAAACCAGTTCAGGCCAGATTAGAGCTTCTACTCTCCATACCAGGATACATTGAGTATATAATTGAATGGAAACTGTCTGGACTTGATCATAGTTAGGGGTGTGGAAAGTCCTACATCATTCTTCAGAGACCGGTATATGACTAGACAGATGCAGGATTGGGGACTATGAGGGGTTCTTTTGTGTACTCTACAGCCTTGTTCACAAATCAGTGTGTGAGTACTCTGATGCAGGTGGGGAAGAGGAAGAAGAGGTTGTTATATGGGGAACTCTACATCATTGTTAAGAAAGGGGTATGTGAGTAGCCTGATGCAGTTGGCTTGGGGAGGGGTCTAAAATCATTATTGGGTTCATTGTGTAGAGTATGCATGAACATAAAAGACGCTAGGCTGCCTTATTACATATGCCTTATTACATAATCAGGTTGTAAATTACCCCATCCAAGTGGGTTTGGAATCCCGGGTTACTAAACTATCCCCTACTTTTACCTGCCTTGTTTCCATAGCTGCTTTTTTTTCCAGCTTGAAGGGAAATGGCGAACAATTTACTTAGCTGCCAGTACTGAGGACAAGATAAAAGAAGGCTCTCCATTGAGGACCTACTTCCGTCTCATTTTGTGTGGGAGAAACTGCAACCAAATCTACCTCTATTTTTTTATTAAGTAAGATATATTTAGAGACAGAACAATCCATGTGATGATCTGTGGGTAGAGAAATGAAATGTTTTCAGTCAACATTCATGTCTGTGAACACACACAGCACTAAGTGAGGATTCTTATATCATTTTGCTCTGTATGGTCCTGAAAACAACATATGATAACTGTACTTTTAATTTAGAATGAGTTAATTTATTTATTGTCGATATTTACTATATATACATAGCTACCCAACTATGTATGTATACATGCATATATCCATCTATTATTTATCTATTAATATATCTTCTCTCTATCATGTTATCTGTCTACCTACATACCTATGTATATATCTATCTTCCATCTAGCTAATTATCTATTATTTAACTACCTACATTTGTGTATGTTTATCTTCTATATACTTTTAAATCTATTATCTATGTATCTATCACCTATATATAATATCCACCCATCTCTTGATATTTATTATTTTACTTTGGCTTAGTATACAATAAATAAAAGAGCAATAGTTTAAAATCAAATAGGTGTATACTCTAGCATTATGAAGAGATAAAATTTTTATTTTTAAATACATTTTTTAAACTAAATCATTACCTCGTTTCTCCCTTCCATTTTCTCCCTCTGATCCTTCCCATGTACCCTCCTACCCTCCTTACTTGGTTTCTTTCAAATCCTAGGCTTCTTTTTTTTATTGTTTGTTTGTTTGTGTGTGTGTGTGTGTTCTTAAACATACAACCTGTTCATTCTATATAATGTTACTTGTATAGATATGATTTTTACAGAAACCATTTGATATTCAAAAACCAATTGATAGGAAAGACTTTTCTTCAGGGAAGACTGCAAAATATCCTAAAATTCATATGGAGCCAGAAAAAAATCTTATGGTAGCTAAAACAATCCTGAGCAAAAGGAACAGTGCTGGAGGGATCGCCATTCCAGACAAGAGTGATTTTCTTTTTTACATGCAGAGAATAGACCCCAAGGCTTTCAATGGGCTTTATGTAACCACATCCCTGATCTAAACAGTTATTTCCATCTTTTTTGCTTGTTTTTGGATGTGTGCCTTTAATGGCTGAGTCATCTCTGCAGCCCAGTTACTCCTTGTTTTTAGAGCCATAAAGACAAATCTAAGCTGAGACTGTCACTATTAGTCACATAGATCTCTGATGAGTTGAATACAGATCTGAATATATGACCATGCCTACGACTCTATGAATCTCCTGATCTGTCGATATACATCACAACTTAAATCTTCTCAGAATAAATTTGTAGGCCGAATTTGAATCCATTTAAATGCACAAAGCAATTGCTGTTACAGAATCTATAATATTGGTGACATTTATGAGAGAGGCAAAAAATGAATACATGGTTCCAATGTAAATATGGAGGCTAGACTATAGTGTATGTAGGGCACTGAGATGTGCTCTGCACCAGGAGGCCTTGCACGCTTTAAACCCAATTTCTACAGACATTTCTCATCTCAGATTGGGGTAGAATTTTTCTCAATACTTTGGGTTTTTTTTTTTCATTTATCAATAGTCATTATTGGGTGCTTGGGATACAGCTGTGTTGAGAAAGTGATTTTATAGCATGAACCACAGAGCCCAGGGTTCCATCCTCAGCATAAAGTTGCAGTGCTACATTCCCATCATTCAAGCACTTGGTGAGTAGAAGCAAGAAGATCAGGGGTTCAAGGACATTTATTGGTCATAGGTCAGGATAGGCAACAGGTATCACACAGAAGAGCTAATTCTGCCCTAACAGAGATGCCTGCAAACACCTAACAGGGTGATTGATGACATTTAAGAATGTATTAATTATATTTAGGAAAGATGTCCTACAACAACATGTTCTTATGACAGATCTACAGAGGAAAATGTGGTTTGCAATAAACTGTCCTGTCATTTATGAGGAATCAGGACTCTGGAGTAATATACAACACAACAACATGCATACATTTGTTTTTCAGGAAAGGGGCCAAGTGCCAAGAGTACAAAGTCACAGGAAGGAAAGAACAAGAAGTTTACTTTGCAGAATGTGAGTAGACAATACTGGGGTGAATGCATGACCTTAATTGTTTTTCTTTTCTCAAAATTCATGGTCATAGTTGAATTTTGCATATCTGATATCTACATTCCAGTGCATTTGTCTCTACAAATTACAGATGCAATCTTGAAGAAAGCCTCTTTCATTTTCTTCTTAGTCATTTTGGAGCTAGTAAGAGATGTGGTTTGTTTGTTTAGTTTTTAATGAATTTAAAAACGTTTTTATCTACATGGTGTATGTTTGTACCAATCACACTTCCCTTGTTTAGCTTTAAGTGTAAAGTAAATCACAGACAAGGATCTGAGAATGGTTAAAGAAATTAAAAGATGACTTGAGCACTTCAAAAAATAACTTGATGCAAAATAGGAACTAAGATTTTATCAACTATTAAAATGACTTCCAGAAATTCTGAAACATGAGTCTTGTATATGTGATGATGAATGATCTAAATTTTATAATTTTATTGGGGAGAAAATTATTTACAATATATAAATTTAAGCATCATGAATGATCTTATCTCTTTAATTGTGACAGGCGAGGCATATAACCTAAAACACACTGAAATCCATTTATAAATAATTGAGGTATCAGTTTAATTTGTCATGAAACAAGTGTTAAAGACACTTTCATTATCTTCTATTCCACCATAAAGTGTCTGTTTAACCCAATACATTAAACTAATAGACTAAACATGTCATGGATTCTAGCTTGATTTATAGATGAGTTTATTGTACCTAAATAGAGGAGCAGAATCAACTCACAAGCAAATATCCCTCCAAAGAACCCCATACTCCACCTTGTGTGTTATGTAATTTAGTTTGTGGCCAGCCTGGTCTACACAGCAAGTTTCAAGTCAGCCAAGGATACATAGTGAGACCAGACTCAAAAAGAAAATGAGTTAACACTGACATGAAAAGGAACAGTCATCATATCAATGTGGAGGGTCTTAGGTCAGCCCCTTCCTCTCTTTAAGAATTCTTGTTAGTGAACCAGTGAGTGTATTGGAGTAACTTACAAAAGCATGTGTAAATATGGCTGCATTTCTGAAGAGCCCAACCCAGCTTGGCTGACACTCCTGAAAGCTTTATTCTTAGAGCTTTAACAAGGCTTACAGACAGCTAAACTAGTCAGAGTCTCTTCCACAGGGCTTGTTCACTCTTTCCCATAACACTGTGGAGTGAGTGACCTACTGAAAGTTGTTATTCTCAAAATCATCCTGAGACTTGTGGGATTTTTACTTCTCAAATCTCATGAGCTACCAAGACAGAATGTTCTGGTTTAGAGAAAATCTATATGTGGCACCCTTTCTTTTTTTCTGTCATGTCTCTGAGGTAAAATTGGTAAAGTTAGCAATTGCACCACTAAATGTAGATCAGATCCATGAAAATGTGTATATATATATATATATATATATATATATATATAATCAGAGAGCTTTCCTCAATATGTGTATATATGTGTAGATAATTCCACCATAAAATGTAATAGGATCCATGAAAATGCCTATATATGTACACGCATATAATATATATATATTACATCTTTTCTCAATATATGTATGTGTAAACAATTCCAGCATTAAATTTAATAGGATCAATAAAATGTATATACATATGTGTGTATGCATATACAGCTATTATCACCAAGTAAGAGGATTGCACTATGCTACCTTCTATGAACTCAGACATCTTGGTATCAGGACCATCATTATTCCAAATCAATATAGATTCTACATATTTGTAGGTAAGTATGGGATATTAGGTATATTTTATATCTTACAACAAAATTTTAAACTTGATTTATAATTCCCTGTTTGTCTTGACTAGTTTCAACTCATAGACCAGAATATTGTGGGTCAGAAATTATATACTCCATATACACATCCATAACTGACGTTAGAATGAACCCGAACAACCATTTTGTTGACTTTATATGTATTTATACAAATTTCTTATCTATTGCAGATGAAGGGAACAACGCATTAGTGTTAAAGACAGTGAATGAGAAGATATTGCTGTTTCATTATTTTAACACGAACAAAAAAAATGAGGTCACACGAGTGGCTGGAGTTTTGGGTGAGTGTCACACATGAAATTAGTCATCTGAGTATGTGGTTCAAGGACATGAATGCATGTATATCTCTGTACCCAAAGGTCGCAGTGTATTTGTGCATCTGATTCTGTTTGTCATGTTGATAAATCACTGAAGCTGTAATGGTTAGCTTTGAAGATCAACTTGACACAACCAGAATGCCCTGGGAAGGGTCTCAGTAGGGAATTTCCCATAGCAGACTGACCTGTGGGTGTGTCTCTGCCCTGTCCAGCAGGGCATTAAACAGGGGTCCGGGGAGGTCACCTGAAAGAGAGGATGGGGGACACAGGTGAACACAAAGAAACGTGGCTTGTCTATAGGTTGATCAAACCGTAGTTTTTACTTTTTCACACAATGGTTATATACACAAAAAGGCAGGATGTGGGGAAGGGGATGATACAGGAGCATAGGTGTTCAGGGGGAGTACAGATATCTTCCAGAACAGAGTGTCAGCTAGGTAAAGGTTCAGGGGACAGGTCACTATGACTCCACCTATGATTCACTTGTCCTTATTTAAGTTGGTACTATCCATCAAGGCTACCCAAGGTCTATGACTATCCACAAGACCAATGCCTACTTGTTCCTATCCAGGCTGGTAAATTTCCACCAAAGCTGCCTGTTTACAGTATCTTAGAGCTATCTGTTTCAGTGTTTTTCCACAGAGACCCAAGACTTTGTGTTAGCAGGCATGTATGTCAGGCCTATTGCTGATTTTAGGTCTATGGCTGATTTTAGGCCTTCAGTGTAAGCAGGACTACCCTTGACATGTCTCTAATGATAATTAGCGTAAGAAGTCTCCACCCAACGTGGGTGGCACCATTCTGCAGCAAGAGGATATGCTGGAAGCTTCAATCACTGACAGACACAACACAGAATCCTTTGACAAAGTTCTTAGCGAGGAATTTCTCTCATGAGACTGGCTTGTGTGCTTGTCTCTGGGAATATACTGAATGATGATTACAAAGGGAGGACTCTGCCCGCTGTTTTCAGTACCATTTGCTGGGCAGATCATCCTAACCTGTATCAGACTGGGAAAGTTGGTTAAATGCAAGCATACATATGTATGCATTCTTTGATCTCTGTTCCTCACTATGCACATGGTGTCTCTGCCCCTAAACTTCTCCCACTGGAATTTCCTCAATTAAATGTGCTATAATTGGGAAAATATACCTTTTTCTACCATAAATTGTTTTTGTCAAAGCATTTTGTCAGAATATTGAGCAAAAAAATGAGATATTGATAAAATTGTCAATGTTACAAAACATGAGAGATAGTATATTACATCCTGGGGTTCACTCACTGCTTTGATATCTTAGGAAAATGATGCAATTTTGCACTGTCAAACTATTGAAAAAAGGAGGCAGATGTTGTGCTTTGGCACTTTTTAGGAGACTAAGCAATAGAGAATATTTTATTAGTGTGCTGAGTACTAATGTTGTGACATCTTCCCTATACACCATGAAACGCAGGAAAAGGCAAACAACTGACTAAGGAAGAGATGACGGAGTACATGAACTTAGTGGAATCAATAGGCATTGAGGATGAGAAAGTACTGCGTGTCATGGACACAGGTATAGTAGCAACCTGTGTGTGTAATTTCTTACTTTTGCATTTTTATAATAAATATGTATTTTTTATTATAGAGGGTATTGGGAAGATGGCTCAGTGGGGAAAGTGCACATAAGCATGAGGTCCCAAGTTTGGATCCCAGCACCCATTTAGAAAGCCAAGAATAACAGAGCTGGGAAGGGAAGGGGACAAGAGGAGCTTTCTAGTAGTAGATCTATCTGAATGATAGTGTCTCAAAACTAACAATGTATAGAATGAATAAAGAAGATGTTAAAATGTCAGCCTCTTGCCTATGCATGCATGGGAGTACCCATGTCATTCACGTGACTATGGACATACAATGAACCATGCAGATAGATACGCACACACAAAATCTACCTGAAGTGAAACTCAGTTCCCAGGATCCTTTGTTCAGATACTCTATTTAAAACTTAGCTTTTTATCTGCATTTTTACAAAAACTGTTTGTATGCAGTAATGTTATTATTAATATTATGTTGAGGTACGGTATTACTTTAAAATAATAGTGTTGTTTTAAATTAATAAATCATTTTAAAATATTATTATATTAAAAATAATATTATTTTTCCATGTTTACTCTCCTTTAGACAACTGTCCAAGCAAGATCAAGATTAAGTGAGTCGAAGCACGTTCATTTTATATCTTGACATTTTATTTTAATTCAATTTTTAAAGTAATATTTTATTTGTTATTTAACTTTTTCATATATGTATATAATGTATCTTGATCTTACCTACTCCCACACACCTCATCCCAGAGACCCAGAAACACATCTCCCTCTATACTTAATGTTTTTTTAAATTTATCATCATCATCATCATTTTCTCAATGAGTCTAAATGGTGCTATCTCTGAATAGGGTGTGTCAATTCCTGGAGATATGAGCAGTGTGATAGGAAAATATGCAATCCATAGGAAAAAATGGACTGCAGGGAGCTGGTGGCACATGCATTTAATCCCAGCACTCAAGAGGCAGAAGCAAGTGTATCTCTGGGTTCTAGATTAGCCTAGTCTATAGAATGAGTTCCAGGGCAACAAGGGCTACACAGAGAAACCCTGTCTCATAAAACCAAAAAAATTTAAAGACAGAAAAGGAAGGAAGAAAGGAAGGAAGGAAGGAAGGAAGGAAAGAAAGAAGGAGGAGGAGGGAGGGAGGGAGGGAGGGAGGGAGGGAGGGAGGGAGGGAGGGAGGGGAAAAGAGACAGAGAGACACAGACAGACAGACACACAGACACACAGACACACAGACAGACACACAGACAGAGGCAGACAGAAAGGCAGAAAGAGGAAAAAAGAAAAATACAGACCCTCCCTCTCCCAGAAACTATCAACTGGCAATAGCTCTTTGGTTAGTGGTGGATTGTCCTGAGTCCCCCTCTGCTCCATTCTAGAATGTTAACTAATTTGATCTGGCCAGAGTCTTGTGCAGACCACCACAGTTTCTGTAAGTTCATGATTTAACAGGTCTTTCATGTTCAAAAAACAGAATTTTTTGGCTCTACTCCCCACTGATGACTGAGCATCCACAGTCACTTGCCCTCAGCCCTTTGACCAGCTACAAAGTTCTGTGTGAACCACTCCCCACTCTAAAAAGTTGAGCAGAAGCCAGACTTGGAAGCACACCCTTAATCCCAACACTTGGGATGCAAAGGTAGATGAATCTCTGTGAGTATCAGCCAGCCTGGTCTATATACTGAGCTCCAGAACAGCCAGGTACATAGTCTCTATCTAAACAAACAAACAAAAAGGTAAAAAGTTGATTTGACCAAAATTGAAAGCAGCATAAATCTATGAGTATTTTAAAGACAGTGGCTTGGAAACATAACACTTTATCACAACTGGTCTCCTCCATAGGTTCCATGAGCGCCATTGTTATGGATTTTTTACTAGAATTACAATAGGAACCCACCCTTACCCCTGTTCTTCCCTAGATGTGAGATCCCTTCTATGGAGCTGGCATCAAATTTAATCAGAGAGTGGTTGGTTTCCCCAATAAAGCCTTTATTGCACCAGTGGATACAAGGATGCTTTTATAGTGTGTTGGAGCAGCATGATGACATCACTAATATCTTATCTCACATACACAGCCTATTAAGTACATCTAAGAACTGAAGAAGAGTTTCCTAGTCCATTTGAAATTGATTTCTTGATGCCCTACAGCCACAGCACGTGATGTCTGCAGTAATAGTGTCCTACTATTTACTTATGCTGGATAGCCAAGAGATATGGCAATAGCCAAGTTATTTTGGTGATTCTAAGGCCTCCCTCCACTAATAAATAGTATGGTGGTACCCTGGTGACTAAAAATTAGATTTTCACTGAATAAACAATGTCTTCTGGGAACAGCAATTGTACCATTGCAGAGATCCTCTGCTGAAACTTTTTTAATGCTGTATTTTTAATTAGCTTACAAACTAGTGGATTTCTGTAAGACTTCTTCATACACCTTCAGTTTTAGTTGACCCACCCCTACCCCTGCTCTTCCTTATGCCCAACCACATCCACACCTGCTTCTCTAACCCACAGCTTTCCCCCCTCTATCTTCCCTGTCATCAGTGCCTGTCTAATTATATTTTAATCACACAATCATTGGTTTCCATATGAGTTTTTATAACCCTTCATTCTGGTTAAACCTTCCATTACACCCTGATTTCTCCATTCCACATCCAACTCCATAATTGAGCCTTCCTGCCCCCAGTATTCTTCTTTATACTTCATTTTAATGGTATTTCACTTGATGGACCCACTCCCTTGATGGACCCAATTCAAACTGGTTTCTAATTACCTGGCTTCCTCACATACTCCATATTATGCATACAAAATAAAAGATTCAAATCTAAGATCCACATGTGAGATAGAATGTGCAATTTGTCTTTCTGGGCCTGGGTGACCTTGTTGAGTATGATAATTTCCAGTTCCTTCTATTTACTTGTAAATTTCATATTTCATTTTCTCTATGGCTGAGTAATATTACATCTTATACATTACATTTTCCTTATCCACTCATTAGTTGATGAACAGTTAGGTCAAGTTTATTTCTTAGCTATTATGAACTTAACTGCAATGAGCATGAACATTCAAGAGTCTCTGTAACAGGATATAAATCCTTTTGGGTACATATCTAGAAATGCTGTAACTGAATCACATGGGAATTCTTTTACTAATTTTTGAGTTTGTTTATTTGATGTTCATAGTTTTTGTGTTTTGCTTTGCTTTGTTCTAAGTTCTTTGTATCTAGTAGACACTAATCCTCCATCACTTGTGTAGCTGCAAAGATCTCCATTCCCTGACATACCTGTGCATTTAATTGACAGCTTCCTTTGTTGTTGTCTTTAATTCCATGATATCTGACCAGTGTTGGTCTTAATTTCTTGCTACAAGAATCCTATTCAGAGAATACATCCCTGTGTCTAAGGTCAACACACACTCCCTGTTTTCTCCTCTATCAGCTTCAGGTTACCAAGTCTTATGACACTGTCTTTGATCCATTTCAATTTGAGTTTTTTGCAGGGTGACAGGGAAGGGCCCAGGCTCATTTCTTTATATCTTGATGTCCACTTTCTTTAATTCTATCTATTTATGGAATTATACATTTTTATATTAGGTAATTTTCCTTCTTTCAGAGGAGACATTGACAATATCCAGAAGGGGACTAACTCTTACTAGTGTTGAGATGTCATTCTCCTCTATGGGGCTGGTATTGGGGGCAGCAAGTTCTACCCCTAGTCTTCCTCTAAGATTCAACTACTATCTGGGACCTTGAGAGAGACTCTGGCTGTAGGATACATGAAGCTTGATAAACTCCAATGTAAAAGTACCTTGAAACAAAAATATATATCATTTTAGTTTAGATTCATAGAAATTACATCCTCAAATAAACATGTGAGTCCTAAAAAATATTAATTTAGGTTGTGATATAAGATCATTTGCCATATTAAAAATTTCCATATATGGAAAACTTCCATACAAAGTTCATGTATATTCCCAAACATACAAAATCCTGTAAAATGTTTTTGCATGATACATCTTGTCATTGTTTGCCTCTTTAATGGCTTGTATTTGTTTTATCTCCACTCTCATCAAATATCATGTATTACTAGCCTAAATCTATAAAGTAATTCTGTTCCAACATTACAGATGACATCAGGAACTTTCCAGTATATTCTTCCTGAAACCTGAAACATCAATATGAAGATGAAGCAATCTCTTCTCTCGAATCACATCTTCCTATTTACTGCAAATTACAATTCCTGTCTCCATACGTTCTTTTTCTCATGTTCTAATCGGTGTTAGTGCATCTTTGAATGTTTAAATAAATTTATTTCATTTGCATACGTGTCTTTGAAGAAAGGAAGCTAAAGTGCAATGCACATAAATATATATTTGACTTTTTTAAAAGGAGAGGAGTGTTGGAGGGGTAGCTCAAAGGGTAACAGAGCTTGCTGCACTTCCAGAGGACCTGAGTTCAGTTCTTAGCACCCACATCAGGCAGCTCACAGCTGCAGTTCCAGGGGACCTGACACCCTCTCCTGGACTCCTTGAGCACTACACACAGATTACAAACACACACACACACAAATAAAAAATCTAATGCAAGTATGGATTAATAATATTAGATGCTTTTTATTAATAATAATTTGAGGAGCTTGGGTAAAGCTTAGTTGTTGAAGTGTTTGCCATGTAAGTACAAATTTTAAATATGTGTTTCAATTCTTAGAATCCACATGAAAAGCCAGGTCTGCTGGCTCATGACTGTAATTTCAGCACCAGGGAAGGAGGTAGAAACAAATGAATCTTCTGATGTCACCAGGAGCCAGCCTAGACTACCTGAAATATCGCAGGCCAGTTAGAGACCATCTCAAAAAATAAGGCAGGGGACATGACAGACAGCTCAGTGGTCAAGAGCACTAGCTACTCTTCCAGAGGACCCAGGTTAGATTCCTAACACCCACATGGCAGCTCACAACTGTCTGTAACTCCAGTTCCCAGGGATCTGATATGTATGTGGCCAAACATGCAAGCAAACACCAATGCACATAAAATTTTAAAAAATAATAAATTCAAATAAGGCAGATGTGCATGAAGGATAGCACCTGACATCATCCTGTGGCCTTCAACATTCACATGCACACATGTGCGTCCACAAACACAGGTGCAAAACTACACACACACACACACACACACACACACACACACACCATACACCAAAGAGTCTCGTCATTCCTGCATGTTACAGACTCCAATATTGTAATTTTGACTTTAGAAAGGAGGGTAAATTATTATACTGGTTGTTTAAAGTGAACACTAAGAGCTTTGTGTGCTGTTAGTTTGTCAACTTGACACAAGCCATAGTCATTTGGGAAAGGAGACACTCACGAAAATACCTCCACCAGGTTCAACTGTGAGGCATTTCTTGATTGGTGGCCATTGCTGTCAGACAAGGCCATCCCCTGCTACCTATGGATCTGAAGCCATGGATCTCTCCCTGTCCACTCTTTGGTTGGTGGTCTAGTCCCTGGGAGCACTGGGTAGTCAGGCCAGCCTATGTTGTTCTTCCAGTGGACTTGCAATCCCTTTCCATGCCTACAGTCCTTCTGCCAGCTCCTCTACCAGGTTCCCTGAGCTCAGTCTGATGGTTGGCTCCAAGCATCCACAACTGCATTGCTCAGTTGCTGGCCAGACCTCCCAAAGAACACAAGGGTCCTGTCAGCAAACACCTCTTGACCATGGCAACAGTGTTGGTTGGGTTTGGTGTCTGCAGGCATGATGAATCACCAGGTGGGAAAGTCTCTGGTTGGCCCTTCCTTCAGTCTCTGTTCCATTTTTATGCCTTTTCTTTCTTTGCAAGGGAACATTTCTGGGTTAAAAACTTTGAGCTGGGTGGGTGACCTCATCCCTCGACCAGGGGCCATGCCTATCTACTGGAGGTAGTCTTAACAGGTTCTATCTCCCCCTTCTCTGGGCATTTCGGCTAAAGTCATCCCCATTGGGTCCAGAGAGCCTCATGTTTCCCTGGTGTCTGGGACCCTCCAGTGGCTATCCCCAGTTCCTCATCCCTCACTGCTACATATTTTTGGTTGATTTCCTGACCTTCTGTACCTCTCTGACATCTCGTCCAGTTCGTGATAGTGCACCCTAATTTCCTCCCCCTCCTGTCTCCATCCAAGGCTTCCCTCTATTTCTCTAATAGAAGAGAAAGTGGGGAAGAGCCTCAAATACTTGGGCACAGGGGGAAATTTCCTGAACAGAACACCAATGGTTTATGCTTTAAGATCAACAATTGACAAATAGGATCTAATAAAACTGAAAAGCTTCTGTAAGGCAAAGGACACTGTCAATAGGACAAAATTGCAACCCACAGATTAGGAAAAGATATTTACCAACCCTATATCTGATAGAGGGCTAATAGCCAAAATATACAAAGAACTCAAGAAAGTAGACTCTAGAGAACCAAATAACCCTATTAAATGTGGAGTACAGAGCTAAATAGAGAATTAATCGAAGAATCTCACATGGCTGAGAAGCACTTAAAGAAATGTTAAACGTCTTTAATCATCAGAGAAAGACAAATCAAAACGATCCTGAGATTCCACCTCACACCAATCAGAATGACTAAGATGAAAAAAAAAAAAAAAACCTCAAGTGACAGCAGATGCTGGCAAGGATGTAGAGAAAGAGGAACACTTCTCTATTGTTGGTGGGATTGCAAGCTGGTACAACCACTCTGGAAATCAGTCTGGCAGTTCCTCAGAAAATTGGACATAGCACTACCTGAGGTCCCAGCTATACCATTCCTGGGCATATACCCAGAAGATGCTCCAACATATAACAAGGACACATGCTCCACTATGTTCATAACAGCCTTATTTATAATAGCCAGAAGCTGGAAACAACCCAGATGTCCTTCAACAGAAGAATGGATACAGAAAATGTAGTACATTTTCATAATGGAGGACTACTCAGCTATTAAAAACAATGACTTCATATAATTCTTAGGCAAATGGATGGAACTAGAAGATATCATCCTGAGTGAGGTAACCAAGACACAAAAGAACAC
