# Supplementary material for: Comprehensive Analysis of Rodent-Specific Probasin Gene Reveals Its Evolutionary Origin in Pseudoautosomal Region and Provides Novel Insights into Rodent Phylogeny
Source: Biology (Basel). 2025 Feb 27;14(3):239. doi: 10.3390/biology14030239 (PMC11940140; doi:10.3390/biology14030239)
Supplement: Supplementary file 1 [file biology-14-00239-s001.zip › Suppl Data Files/gPBSN/gPBSN_Grammomys dolichurus.docx]

>JADRCF010083295.1 Grammomys dolichurus isolate MVZ Mamm 221001 853264, whole genome shotgun sequence

TATTTACTGGCCCTTTAAGTTGGGAATCTTCATTCTCATCTATACCTATTATCCTTAGGTTTGGTCTTCTCATTGTGTCCTGGATTTCCTGGATGTTTTGAGTTAGGAGGTTTTTGCATTTTGCATTTTCTTTGACAACTGTGTCAATATTTTCTACCGTATCTTCTGCATTTGAGATTCTCTCTTCTATCTCTTGTATTCGGTTGGTGATGTTTGCGTCTATGACTCCGGATCTCTTTCTTAAGTTTTCTATTTCCATGGTTGTCTCCCTTTGAGCTTTCTTTATTGTTTCTATTTCCATTTTCAGATCCTCAATGTTTTTGTTTAATTCCTTCTTTGTGTTTTCTTGCATTTCCTTAAGGGATTTCTGTGATTCCTCTTTAAGGGCTTCTATCTGTTTACTTGTACTCTCCTGTATTTCTTTAAGGGAGTTACTTAATTCCTTCTTATAATCCTCTATCATCATTATGAGAAGTGATTTTAATTCTGAATCCTGCTTTTCAGGTGTGATGGGGTGTTCAGGGCTTGCTATATTGGGAGAACTGGGTACTGATGATGCCAAGAAACTTTGGTTTCTGTTGCTTATGTTCTTACGCTTGCCTTTCGCCATCTGGTTAACTCTAGTGTTGCCTGCCCTCACTGTGTAAGACTGGAGTCTGTATTTCCAGTTATCTTACTTGTTGCAGAGCTCCTCAGAGTCCAGATGTCTCTGTAGTCTTATGATCCTAATCTCCTGGGGGAAAGAACTCCTGGGACTCAGGCCCCCTCTTGGATCTGGAAATCCTCCTGCTCTGGGTGCAGTGGATCCTCTAGGATGGCTGAGGATACGATGTCTCCCAAGTGCAAACCACCTTGGTGTCTGCTGCTCTGAGTGCAGTGGCTCCTCTAGGATGACTCAGGAGTCAGTATCTCCACAGGACAGGAGCAGATCAACTCTGTGTCAGCTGCTCTGAGTGCAGTGGCTCCTCTAGGATGACTCAGGAGTCAGTATCTCCACAGGACAGGAGCAGACCAACTCTGTGTCTGCCCCAGCCCACAGGTCCAGGCAGGGGAGGAAGCCCTGTAATTCTTTAAGGGATATTTTGTGTTCCCTCTTTAAAGGCTTCTGCCTGTTTACTTGTGTTCTCTTGTATTTCTTTAAGGGAGCTATTTACGTCCTTCTTAAAGTCCTCTATCATCATCATGAGAAGTGATTTTAAATCTGAATCTTACTTTTCCGGTGTGATGGGGTATCCAGGACTTGCTACGGTGGGAGAACTAAGTTCTGATGATGCCAAGTAACCTTGGTTTCTGCCTATATTGAATTGAGATATGGAGTTCAAACACGTGGCTTAAAATAGCATGGATCTCAATGTCAATGACAAACAGAAAAAGGAAAAAAAACATGATTCTGAAAACCTTCAATCCTCACATGTTTCATGAAACAAATTTGACAAAATGGTGATACAGTGCCAAAATTCATACCATTTCCTCAACTGAGTAACTGCTCAAAATCAGATCTTGTCAAATGTCAAATTTAAATGTCTAGTCATTTTAGGGGAATATTGAAAAGTAGAGGAATAAAGGTTTTAAGGAAATAAGAGTGAGATTGTCTGCATTTTAGTATGGTTCATGAATCCTACCCCTAAGTGAGCAGCTACTCAGTTCAGGAAGAGGGAGAATACATTTTGAAGGGTATACCTTTTAGTAGGTCACACATCTGTGAGTATGTGCCCTACACAAATATGACTCCAAGCATAATTTTAAAATAAAAGATTGAGCAAGTAGAGGGAGAAGAAAGCAGCTAGAAAGAATTAGAGGGAGGAACAGGGATTTATATCATCAAAATTCATTGTCTATAAGTATGAAATTTTCAAAAATGTAATAAAATTATTATATGCATTAAAATGACTTTAAGCATACTCGGTGTGAATAAGTCAATATGTAAATGAGGGACTGAAAAGTAATGGAAAATAAAGATTCTGCATAGAAAGGTTTCACATAGACTTCTCTTCTGTTTACTGAAAACTCAAATATCCCACTTTACTTAAGTTTTATTTAAAACCAAAGTCGGTCCTAAGTACTCCAACATCAAACACAGTCTATTCTTGTCTGTGGGAAATGAAGATTGTCACCTGAGTTTGACATCTGACATTCTTCTCATGTTTGCTATGGCATGTGCATGCCTTCACTCACAGGAACATATACACATATGCACACACATTAGACTTGTAAAAATCGCACATTTTTCTGGAGAATTATTCATTTCCTTGTTCATTGGTTGGTTTTCTGGATGCAAGATCTCACTAAGTAGTCCTGGCTGTCCTGGCACTCACTATGTAGAACAGAATAGCCTTGATCTCATGGAGAACAACTGCCTCTTTTTCAAGAGTACCAGGATTAAAATGTATATATCTCCATGCCTGATTAAGGACATTGTATTTTCAAAATAAACTTGACATTCCTGTGGGAAATGCTCACAAGTGTAACTGTAAAAGCAGATAAATAGAATCATTATTTTTCTGTATCAACAGCATGTTTAAAGAGCTTATCCAAAAATTAGACCTCAGGAGGAGTGGAATTGCCATTTTCTAAAATGTTTAGATTCCTGCTTTACTTTTCAACTGTATGCTGAAATATCTTTTTTCACAGTATTAAAATATTTGTATTTGCAATTAGGTTTTTAAATACTGTCAAAAATTATGCCAGAAAACAAGAAAAATCCAACCTATTCTAGAAGGAAGAACAATAAATCTAAGACAGATGATTTCCTCTCCAAGGATTCCTCTTAGCAAACAGATGTGTAAGCAGAACCAGGGGACTCAAAGCAGACAACAACTGCACAGACAGGTTGGGAGTTTACAAAACCAAAACTTGCAGTGTTGAGGAAAGGGAAATGACACAAAGGAGCATATTGACTCGTGAATACCTGTTGTCTGAATTTTTCTTCCCTCTTTTCTGTTGTGACATTTACTACCCAGTAACACAGTGACCTAAACAACCAGTGCCATTGTAGGCTTGAGTCAGCTCTTTCAGGCTCATGTCCATCAAAGTTCCACACCTCTCCTCTAGTAGCTTAAGAGAAGCCATGGTGGTTGATAATTCCTACTGCCAGACAGATGGTTGTTAAGTGTATATTTTGAAGTCCTAAAAATTGTGTTGAATTTTATAGCATTATCCATCACTTTGTATTATTTGCTTATTTTTTTAAATTGAGAATTTCATGAATGTACATAATATGTTTCGATCAATTCCATCCCATTCCCTTTAATAGGAGAATAAGAGGTGCTGTTTGGTATCCTAAGGAGGGATGGCCTCACAGGAGTAAGTATGCCTGTGAGGAATGGGATTTGAGGTCTCAATGCCTTACATCATATCTGCTTTGCTCTGTTTACCACTTTTGGATCAAGATGTAAGCTCTCAGCTTCTGAATGAAGCCACTGCCATGATGAAGATAGAAACTCATACCTCTGAAACAGTGATCTCGAAGAAAAACTTCTTTCTGTAGGTTGCATTTTCTTCTTTAACAAATTTGCTTTTTTAGTGCTCCAATGACTAACAGAGATATCAATTAAAAGTGGGGGTGGGGGGAGACATACTTACTTGAATAATTTTTGCACTATAATGTAGGTTTTGATGATTTCTACATTATTTTATATTTCAGAACTATGTTCCTAAGATGTTAGACACCTCAGGGGATAATCATGACTGAATGTTATGACCCACTTAGTAGGAGGAAAGAGAACCAGTACCCACCAGCTATCTTCTGACCTCTACACACATGCCAGGGTATGAACATACCCAGTCCCATGTACAAAATATATGATAAAGTTTTAACACATATTATGCACAAGTAAACTAATAAGGACACTTACTACATGTATAACTGAGAACCAAAGTAATTGTAAAATCAAGTATTTTGTGATATTTCTCTTTCATGTGCAGACAGATACAGAAGTCAAAACTGTCTTTCTGGCTGTTTTAATATTACACATGGTTCTATGCAAAAAATACTGATACTATATTTTAAAAAGAAAAAACTGTATATGCATGCATTGTTGCATATTAAACACTATTAAAAAGATATGAGTCCAAACATTCTATTTCAAATTTCAGATTATTGGATTCTGACACATGGAAGGGTTACATTCCCCAAATGTCTCACCAGCACAGGTTCCCAAAAGCTACGAAAACCCAGGGCTAATGAGATGGCTCTTCATGTAGAGGTGGTTGCTGCCAAGCCTGGGAACCTGAGCCCAGACACAAAGATATACACACATGAACTCAAAATAAATAAGATAAGGAAGAAATTTAAATTTTAACTATATATAATTATGATTATCCAATAAGTTTTCTGAATGTTTCTTGGGTTATACATCATAATTATGATTGACAGGCTATTAAATTTGAGTGATATTTGTAAAGAAATAACAATAAATTATATCTATAGAAGTAAGACTTTATCTTTGGAAACAAGACTGTATGCTCTATGTTATCACAGTTGTTTAAATAGAAAAAGGCTAGAACCTCCTGTTCCACAAACAAATGTGAATTCTACTCAGGCCTGTTTACTGCAGTGTCTTTGGTTATGTGAACAATAACCAAACGTTTGTGCCAACGTTAGAGCATTGTTCACAAGTGCATTTAACCTCCCCAGTATTGCTTATGAATCCATGGTTCAAGTTCAAGAGTGTTGAAAACTTGATTGAAAATAGCCAGACTTGATATTCTTCCACCAATATCTATCTGATTGGAGGAATGGATGATAGACATCAGGTTTAAACATCTACCATTCCAGTTAAGATAATATGATAGCATCTTGTTCTTAGTCTTCCTTTTTCTTAATAGGGACATAAAACCAATGAATAAAAATATGCCTGAAACATGGGGCAGGCATTGGGCATTGAATTGACAATAAAAGTCAATTTTCCATCCCTAGTAGAGTTCTCCAGGAACCTATTTGTATACTAAATGACACAATGTCAACATCAGTGCAGAACTGTCAACTGGGATGCAGGACACTGCTCATGCCAACCATCCTGAAAGGCAGCTATAAAAAGCAGGGAGCTACTCTGAGCCTTGTCAGTGAGGTCCAGATATCTACAGAGCAGACACAGTTGCTCACACATGATGAGGGTCATCATCCTCCTGCTCACACTGGATGTGCTAGGCGTCTCCAGTGTGATGATGGAGAAGAGTCTCAAACAGAAGGTAGCAGGCCTTCAAGGAAGGGGGCTGTATGTGGTGGGCATGCTGGGTAGAGACAAGCAGAGAGACAGAGAGAGGCTTGGGAGGGGGATTTTTGCAGGTGGGGGTGGGCAGCAAGAGACAGAGAGGCAGAGGCAGAAAGACCTTAAAAGGGGGAAAGAATGTTCTGGAAGCTGGAATTTGCTAAGTCTGGTAATGGAAAGTGACAAATGTGTTGGGGGGGGAGAGTAGTTAACTCTCATAAATGGTTTTCCGAAAGAAAAAAAAAAAGAGGGACAGATACCAAGAGATGAAGACAGAGAGAAATGAGACAGTTCCTGGAGAATTTTGATAGGAGTGGATAATTACCCTCTGGAAGTTGCTATTCTCTATGTCAGGAGTGGAAAGACAGAGAAAGTGAGGGTGGTCTAAATGGTGGGTTTATGAGATAGAGTCACAAAGAGAGGGCAAGACACAAAGACAGAGATACACACAGGGAGAATAATGCTTCTGAAGATAGGTTTTCTGTGTATCTTTGTGTGACAGAGAGAAATATGAGTGGGGAGGAATGTTCTAGACATATTCTCTACATCTCAGAGTGGAGAATGGCAGGGAGAGAGAATAGTCTAAAGGGTGGTTTTCTGAGAAATAGAGACTGAGACTGAGAGACCAGAGAGAAGACACACACAGAGACCTGGATAGAGATATAGAACATCCATCAGTCTGGCTCCAAGGGTATGTCTGGCTTTCATAGCAAATCCAAACACTTACATCACTTCCTATGTTTCTACTCATGTGTCTCACCCTATGAAGAAATTGAAGCCATGATAATGAATATGATGAAGTAATTTAAAGGGGTGAATTGTACTAATGTTATTCTAGCAGTGACTATTGTCTACTTTGGCCTGTTATCCAAAACATTAAAAGTAAGGATTCAAAAAGATCTAAGGAAAAGCAAGATGACTTAATTCAAGGCAAAGCTAGAGTACAGATTACAGACAGACATACTGCCAAAGATGGACAGCTGCCCACTTGACTGACTGGACTCAAGATCCCCAAGCTATTATGTATGTCTCTATCAGGGGTCCCAAGGAGACCGATGAGCTGGTTACTAAGGGGCCAGATTTATTTTGATTGACAGATTAAAACATTTATTTCTCTAACTGTATATGTCCCTTCTCATAGTGCATCTGATTTTAATCATATATATGCCAAATTACACAAAGCCATAATAGTTAGGGTAAGATCACCTGAATAATGTTTTTGTCTTACTATGCATATACCCCAAATCACTTCAGGCCAGATCAGAGCTTCTTTTCTCCATATCAGGATACATTGAGAATATAGTTGAATGGGAATTGTCTTGCCTTGATCATTGTTAGGGGTGTGGGAAGCCCTACATCACTCTTCAGAGATTGGTATATGACTAGCCAGATGTGGGGTTGGGAACCATGAGGGATTCTTTTGTGTATTCTACAGCCTTATTCACAAATGAGTGTGTGAGTATTCTGATGCAGGTGGGGAAGAGGAAGAAGGGGTTGTTATACGGGGAATTCTACATTATTGTTTAGAAAGGAGTATGTGAGTAGCTTCATGCAGTTGGCTTGGAGCGGGGTCTAAAATCATTATTGGGTTCATTGTGTAGAGTATGCATGAACATAAAAGAAGCTAAGCTGCCTAATGCCTTATTACATAATCAGGTTGTAAATTACCCCATCCAAGTGGGTTTGGAATCCAGGGTTACTAAACTATCCTCTACTTTTGCCTGCCTTGTTTCCATAGCTGCTTTTTTTTTTTCAGATTGAAGGGAAATGGCGAACAATTTACTTAGCTGCCAGTTCCGAGGACAAGATAAAAGAAGGCTCTCCATTGAGGACTTATTTCCGTCGTCTTTTGTGTGGGAAGAAATGCAACCGAATCTAC

>JADRCF010011507.1 Grammomys dolichurus isolate MVZ Mamm 221001 194106, whole genome shotgun sequence

TAGCTGTACTTTTAATTTAGAATGAGATAGTTTATTTATTGTAGATATTTACTATATATCAGTATCTACCTATGTATGTACATATATACATGCATATATCCATCTATTATTTATCTATTTATGTATCTTCTGTCTCTTTATCATGTTATTTGTCTACCTACATGCCTATGCATACATCTATCATCTGTTCTAGCTAATTATCTATTATTTAACTACCTACATTTGTGTATATGTATCTTTTATCTTTTTAAATCTATTACCTATGTATCTATTACCTATATATAATCACCACCCATCTCTTGATATTTATTATTTTACTTTGGCATTCTATACAATAAATAAAAGAGTAACAGTTGAAAATCAAGTAGGTGTATACACTAGCATGTTGAAGAGATAAAATTTTTATTTTTAAATACATTTTTTAAACTAAAACATAATTACCTATTTCTCCCTTCCATTTTCTCCCTCCAGTCCTTCCCATGTACCCTCCTACCTTCCTTCCTTAGTTTCTTTCAAATCCTAGGCTTCTTTTTCTTTAATCGTGTGTGTGTGTGTGTGTGTGTGTGTCTGTGTGTCTGTGTGTGCTCTTAAATATGCAACCTGTTCGTTCTATATAATGTTACTTGTATAGGTATGATTTTACAGAAACCATTTGATATTCAAAAACCAATTGATAGGAAGGATTTTTCTTCAGGTAAGACTACAAAATATTCTAAAATTCATTTGGAGCCAGAAAAGATCTTATGGTAGCTAAAACAATCCTGAGCAAAAGGAACAATGCTGGAGGGGTCGTCATTCCAGACATGAGTGATTTTTTTTATATGCAGAGAATAGACCCCAAGGCTTTCAACAGGCTTTATGTAACCACACCCCTGGTCTAAACAGTTATTTCCATCTTTTTGTTTGTTTTTGATTGTGTGCCTTTAATGGCTGAGTCATCTCTCCAGCCCAGTTATTCCTTGTTTTTAGAGCCATAAAGACAAATCTAAGCTGCGACAGTCACTATTAGTCACATGGATCTCTGATGAGTGAATACAGATCTGAATACAAGACCATGCCTACGACTCTATAAATCTCCTGATCTGTCAATGTACATTGCAACTTAAATCTTCTGAGAATACAATTGTAGGCTGAATTTGAATCCATTTAAATGCACAAAGCAATTGCTGCTACATAATCTATAAGATTGGTGATATTTATGAGATGGGCAAAGAATAAAAACATGATTCCAATGTAAATATGTAGACTAGCCTATAGTATGTGTAGGGCACTGAGATGTGTTCTGCACCAGGTGGCCTTGCACCCTTTAAACCCAATTTCTACAGACATTTCTCATCTCAGATTGGGGTAGAATTTTTTCTCAATACTTTGGATTTTTTTTTTCATTTATCAATAGTCGTTATTGGGTACTGGGTATACAGCTGTGTTGGGAGAGTGATTTTACAGCATGAACCACAGAGTCCAGGGTTCCATCCTCAGCACAAAGCTGCAGTCCTACATTCCCATCATCCTAGCACTTGGTGAGTAGAACCAGGAAGATCAGGAGTTCAAAGACATTCATTACTCAGAGGTCAGGATAGGCAACAGATATCACACAGAGGAGCTAATTCTACCCTAACAAAGACACCTGCAAACACCTAACAGGGTGATTGATGCCATTTAAGAATGTATTAATTATATTTAGGGAAGATGTCCTACAACATCATATTCTTATGACAGATCTACAGAGGAAAATGTGATTTGCAATTAACTGTCCTGTCATTTATGAAGAATCAGGACTCTGGAGTAATACACAATACAACAACATACATGCATTTGTTTTTCAGGGAAGGAGCCAAGTGCCATGAGCGTAAAATCGTAGGAAGGAGAAAACAAGAAGTTTACTTTGCAGAGTGTGAGTAGACAATGCTGGGGTGAATGCATGACCTTAATTGTTTTCTTTTTTTCAAAATTCACGGTCATAGTTGAATTTTGTATATCTGATATCTACATTCCAATGCATTTGTCTCTACAAATTACAGATGCAATCTTGAAGAAAGCCTCTTCATTGTCTTCCTAGTCATTTTGGAGCTAATAAGAAATGTGGTTTGTTTGTTTGTTTAGTTTTTAATGAATTTTTAAACATTTTTATCTATATGGTGTATGTTTGTACCAATCACACTTCCCTTGTTTAGCTTTAAGCATAAACTAAATCACAGACAAGAGTCTGAGAATGGTTAAAGGAATTAAAAGGTAATTTGAGCACTTCAAAAAAATAACTTGATGCAAAATAGGAAATAGGATTTTATCAACTATTAAAATGACTCCCAGAAATTCTGAAACATGGGTCTTGTACATGTAATGATAAATTATCTAAATTTTATAATTTTATTGGGGAGAAAAATTATTTACAATATATAAATTTAAGCATCATGAATAGTCTTATCTCTTTAATTCTGACAAGCAAGGCATATAACCTAAAACACATTGAAATCCATTTATAAAGAATAGAGGTATCAGTTTAATTTGTCATGAAACAAGTGATAAAGATACTTTCATTATTTTCTT

>JADRCF010064522.1:c5644-1 Grammomys dolichurus isolate MVZ Mamm 221001 812437, whole genome shotgun sequence

TTTGTACTCTCAGGCTGGAGGTTTAGACTTTGGGAGCTCTGGTTGAATCTTTTGATACTCCTTCTAGAAAGGACCAAAACACCCACACTTTGGTCTTCCTTCTTCTTCTTGAGTTTTATGTGGTCTATACGTTGTGTCTTAGATATTGTGAGCTTTTGGGCTAATATCCACTTATCAGTGGGTGCATACCATGTATGTTCTTTTGAGGTTGGGTCACCTCACTCAAGGTGATATTTTCCAGCTCCATCCATTTGCCTAAGAATTTCATGAACTCATTGTTTTTAATAGCTGAGTAGTACTCCATTGTATAACTGTGCCACATTTTCTGTACCCATTCTTCTGTTGAAGGACATCTGGGTTCTTTCCAACTTCTGGCTATTATAAATAAGGCTTCTATGAACATAGTGGAGCATGTGTCCCTGTTGTATGTTGAAACATCTTTTGGGTATATTCCCAGGAGTGATATAGCTGGATCCTCAGGTAACACTATGTTCAATTTTCTGAGATACCGCCAGACCGCTTTCCATAGTGGTTGTACCAGTTTACAATCCCACCAACAGTGGAGGAGTGTTCCTCTTTCTCCACATCCTCGCCAACATCTGCTGTCACCTGTGTTTTTGATCTTAGCCATTCTGACTGGTGTAAGGTGGAATCTCAGTGTTGTTTTCATTTGCATTTCCCTGATGACTAGGGATGCTCAACACTTCAGCATTATTTTCTATTAAACCATAAAGTGTCTATTTAACCCAACACATTAAACTGATAGACATGTCATGGACTCTAGCTTGGTTTATAAATGAGTTTATTGTACCTAAATACAGAAGAATGAAGTCACCAATATGTGCAGAATCAAGCATATATCCCTCCAAAGAACCCTATACCCCACCTTGTGTGTTATGTAATTTAGTTTGAGGCCAGCCTGGTCTACAGAGCAAGTTTCAGGCCAGTCAAGGATATATAGTGAGACCAGTCTCAGAAAGAAAATGAGAAACCACTAACATGAAAATGAACAGTCATCATATCAATGTGGAGGGTCTTAGGTCAGCCCCTTCCCCTCTTTAAGAAGTCTTGTTAGTAAGTGTATTGGAGTAACTTACAAAATCATGTGTAAATATGGCTGCATTTCTGAAGAGCCCAAACCAGCTTGGCTGACACTCCTGAAACCTTTATACTTGGAGCTTTAGCAAGGCTTACAGACAACTAAGCTAGTCAGAGTCTTCCACAGGGCTTGTTCACTGTTTCCCATAACACTATAGAGTGGCCTGCTGAAAGTTGTTATTCTCAAAATCATCCTGAGACTTGTGGGGTTTTTTTTTGGGGGGGGGGAGTTGGTTTTTTGGAGACATGGTTTCTCTGTATAGTCCTGGCTGTCCTGGAACTCACTCTGTAGACCAGGCTGGCCTCGAACTCAGAAATCCACCTGCCTCTGCCTCCCAAGTGCTGGGATTAAAGGTGTGTGCCACCACCACCCGGCCATGGGTTTTTTACTTCTCAAATCTCATGTGCTACCAAGACAGAGTGTTCTAGTTTGGAGAAAATCGTTATGTGGCACCCTCTCTTTTTTCTTCTGTCATGTCTCTGAGGAAAAAATTGGTAAAGTTAGATAATTGCACCACTAAATGTAGATCAGATCCATAAAAATGTCGGTATATAGAATCAGAGAGCTTTCCTCAATGCGTGTATGTATGTGTAGATAATTCCACCATTAAATATAATAGGATCCATGAAAATGCCTATATATATATACACTCACATATATAATATATATACTATATATTACATCTTTCCTCAATATATGTATGTGTAAACAATTACAGCATTAAATTTAATAGGATCAATGAAATGTATATACATATGTGTGTTTACATATAATCATATAGCTATTCTCAGCAAGTAAGAGGATTGCAGTATGCCACCTTCTATGAACTCATATATCTTGGTATCAGGAACATAGCTATCCCAAATCAATATAGATTCTACATATTTGTAGTTAACTGTGGGATATTAGATATATTTTATATCTTAAAACAAAATATTAAACTTGCTTTATAATTCCCTGTTTGTCTTGACTAGCTTCAATACATGGGCCAGAATATTGTGAGTCAGAGATTATATACTCCATATGCACATTCATAACTGTGACATTAGAATGAACCTGAACAACCATATTGATGATTTTATATGTATTTATACAAATTTCTTATCTATCTCAGATGAAGGGAGCAACGCATTAGTGTTAAAGACAGTGAATGAGAAGATATTGCTGTTTCATTATTTTAACAAGAACAAAAGAAATGAGGTCACACGAGTGGCTGGAGTTTTGGGTGAGTGTCACACATGGAATTAGTCATCTGAGTATGTGGTTCAAGGACATGGATGCATGCATATCTCTGTACCCAAAGGTCACAATGTGTTTGTGCATCTGATTCTGTTTGTCATGTTGATAAATCACTGAAGCTGTAATGGTTAGCTGTGAAGATCAACTTGACATAACCAGAATGCCCTGGAAAGGGTCTCAGTAGGGAATTTCCCATAGCAGACTGACCTGTGGATGTATCTCTAATGATAGTTAGCATAAAAAGTCTTCACCCAATGTGAGTGGCACCATTCAGTAGCAAGAGGATATGCTGTAAGCTTCAACTACCAACAGACACAATTCAGAATCCTCTGACAAAGTTCTTAGGGAAGAATTTCTCTCATGAGACTGGCCTATGTGCGTGTCTCTGGGAGTAAACTGAATGATGACTACAAAGGGGGCTCTGCCCACTGTGTTCAGCACCATTTGCTGGGAAGATCATCCTGACCTGTATCAGACTTGGAAAGCTGGTTAAATGCTAGCACACGTATGTATGCATTCTTTGATCTCTGTTCCTCACTATGGACATGGTGTCTCTGCCCCCTAAACTTCTCCTGCTGGAATTTCCCCCAATTAAATGTGCTATAACTGGGAAAATACACACTTTTCTACCATAAACTGTTTTTGTCAAAGCATTTTGTCAGAATATTGAGCAAGGAAATTGAGATATTGATAAAATTGTCAATGTTACAAAACATGAGAGACAGTATATTACATCCTGGGGTTCACCCACTGCTTTGATATCTTAGGAAAATGATGCAAATTTGCACTGTAAAACTATTGGAGAAAGGGGGAAGATATTGTGCTTTGGTGCTTTTTAGGAGACTAAGCAAAAGAGAATATTTTATTAGTGTGCTGGGTACTAATGTTGTGACATCTTCCCTATACACCATGAAATGCAGGAAAAGGCAATCAACTGACTAAGGAAGAGATGACGGAGTACATGAACTTAGTGGAATCAGTAGGCATTGAGGATGAGAAAGTACTGCGTGTCATGGACACAGGTATAGTAGCAACCTGTGTGTGTAACTTCTTACTTTGCATTTTTATAATGAATATTCATTTTTGATTATAGAGGGTATTGGGAAGATGGCTCAGTGGAGAAAGTGCACTCATGCATGAGGTCCCAAGTTTGGATCCCAGCACCCATTTAGAAAGCCAAGAATAACAGAGCTGGGAAGGGGAGGGGACAAGAGGAGATTTCTAGTAATAGATCTATCTGAAGGATCATGTCTCAAAACTAACAATGTATAGAATAAATAAAGAAGATATTACAAAGTCAGCATCTTACCTATGCATGCATGGGTGTACCCATGTCATTCACATGACTATAGACATACCAATGAACCATACAGATAGATACGCACAAACAAAATCTACCTGAAATGAAACTCAGTTCCCAGGATCCTTTGTTCAGATACTCTATTCAAAACCTAGCTTTTTATCTGCATTTTTATGAAAACTGTTCATATGCAGTAATATTATTATTAATATTATGTTGAAGTGCAGTATTACTTTAAAATAATAGTGTGGTTTTACATTAATAAATCACTTTAAAATAATATTATTTTTCCATGTTTACTCTCCTTTAGACAACTGTCCAGACAAGATCAAGATTAGGTGAGTCAAAGCACGTTCATTTTATATCTTGACGTTTCATTTTAAATTCAATTTTTAAAATATTTTATTTGTTCTTTAACTTTTTCATATATGTATATAATATATCTTGATCTTACCTACTCCCACACACCTCATCCCAGAGACCCAGCAACACATCTCCCTCTATACTTCATGGTTTTTTAAATTTATCATCATCATCATAATCATTATTATCTCAATGGTTCCAAATGGTGCTATTTCTGAATAGGGTGTGTCAATTCCTGGAGGCATGAGCAATGTAATACACCAGGAACCACAGCCATAGGGAAAAAATGAACTGCAGGGAGCTGGTAGCACATGCATTTAATCTCAGCACTCAAGAGGCAGAAGCAACTGTATCTCTGAGTTCTAGATCAGCCTAGTCTATAGAGTGAGTTCCAGGGCAACAAGGGCTACACAGTGAAACCCTGTCTCATAAAACCAAAAAAAAAAATTAAAGACAAAAAAGGAAGGGAGGAAGGAAGGAAGGAAGGAAGGAAGGAAGGAAGGAAGGNNNNNNNNNNNNNNNNNNNNNNNNNNNNNNNNNNNNNNNNNNNNNNNNNNNNNNNNNNNNNNNNNNNNNNNNNNNNNNNNNNNNNNNNNNNNNNNNNNNNGGGAGGGAGGGAGGGAGGGAGAGAAAAAGAGACAGAGAGATACAGAGAGAGAGAGAGTCAGAGAGAGACAGAGAGAGACAGAGACAAAGACAGAGAGGCAGAAAGAGGAAAAAAGAAAAATACAGACCCTCCCTCTCCCAGAAACTATCAACTGGCAATAGCTCTTTGGTTAGTGGTGGATTGTCCTGAGTCCCACTCTACTCCATTCTAGAATGTTAACTGACTTGATCTGGCCAGAGTCTTGTGCAGAACACCACAGCTGCTGTAAGTTCATGATTTAACAGGTCTGTCATGTTCAGAAAACAGAACTTTTTGGCTCTACTCCCCATTGATGGGTGAGCATCCACAGTTACTTGGCCTCAGCCCTTTGACCAGCTATAAATTTCTGTACGAACCACTCCCCACTCTAAAAAGTTGAGCAGAAGCCAGACTTGGAGGCACACCCTTAATCCCAACACTTGGGAGGCAGAGGTAGATGAATCTCTGTATCAGGCCAGACTGATCTATATACTGAGCTCCAGAGCAGCCAGGTACATAGTCTCTAAAGAAACAAGCAAAAAGGTAAAAAGTTGATTTGACCAAAATTGAAAGCAACATAAATCTATGAGTGTTTTTAAGACAGTGGCTTGGAAACATGACACTTTATCACAACTGGTCTCCTCCAGAGGCTCATGAGCTCCATTGTCATGGACTTTTGACTAGAATTACAATAGGAACCCACCCCTACCCCTGTTCTTCCCTAGATGTGATATCCCCATGGAGCTAGTATCATATTTAATCAGAGAGTGGTTGGTTCCCCAGTACAGCGTTTATTGCACCAGTGGACACAAGGATGCTTTTATAGGGCTGGAGAGATGGCTCAGCGGTTAAGAGCACTGACTGCTCTTCCAGAGGTCCTGAGTTCAAATCCCAGCAACCACATGGTGGCTCACAACCATCCGTAATGAGATTGGATGCCCTCTTCTGGTGTGTCTGAAGACAGCTACAGTGTACTCATAAATAAAATAAATAAATAAATCTTTAAAAAAAAAAAAAA

>JADRCF010071464.1 Grammomys dolichurus isolate MVZ Mamm 221001 822590, whole genome shotgun sequence

TGTTAAACTCCAATGTAAAAGTACCTTGAAACAAAAATATATATAATTTTAGTTTAGATTCATAAAAATTATATCCTCAAGTAAACATGTGAGTCCTAAAAAATATTTTTTATTTATTTTATTTTATTTTTTGGTTTTTCGAGACAGGGTTTCTCTGTGTAGCCCTGGTTGTCCGGGAACTCACTCTGTAGACCAGGCTGGCCTTGAACTCAGAAATCCACCTGCCTCTGCCTCCCAAGTGCTGGGATTAAAGGCATGTGCCACCACTGCCCAGCGAGTCCTAAAAAATATTAATTTAGGTTGTGATATAAGATCATTGTCATATTAAAAATTTCCATATATGGAAAACTTCCAAAGTTCATGTATATTCCCAAACATACAAAATCCTGTAAAGTGTTTTTGCATGATACATCTTGTCATTGTTTGCCTCTTTAATGGCTTGTGTTTGTTTTATTTCCACTCTCATCAAGTATCATGTATTACTATCGTAAATATATAAAATAATTCTGTTCCAACATTACAGTTGACATCGGGACTTTTGCAGTATATTCTTCCTGAAACCTGAAATGTCAATATGAAGATGAAGCAATCTTTTCTCTCAGATCAAATCTTCCTACTTACTGCAAATTACAATTCCTGTCTCCATACTTTCTCTTTCATTCATTCTTCCCCATGTTCTAATCGGTGTTATTGCATCTTTGAATGTTTAAATAAATTTATTTCACTTGCATACGTGTTTTTGAAGAAAGGACGCTAAAGTGCAATGCACATAAAAATATATTTGACTTTTTTAAAAGGAGAGGAGTGTTGGAGGGGTGGCTCAAAGGTTAAGAGTTCTTGCTCCTCTTCCAGAGGACCTGAGATCAGTTCTTAACACCCACATCAGGCAGCTAACAGCTGCAGCTCCAGGGGACCTGACACCCTCTACTGGACTCCTTGAGCACTGCACACATATTACACGCACACACACAAATAAAAAGTCGAATGCAAGTATGGGTTAATAATATCAGATGATTTTTATTAATAATAATTTGAGGAACTTGGGAAAAGCCATGTCTGGTGGCTCATGATTGTAATTTCAGCACCAGGGAAGGAGGTAGAAACAGATGAATCCTCTGATGTCACCAGGAGCCAGCCTAAACTACCTGAAATATCGCAGGGCAGTGAAAGACCATCTAAAAAGCTAAGGCAGGGGACATGGAAGATGGCTCAGTGGTCAAGAGCACAAGCTTCTCTTCCAGAGGACCTGGGTTAGATTCCTAACACCCATATGGCAGCTCATAACTCTCTGTAACTCCAGTTCCAAGGGATCTGATATGTACGTGGACAAACATGCAAGCAAAACACCAATGCACGTAACATTTTAAAAAGTAATAATAGACCTTGTAANNNNNNNNNNNNNNNNNNNNNNNNNNNNNNNNNNNNNNNNNNNNNNNNNNNNNNNNNNNNNNNNNNNNNNNNNNNNNNNNNNNNNNNNNNNNNNNNNNNNCCTGAACCTTCACCCAGCTGATACCCGGTTCTGAAAGATATCTGTATTCCCCCTGAACACCCACACTCCAGTGTCATTCCCTTCCCCACATCCTGCCAAATGTGTTTATAACCCCTGTGTGAATGAATTTCGATTTGAGAAAAAAATAAATAAATATTTTTTAAAAAAAGTAATAATAATTCAAATAAGGCAGATGTACATGAAGGATGGCACCTGACATCATCCTGTGGCCTTCAACATCCACATGCACACATGTATGTCCACAACACAGGTGAAAAACTACACACACACACATACACACACCATACATCAAAGAGTCGCATCATTACTGCATGTTACAGACTTCAATATTGTAATTTTGACTTTAGAAAGGAGGATAAATTATTATATTGGTTGTTTAAAGTAAACATTAAAAGTTTTGTGTGCTGTTAGTTTTCAACTTAACACAAGCCATAGTCATTTGGGAAAGGAGACACTCAAGAAAATACCTCCACCAGGTTTAGCTGTGAGGCATTTCTTGATTTGTGGCCATTGATGTCAGGCAAGGTCATCCTCTGCCACATATGTATCTGTAGCCATGGATCCCTCTAGGTACACTCCTTGGTTGATGATCTAGTCTCTGGGAGAACTGGGTGGTCAGGCCAGCCTATGTTGTTCTTCCAATGGGGTTGCAATCCCCCTCCACCACTCCAGTCCTTCCGCAAGTTCCCCCATCAGGTTCCCTGAGCTCAGTCTGTGGTTGGGTCCGAGCATGTGCATTGCTCAGTTGCTGGCCTGATCTCCCAAGGAACCACCTCACTAGGTTCCTGTTAACAAGCACCTCTTGACCACGGCAACAGTGTTGGGTTTGGTGTCTACAGACATGATGAATCCCCAGGTGGGGAAGTCTCTGGTTGGCCCTTCCTTCAGTCTCTGTTCCATTTTTTATCCCTTTTCTTTTTTTGCACAGGAACATTTCTGGGTTAAAAAACTTTGAGATGAGTAGGGCCATGCCTATCTACTGAAGGTAGTCTCAACAGGTTCTATCTCCCCCTTCTCTGGGCATTTCAACTAAAGTCATCCCCATTGGGTCCCAAAAGCCTCATGTTTCCCTGGTATCTGGGACCCTCCAGTGGCTATTCCCAGTTCCTCATCCCTCACTGCTACATATTTTTATTCGATTTCCTGACCTTCTGTACCTCTCTGACATCTCCTCCAGTTCATGATACTGCCCTCCCTTATTTCCTCCCCCTCCTCTCTCCCTCCAATATCCTCCTCTAATAGAAGAGAAAGTGAGAAAGAGCCTCGAATACTTGGGTACAGGGGAAAACTTCCTAAACAGAACATCAGTGGTTTATGCTTTAAGATCAACAATTGACAAATAGGATCTCATAAAACTGAAAAGCTTCTGTAAGGCAAAGGACACTGTCAATAGGACAAAACAACAACCAACAAATTGGGAAAAGATCTTTACCAACCCTACATCTGATAGAGGGCTAATATCCAATATATACAAAGAACTCAAGAAGGTAGACTCCAGAGAACCAAATAACCCTATTAAATGTGGAGTACAGAGCTAAATAGAGAATTAACCAAGGAATCTCAAATGGCTGAGAAGCACTTAAAGAAATGTTCAACATCCTTAGTCACCAGAGAGAGGCAAATCAAAACGACCCTGAGATTCCACCTCACACCAATTAGAATGGCTAAGATCAAAAACTCAGGTGATAGTAGATGCTGGCGAGGATGTGGAGAAAGAGGAACACTCCTCCACTGTTGGTGGGATTTCAAGCTGATATAACCACTCTGGAAATCAATCTGATGGTTCCTCAGAAAATTGGAAATTGTTCTACCTGAGGACCCAGCTATACCACTCCTGAGCATACACCCAAAAGATCCTCCAACATATAATAAGGACACATGCTCCATTATATTCATACCAGCCTTATTTATAATAACCAGAAGCTGGAAAGAACCCAGATGTCCTTCAACAGAGGAATGGATACAGAAAATGTGGTACATTTACACTCAGCTATTAAAAACAACGAGTTCATGAAATTCTTAGGCAAATAGATGGAACTAGAAAATATCATCCTGAGTGAGGTAACCAAGACACAAAATAACACACGTGGTATGTACTTATTAATAAGTGAGTATTAACCCAAAAGCTCACAATGCCTATGATACAACCCACAGACAATATGGAGCACAGAAGGAAAGAAGACCAGATTGTGAATGCTTTAGTCCTGCACTGAGGGAAGATCAGCTTGCATTCTTCAAGAACCCAAGACTATCAACCCAAGGATAGCACCACCCAGAATGGGCTAGTTTCTCACCCATCAATCACTAATTTAAAAAATTCCCTACAGGCTTGCCTACAATCCAATCTGTGGAGGCACTTTCTTAACTGGGTTTCCTTTCTCTCATATGAATTCAGCTTGTGTCAAGATGACATAAAACTATGCAGCACAGACAGTATATAGATGTGTATGTATCTATCACATATTTATCATCTAAAAAATAATCTTCTATGTATTCATCATCTATCTATCGTCTATTATATATCTATCATCTATAGGTCTATCTCTATATAGATGTATGTATCTATCACATATCTATCCATCATCTATCAACACATCATGTTTATCCATCATTTATCTATGTATCATCTATGTATATATCCATTGTCTATCTATTCACCATCTATCTACCTATCTATCTATTAAAAAATTAACATGAGCAGGGCCATAGTAGCACACATTTTTAATCCCAGAACTTGGGAGGCAGATCTCTGAATTCAAGGCCAGCCTGATCTACAGAGTAAGCTCCAGGATAGCCAGGGCTACACAGGAAAACTCTGTTTAAAAACAACAAAAAATAATGTGAATAATGATCAGGGAAGCCACAACACAAATTCCATCATCTATGGACATCCACGTACATGCTTACATAAAAATGTATGTTTCTGTTGGAGAGATGGCTCAGTGGTTAAAAGCACTGACTGCTCTTCCAGAGGTCCTGAGTTCAATTCCCAGCAACCGCATGGTGGCTCACAACCATCTGTAATGAGATCTGGCGCCTTCTTCTGGCACGCAGGCATATATGCAGACAGAATACTGTAAATAATAAATAAATTAAAAAAGAAACTCAGAAATTCTTTTTTTCTCAAAGCAAAGTCAGGGATTAATTAGTGATAGTTAATACCTTGACTTCATGACCTTTTAATTTAATTTTAAAAATATTTACTGTGTGTATATGTATGCCCATGCCACAGAGACCAGAAGATCACTTTTGGGTGTTGGTCCTCTCCTTCTGCCACGTGGGTCCTTGTGATATAAATCAGGTCAAGATAAGTCACAATCCTCATTGCCCACTGAGTGAACTCATCATTCCTGCTTCACAAACTTCTTCAAATGTCTCAAAAGGAAAAAAGAAACCTCAAATTTTCATATTAGAGCAATAGTGTCCTGACACAAAACTGACACAAAATGTTAACAAAGATATCCTTAGAAAATTATAATAAATTTTATTTATTATTATTATTATTATTGTTTTTTATTTTCCATGCAAACTAGTGCTCAAAATTCTCAATTATATGCTAATAAATTTCAGTCAGCATAATATAAAAAAGTCTGTGTATCATGGCTTAATTTGGTGAAATATATCAATTAATGTGCATTGGCTAAACCATAGGGAGTTATTTTGATAAAAGTTTATTGATTGGGACCATGTTTTTGTTTAGAAGTTTCCAGAAAGCAAAGAACATTCTCTAGATTGAAAAAGAAAGCACATGTAAATGTAAGTTTTAAAAAATGTATCTGTTCTACTCAAGTGCAGAAAGTTTTTTCAACAGCCAGAATGGGGAATTTTCTTTTGAGAAAAATAAAAATATCCCTGGCACAGCAAATTCCAATTTCATGCTTACCAGGATGTAGCATGAACAGACATATTCAGCTATGACGTGAACAGTGGAGAACACCGGGAAGCCTCTGATGGTGTAAGAGAATAATAATAACACACATACACTCCAAAAGTATTTGAATCTGTTATGTGTGCAATACTTATTTGATAAGAATTGTTTTTTGAAAAAGAAAGAAAAAGAAAAAAAAGCTGGGCTGTGGTGGCGCATGCCTTTAATCCCAGCACTTGGAAGGCAGAGGCAGGTGGATTTCTGAGTTAGAGGCCAGCCTGGTCTACAGAATGAGCTCCAGGAAAGACAGGACTACACAGAGAAACCCTGTCTCGAAAACACACACACACACACACACACACACACACAAGAATTGTTTTTTGGTCTGTAAATTCACACTTTGGTGTTTACAGAGTGAAAACAGCACAAAAATATTTATTTTGTGTAATAAGTGACCCACGGGGTTATTTCCAAAGTAATGACCCTCCTTAAAAAATGAAAAAGTGGCATTTAATCAGGGGGCCTTTGCATATTCATATAGAAAAGTGATTTGAAGGCAGATACATTTCTGAAAATGCACATTCATGACCATGCTCAGATTAAAGTCCTTGATTGCAGAAACATTTTTTATTGAAAATATATTCTTTTTCATGCAATATAATCTGATTATGGTTTCAAAATTTATCAGACTTGCTTGTAATGATGAAATGCAAATATTAATTTATGTAATTTTACCAAAATGCAAATAAAAATCTACAAATACAGTTTTTTCAACTCATGAAATCTTTGAAAACTTTTGCTGGCAAATGTGCACAGAATTAAATAAAGCAAAGTAAATTATGAAGCAGTTTAGAAAGTGGTGTAAAATGATTTGACATCTTTAGAAAAAAATCCTCCAGGATCCTGTCTTGAGTTCCTGTCTTGACTTCCCTCAGTGATGGACTATGATTTGAAAGTGTAAGACAAACGAGCCCTGGGTTATAATTGTGTTCTACATCCAGCCCAAACTCATTGCTTCCTGATTCACCAAGATATCAGGAAAAATGTTACAATCTCTACCCAACACACATGCAAAAGTATAGTTTGCTTTCTATCGCTGTGACAAGTCCCTCTAACTAAAACATAATTTAGGAGAGGAAAGGGTTAACCCATCTTACACAGGCTACCATCTACCATTGGGGGAAGTTAGGACAGAATCTTGAATCAAAAACCATGTGTGCTATGTACTGGTTTGTTTTTCCACTCGTGCTTAACCAATTTCCTAATACAGCCCACAAAGCCTGACTAGGGAATGGTGAGCCCACATTGGGAGGAGCCCTCCACATCAATCATCAACCAAGACATTCCCCAACAGATAATCCCACAGATCAATGTAAGATGGACAATTCCTCAATTGAGACTTCCTTCCCAGATGATTCTAGATTATGTCAAGTTGACTATGAAAAAATAGAGACTCTTTTTCAGCTATCACACCTTTCCCACCATGGTGGGTCACACTTTCAAACTATGGACCAAAATAAAGTCTTCCCTTAATTGACTACTGTCAGGTTATTGAGTCAAAGTACCAAAAAAGATATTTATATGGGTCTCTAAATTGTCTCTCTAGTAATAACAACAAATGATATCAAGAACTGTCTAAACTATCAGTTGTAGTAAGAAATCAAAATGCTCTGGATTAAAAGTCTCTATGGTTAAAAAGCACTCAGTAAATATTTCCAGGACAACCGTTGTAATTACTGTTGAGTATCAACTTGACAGGATCTACGATCATCTATAACATAAACCCTTAGGTATGTCTGTGAGAAAGCTGTGGTACAACTGAGGTGAGAAATCCCTCCCTGAATGTGGGCACTACCATTTAACAGCATTTGATTATTTATATACTTTCCTGCTTGTATCAGAGGAATTGGGGGGCAGAAACAAAGGGGTAAATGACATGGGGAAGGAGGGGTGTCTTTGAAAATGGTGAAGCCTTTCCCCACTTGGATCCCACAGAACTTAACATCCATCAAAGTCTCCCCAATCAGTGACTTTTACATTCTTCTGTCATGAATTTGAAGAATATTATTTGAGGACCCCAGAGCGTGGATTGTTTGTCTTGAATTGGGTACTTGGGTGCCCAATAGGTGGGCACTTGAGAGGGAGAAAATGACATAATGGACTAGGCAAGGTCAGAATGTCAAATGATATCATTATGACCTGAAGGGATTTTGACAGGATTTATGGTACTTCTGAGGTAATTCATGGTAGTGAATGAAAAGGGATTAAAACTCAGGCTGACATCCAATTTCAGGTACAAGAGGATTGAATAGAACACCATCCCACTATTATTTTAACAAACCACCCACCATGACTGGCATAGAAAAGAAGCTGGCAAGTGAATTTCTGATACCACCCTCTGACTTGGTATGTTGCCCACATTGACCTCTAATTCCTAGAAAAGCAAAAAGTTAAGAAAACAAAACTAGCCTCTTATTTTCAAATAAAGATTGAATGACCTACTCCTAACATCAAGAAAATCCTCCCCATTCTAATATTTTGTGTGAGAATTTGAAAAGCATTTTTCCCTCCAGGTTTATTCATATTAAATCTTTGGCCGTAAATTTTTAGCCATTTTGCTAGTGGATTTAAGGAGCTTCATATGCTGAAACATTGAACTGTGAATGAATGAATATGAATATGCAGGAAACAGATTGTCTGACACATCTGAATCATTTCTCAGCCACTTAACAAGACCAAATTCATACTGAAAAACAAAGAAAGATTTTTGGATTATCTATATTAGGAAGATCGACAAAGAGATCCAGCAACCTACTCCCACTTACCCCCTTACACCACCACCACCCCAACCCTAACACTACCCCACCCCCATCCCCTTATCACTGTTACCACATCACCATGCCACCATTACTCCTACCCTCACCCCTGGTAACAAACCCATTGCCAGGGACCTGATATAAGGTTAAGGTAGCAGTCCTGGTGAAGCTGTGGTCCCACCCATACCTGACCACATCAGAGAGTCTATTATCCTGAAATGTAAACTTAAAAGCTATCAAACTCTCTCAGGGAGAAAAGTTTACCTTTACCTGGTCTAGGTTTCTGGATTTGTCTTCTCTCAGAATGATATCCCTGAGGAAATTTCAGACTTTTGAGGCCCATTATTAGGATATTCCAGAAACTAGAGGGAATGGTACAAAGTCCTTACCCCTACCCCCAAGGCTGGTTAAAGCATCTCTCTATGGAAATCCACCATGAGAGGTTTAACTCTGTACAGAGACTCATAGCAAAAAAAAAAAATTGTCTACCCTTAAATTGTTTCCATTTAAATATTTATTACAGTTTTCTCATTATTCTTTTAAAAATAAAACTGAGATTATGTAGAGGAAACACTACCTTAACCAATGTTTTAAGATTTATTGTATATGTTAGGTCTCAAAACTGACCTGGCTGTCAGGTTCTTTCATCATCTCTGAGTCCTTACCTGTTATGGGGTATGGCTGACTCCCTACCCAAAATTTCTCTAGTTGGCCTGCTCCAGCCATTTTTGTGATCCAGCCCCTTTTTGTCTACCCCTTTCCCCTCCTGAGCTCTTGGTCTCCTGGCTCTCCCCTCGCTCCTTTCCCTCTCCTCACATGCCCCTGCTCAGGTTCTTGTTGACTCTGGACTCTCCTAGATGTCCCTACCTCTGCCTATGTGCTCCTCGTATCTACAATAAACCTCCGCTACCATACCCAGGAGTAGTTACGTCCTCATTTTTTTTCATTCAATATAAGCATATAAAATTTATTTCAGTACCTAAAAAGATGATAGAGGTAAATGTGGCTTTGTGAATGCTTTTACATTTATATTTAGGAATATATGTAGGTACATATATGCATGCAATAACAATTCATTTTTAAAATGGGGCATAAATTTGAAAGAGAGCAAGAAGGGGTGTATGGAAGGAGGGAGGAAATGGAAGGGGAAATGACATAATTATATTATTATTATCTCAAAAAATAAAAAGGAGAATTTAAAAGTAATACAAATATTGGAGGTGGAGACACAGCTCAGAGGTGAAGAACCCTTGTTCAGTCCCCAGAACCCTGGTGGGCAGCACACAACCACCTGTAAATCCAGCACCAATAGATCCTACACCCTCTTTCAGACTTAACACACACCTGCAAAAAGAAATACATGGCCCACACATAAAAAAGAAATACAACTTCGAAAAAGTAGTCCCACATTACAAAAATTTTCAAACACATATTCTTCCTGGAATCACTATGTCACCTTTTATCTGGCTTTACTGAAGAACTTTCATCAATCCACTGATTAAATTTTATTAATGCCGTAAAATGAGTAAATGCCTTGATCTCTAGGAAGCAAAAAATAAAATGAAAAATCAAAGCATATGTTTGGGGGAAGGGCGGGAATGCAAGTACCAATTCAGAGGTCACGGGGCCGGGAAGATCATTGGTAATAAACGAAAGTGAATGAGTGGGTACCTGGCCTGGCTGTATCCATCCCACTGGTTTGCAAAACAAAACTGAATATTACCACAACCCATAATTACAACAACTCTTCACTTTTGGCATCAGTCTCACTGCAAGAAATCACATTGGGAATAAATCAATTCACTAAATGTGAATCCTTGAGGGGGATACTAGGACCCCAGTATCATTTATTTCCCCCCCTCTCTCTGTCTCTCTCACTCACACACACACACACACACACACACACACACACACACACACACACACAAACTTTCAGTCATTTATTTATTGCCAGAACCTCCTATAGTTCCCTGTGGCCTCCACTACATAGCTGAGCATGACCTTGAATTCCTGAATTCCTGACTACCTCCCCTGCCTATGCCTCTCATATAATAAGATGATGGATGAGACACACACACCCGACCTTGACATTTATTGTTGTTGTTCTTGTTTTTCTTGTTGTACAAAGTCTGACCTTTGTGCAAGTTACATGGTTTCTCCTTCAATAGTTAAATAGGAGTTTACTAATTCCAATTCTCTATCATTAATTCAATTACTTGGAAACTAATGCTAAAGAGCAAATGATAGGAATATATATTTTGTGCACTTGTCAACATTCACTTAACAAAAATGGAATTATTGCCCTGTGTAGTGTTAGCTTCATAGAGATCTTGGCATAGAAACAGGGATTTTAGAAAAACAAAAAGGTGGAATGGGTTAAAGTTTGACCTTCAAGTGTGGATTTCTGCCTCACTCTTATCTCAGCAGAGCAAACACCTGGCAGAACCTTCCAGAAGGAGTGATGGGCACAGCTACCTGAAAAGATTCTGTTTGCTTAGAAAGGGATTGTTCCTTTATTGTTCCCTGCCAGCGCAATACTGGAAGCCTTCACAGCAAATATTTGTACATTTTTATTGCTCCCCACAAAATCATCCTCTGGTCACTGCAGATCCTGCCATCATGCCTGCCAGATGGCAAAGATCATCTGTCATGTGACCTATAAAAGCTGTGAGAAATTATGGGGTGTTTGGGAACTTTCCAGAGCATCGGCCCACTCATAGTTACAAAATTCTTTGTGTCTCCCAGGGCCTGCTGGGTGGCTTCCTGGTTGGAGTTTTCCATAGTGGCACACAACTGCAAAATACATTTTGGTAGAATCACGTCATTGGTGTCAATTGATTTCCTAAGAAAAGTTAACAGTCACAATAAAATTGGGTACTTTCTAAATTGCTTATGTCCAAATAGTTTCCCATTGCAGTCTCAAAACATGTGAATATTACAACCTTTATTTCAAAAGATTAATATTAATTTTAATTATGTTTGTATCTATTACATATTGTATTATATACACACATATATATTACAAGCTTTATTTTTACACTTTTAGTTTGAATTCTGTATACGTGTGTATATGCAGTACATATTGTATTACACATATATACATTACAATTTTTATTTTAAAAATTGATTTCATTTTAATTGTGTGTGTGTGTGTGTGTGTGTGTGTGTGTGTGTGTGTGTGTGTGTNNNNNNNNNNTGTGTGTGTGTGTGTGTGTGTGTGTGTGTGTGTGTGTGTGTGTGTGTGTGGTGTGTGTATGGGGTGGGGCAGGGTGGGCATGTGTGAGTATAGTACCATGAGAGGCCAGTAGAGGGTGGGAGTCCCCAGGAGCAGGAGCAATGGGTGTTTGTGAACTCTCTGTGTAGGTGCTGGAAGCTGAACTTGGTTCCTATTGTTGGCCTTGCTTGTGGGGGATTACATTGCTGCTCTTTGCTGGAGGAAGTATATCAGTGGGGGTGGGCTATTTGAGATTCTAAAACATCCAGCACCTTCTGTTTGGTTGGAGATGGAAGCTCTCAGCTCCTTGTTGATGCCACCTTGCCAGCCTCTTCCTGTTGTACTTGTCCCTTATGTGTGCTTCCCTCATTGCCATGCCCATCCCTCACCACTATGCTTCCCTGCCCCAATGGACTCATTACCCTTTGGAAGTTAAATGCATAACTCAGAATCTTTCTCACATACATTGCTTTTGTTTGTGATATCTTGTCACAGCAACAGTAAACTCTAATACTTAACACTAAGCTACCTCTCCAGCCCCCAAAGATTTTTGTTTTGTTTTGTTTGAGTTTTTGTTTTTAGTATCATTTTTATTTATTTATGTGTATGGGTGTGTGCCTCTGTGAGTTTTTTCTCATTAATTAATTAATTTATTTATTCACTTTACATGCCCTCTCCTGGTCCACCCTTACATAGTCTCTCCCCTTCTCCCCCTCCCCTTCTCTTCTGAGAGAGTGGAGGCCCTCTAGGTATCCCCCCACCCTGGCACATCAAGTCTCTGCAAAGCTAGGTGCTTCCTCTCCCACTGAGGCAAGACAAGCAGCTCAGTTAAACAAACAGATTCCATGAAGTGTTGGGAGAAGGACTGAAGGCCCTGAAGAGGATAGGAACTCCACAGGAGGACCAACAGTCAACTAACCTGGACCCCTGGGAGTTCTCAGAGTCTGAACCACCAACCAAGGAACATACACAGGCTGAACCTAGGCCTCCCCACACATATGTAGCAGGTGTGCAACTCAGTCTTCATGTGGTTCCCCCAGTAGGAGCTGTCCCTAAAGATGTTGCCTGTTTGTGGAATCCGAGAGTTTATTTTGTGTTTTGTTTTGTTTTGTTTTGTTTTGTTTTGTTTTGTTTTGGTTTGGTTTGGTTTGGTTTGGTTTGGTTTGGTTTGGTTTGGTTTTTTAACCCTGGGAGTTCTTAAATGGAATAGTTCTCTGTGTTACTTCTATTTCATTCATGCTACCCACAGTCTTGCTTTTGAAATCACATGTATTTTTCCTATTTTCTTCTCTCTAAATTTGAATCAATTTCTGGTATCAGCATGTTTAAACACCAAGAAGAATAATGTGTCTATTTTATATTTTGTTGTATCTATGCACACGGAAACACACATAAGCTTATATGCTCAATTATTGGAACAAATGCTCTCCTTCTTTATCTACTCTCACTATGTGACCCTTTATTTGTTTCAGGCAAATGAAATTACAAACTGATGATTAAACTAATAAAACTGTAAAATTTACAGATGATGTCATTATTTGCATCATTTAATTCTCATGGAAAAGGTGCCCTTTTTCTTACTAATGCTACTTACAGGCAGATACTCTTAGCCAAAACTAATCCTGAAGTGAAATTGCCTGCAAACACAATCCACTGATGACTGATAAGATTCCTGGGAATTAGGAAACAAGCCACAAGGTTCTTACAGGGAACAATTGATTCTTCAGAGACTGCCTAAGAAAACACTGACCCATTTGCAGAGAAGTTATTGAGTTGGACAAAAGTTAAAAAAAAAAATACTCACACTGCATTTTAATACTAAATCTGCAAAAGAATATGCTAAGAAAAGTCACACACACACACACACACATACTCTTGCACAATCACAAACACACACAAATAAATGGATAAGCCCAGTCCTGAGGGCCTGGGATATCAGCCATAAACTCAGTAGGTTGGGTAACAAGGAAGCTAGGACTCTATGTTTAAAGATGAATTTCTTTCAAAATCCACAGGAAGACATCACAAGTTGCCTAGGTCATGTGCTATATCCTGGCCCTGGGGTTGGGGAGTATCCAAGAAAAATTTGGAGGCACAGGTTATGATTATCTTAGCATATCAAGCAGCAAACTCAGACACACCTTCCTCTTCTGTCTGCCAAGGGATGTTTATTTCAGGACATTTTTCTGGAAAGACAGGTACCAATGTATATTGACAGATGAGGCACACCAATGACAGTCAAAGGAAAAATTTCAATTTCTGCCATTTCACATCAGTAAATATAAATATTGATGAGTAAGTGTCCTTCCTTCAAGACATGCATTGAATTGGGTTTTCATCAAATGTTCAGGCAGCCGGAGGAAGCCAGTGGCATCAAAGTTGGCCACAAATGCATAAAAATGTATAAAAACTCAGTAACAGTTGCCCATGTCATGCATCTGATATTTTTTTTTAAAGATTTATTT
