# Supplementary material for: Comprehensive Analysis of Rodent-Specific Probasin Gene Reveals Its Evolutionary Origin in Pseudoautosomal Region and Provides Novel Insights into Rodent Phylogeny
Source: Biology (Basel). 2025 Feb 27;14(3):239. doi: 10.3390/biology14030239 (PMC11940140; doi:10.3390/biology14030239)
Supplement: Supplementary file 1 [file biology-14-00239-s001.zip › Suppl Data Files/gPBSN/gPBSN_Microtus fortis.docx]

>NW_026068168.1:49000-65000 Microtus fortis isolate MF-2015 unplaced genomic scaffold, M_Fortis_MF-2015_v1.1 scaffold_289, whole genome shotgun sequence

AGTAGAGGTGATTGCTGCCAGGTCTGGAAACCTGAGCCTAAACACCCAAGTTATACACACATGCACTCAAAACAAAGAAGAAAGTATAAAATTATTAACCACATATACCCTGACTATATAATAAGCTTTCTAGCTTTTTCATGATTGAAAGGCTTTTAAATTTGAGTGGTGGTATGTAAAAAAAAAAAAAGAACATTAAATTATGTCTACAGAAGTAAAACCTTATCCTGGGAATCAAGACTGTGTGCTCCATGTTTAAATAGACAAAGGCTAGAACCTCGAGTTCCACAAACAAATGTGAGTTCTCTTTCTGTTGGTTAAACGCCATGTCATTGGTCATGTCAACAATGTGATTTGAGCCTACATTAGTGGTGTATCTACAAGTGCATTTGAGCCTCTGCAGTATTACCAATATTTCATTCATGGTTCAAGTTCATGGGTGTTGAAAACGTGGTTGAAAAATGGTGAGACTCAGTATTCTTCCCCCAATATCTGATTGGCGAAAAGGATAATAGGGAATAGATTCTAAAATGTTGATTGGCACAACACATAAAAAGGTTCAGTTGAAAGAAATCCACACATAGGTTTACAAAAATATAGGAACTGGTTGTGCCCAGAAAATAAAGTCCAAGTAGAGATGAGAAACTGTATTTTTGTGTAACTATCAGCTACATGTAAGTTTTAAGTTTCAAAATCAAGTTTTAACAGTGCCCCTTCCAGTTAAAATAACATGATAGCATCTTGGGTTTTGTCTTCCATTTTCTTAATAGGCACATAAAGTCAATGAATAAAAATCTATCTGAAACATGGGACAGCAATCAAGCATCAGAAACTGATAATAAAAGTTAATTGTCCATCCCTAGTGAAGTTCTCCAGGAACCAATCTGCATAGTAAATGACCCAATGTCAACGCCAGCACACAACTGCCAACTAGGATGCTGGACACTGCTCCAGCCAATCATTCTGAAAGGCTGCTATAAAAGGCAAGAAGGTGACTCTGAGCCTTATCAATCAGTGTGCTCTGGACACCTGCAGGGCAGACACAGTCACTCACACACAATGAAGGTCATCATCCTCCTGCTTATGCTCAATCTGCTCGGTGTCTCCAGTGTGATGATGAATAAAAATCTCAAAAAGAAGGTAGTAGGCCTGCATGGGATGGGCTGTGGGGGGCGGATGTTCTTGTCAGAGACAGACACAGGCAGAGAGACAAGAAGACACAGAGAGAGGCATGGGGGACCGTGGGGCAAGACAGACAGAGAGAAAGAGGCAAAAAGACAAAGAGGGGAAAGTTGGTATTCTCTATGTCTGGAAGTGGAGAGTGACAGAGGAGTAGTCTAATGGCTGGTTTTCTGAAAGAAAAAAAAAACAGAGGGACAGAGACTAAGAGATAGAAACAGAAAGTAGAATGCTCTGGAAGTTAGCATTCTATACTTGTGAGAGGAGAGAGAGAGAGAGAGAGAGAGAGAGAGAGAGAGAGAGAGAGAGAGAGAGAGAATAGCCTTAAAGATGGTTTTTGGGGATAGAGACCTAGAGAAATAAAGAGGCTAAGAGATAAAGAGAGAAAGACACAGATTAATGCTTCTGAAGGTAAGTTTTCAGTGTGTGTTTGTGTGGAGAGAGATGGGAGAGAGGGAAGAATGTTTTGGAAGTAAGTATTCTTTATGTCTCTGTGGAGAGTGGCAGAGAGAGAATGGACTAAAGAGTGGTTTTTCAGAGAGATAGAGACAGAACCAAAAGAGACACTGAGAGAGACATGGAAAGAGAGACAAAGAGAGAATGTCCATCAGTCTTGCACCAAGGGTGAGTTCAACTTTCTACTAAAATCTGTACACTTTCATCTCTTTGACATTTCTACTCCTGTCCCTCTTCCCATGAACAAATGGAAACCATGATGGTGGATACAATAAAATAATTGAAAGAGGCTGAATTGTACTAATGTTTCTGTTTGCAGTGTCCACTATTTACTTTGGCCTGTTAGCCAAAATTTAAAAGTAGGACTCACACAGATCTGCAGAAAAATCAAGATGACTCAATTCAAGCAAGAGTTCAGATTACAGACAGACACATTGCCAAGATTGACAGTAGGCCACATGACTGACAGGACTCAAGGGCCCCAAGTTATTATGTGCATCTCATCCAGGGATCCCAAAGGCAAATAAGCTGGTTACTAAAGGTCCTTTTTTATTTTGATTAACAGATTAAATCATTTATTTCTCCAACTGCATGTGCCCTTTACCACAATGCATGACTTTAATCATACATATGCCCGATATTACACAGAAGCATTAAGTGACCAGTAGGGTAAAGTTATTTGAAAAGCATGTTTTTGTCTTATTGGACATACACTCAAAACCAGATCAGTGTGTCTATTCTTCCTACCTGAATACATTGAGAATATACTTGAATGAAAACTGTCCAGACTTGATCATTGCTAGGGATTTGGGAAATCCTACACCATCTTCAGAGAGAGCTGCATGAGTAGCCAAATGCAGGTGGAAAGGGAGTTATTAATGGGTTATTATGTGAAGATCACTACACAATTGTTCACAGATGGGTATGAGAGTAGCCTGACACAGATGAGTAGGGAAGAGAAGAGATTGATATATGGGGAACACTATACCATTATTCAGAGAGGGGTTTGTGAGTAGCCTGAAACAGGTGGGAGGGGATAAAATCATTATTAGGTTCATTGCTCAGAATATGTACAAACATAAAGAAGCTAAGCCACCTAATGCATTATTATAAGGCCAAGTCATGGTTTACCCGGTTCAAGCCGAGTCCCAGGTTACTAAACTATCCCCTACTCTCAACTGCCTTGTTTTCAAAACCACTTTTTTTTCCAGATTGAAGGGAATTGGCAAACTGTTTACTTAGCTTCTAGTTCTGTCGAGAAGATAAAAGAAGGCTCACCCTTGAGAACCTACTTTCGTCGCATTGAATGTGGGAAGAAATGCAAGCAAATCTACCTCTATTTTTATGTCAAGTAAGATATAAAACAAAATAGAACTATCCAGGTTGTGGGATGTCAGCAGAGAAACATGATGTTCTCAGTCAACATTCATGTCAGTGAACACCAACAACACCAAATAAGGATTCTTAAATCATTTTGCTCTGTATGAGCCAAAAAAATCACATATGATAGCCATACTTTTAATTTAGAATGAGTTTATTGAGTGTGTGACAAGCCAAGGTAAAATAATAAATATCAAAAGGTAGATGATAATTACATATAGATGGCATATAAATACATAGATAATAGATATATCAGTACATAGGTGATACATGCATATGGAAGTAGATAGACAGACAGATAGATAGCAGATATATAAGTGGATAGATAATGGATAGATAGGCAGATAGATAGATAGATAGATAGATAGATAGATAGATACATACATACATACATAAATACATACATACATACATACATAGGTAGATATTAATAATACATAAATAGATAATAGACCCATCTATCTGGGGTGATCAAAGAAAAACACATTAAGTTGTAAAATGCATGTGGGAATCCCTATGTTCAAAACCAGCTCCTAGGGGAAGTTAAACAGCATAACACAATATCAAAAAATATACATTACGATTGTTTAATTTTCTATTTATCAGTGCTCAAAAACACCGTTGGGGAGAAGGTAAATAATCCCAAATAATAATTTCAATGCTTTTAAACTATCTGGATATAAGTGAAATTATCCATTGAGGATATACGTCTATCTGTGATAGTGGAAGTTTTTATCAAATATAGTTTATATTTGAAACTTTTCATTCATTAATGATTTATTTATAATATCAGTTTTCTGATACAGAGTCTCTTTATATCATGCTGGCTTTGAACTCAACACAGAATAGAAAACAAACTTTTTAGTTTTTTATTTATTATGTATGTTGGTTTTTCAAGACAATATTTTTCTGACTATCCTGGAACTTGCTCTGTAGACCAGGCTGGCCTCAAGCTCAGAGATATGCCTGACCCTATCTCCTAATTGCTGAGATCAAAATTATGTACCACCACATGCAGCCTAATTTTTTAAGTTTCTTTTTTTTATTATTATTACTTTTGAGAATGGCATTGAACCTCCTCTCCTGCATCAGTGTCCCAAGTGTTAGGATGACAAGCAATACAAAAATACTTGGCTTGATATTTAAATTTAACACACCTGAAATAATTTAAAAGAGCAACAGTTCAAAATCAATTAGGTGAGCACACTAACATGTTGAAAGAGAGATAAAATTTTTATTTTTTAAATACTTTTTTAACTAAAACATACTTATTTCCCCCTCCCCTTTTCTCCATCCAAAGCTTCTTATATACTCTCCTCCCTTGGTTTCTCTCAAATCTTATGTTCCCTTTTCTTCAATTGTTCATATACACATATGGTCTTACATATACAAATATAACATGTTCATTTCATATACTGCTATTGTATAAATATGATCTCAGGGCAACCACTTGGTATTTAAAAACCAAGTGGTTTGGGGGGAGGGAGGGCTTTTCCTCAAGAAAGACTATAAGATATCCTAAAATTCATATGGAACCAGAAAAGACCCGAGCTAGCTAAAACAATACTGATCAAAAATAACAGTGCTGGAGGGATCACTATTCCAGATACAAATGATATTTCTTAAAGCACTGAAAATGGACCCCAAGACCTTCAACAGGCATTATGGAACTACATCCCCAGTCTAAACTGTTAGTTCTATCTGTTATCTTTTTGGTTGTGAGCCTAGCCTTTAATGGCTGAGCCATCTCTTCTGCCCAATTATTCCCATTTTTTAAAGATAAAGACAAATCTAAACTGTGGCTGTCAGCTATTCATCACATGGACCTCTGGTAAGTGAATGTAGATCCAAATACATGGCCATTCTTGTGACTCTACGGGTCTCCTAATAAGTCAATGGACATCCCAATCTAAATCTCAGAAAACAACTGAAGGCTGATTTTGAGGCTATTTAAATTCACAGGGCAATTGCTGCTACAGAATCTATAAGATCAGTGATATTTATGAAAGGAAAATTTTAAAATGTGATTACAGTGTAAATATGGAAATTGGACTATAGCGTATGGAGGATGCTGCATTGTACTCTGTACCAGGTGACCCTATTAATCCAATGTCTACAAACATTTCTCATTTCAAATTGGGGTGGAACCTTTGGTCACAACTTTGGTTTGTTTTTTTTTCTTTAATCAATAGTTGTTCTTTTGGTACTGGGGATTCAACTGTGGCCCAGCATGTACCACAGATCCCAGGATTCCATCCTCAGCACACAGGTGTGGCCCTATACGGCCAACGTCCCAGGGCTTGGTGAGTGGAGGTGAGATCAAGTTCCAAGGTCATTCACTGGTCAGGGGTCAGGACAGGTGACAAGTGTCAAATAGAGGAACTGATTCTGTACCGTCAAAGATATCTGCAAACCCCAAACAGGGTTATTGATGACATTTAAGTATGCTATTGATTATATTTAGAGATGATGTCATACAACAACGTGTGCTTATGACAGATCTACAGATGAAAATGTGGTTAGCATTAAACAGTCCTGTCATTCATGTAGAATCCAAAATCAGGACTCTGGAGCAATCCAAGATAAGACAACATCCATGCATTTGTTTTTCAGGAGAGGGACCAACTGCCAGCGGTATGAAATCATAGGAAAGAAAAGGCAAGAATTTTACCAGGCACAGTGTGAGTAGATGATTCCAGGGCAAACGTATGACCCTAATTTTTTTTAATTCACAATCTTAGTTGTATTTTGCATATCTGGATATCTACATTCCAGTGCAAACATCTCTACAAATTAAAAATGCAATCTTGGCGAAAGCCCCTTCAATTTCTTCTTGTCATTTTGGATCTAATAGCAGACTTTGTTTTGCTGTTATGGTTGGTGCTGTTTGGTTTTGGTTTTGTTCTTAGGTGACATTTTAAAACATTAAACTGTGTTTGTACCAGGCACACTTCCCTCCTGTAGCTTTAGGCCTAAACTAAATCACAGACATGAGTCTGAGAATGGTTAAAAGAATCAAAAAGTGATTTGAGCATTTAAAAAAATGATGCAAAATAAAAACTAGGATTTTCTCATTGATTAAAATGATTCCCAGAAATTTCAAAAATGCATTTCAAAAATGCATAATTATGAACTAACTAAATTTAATAATTTTATTGGAGATAAAAATTATTTAGAGTATATAAGTGTAATCTTCATGAATAGTCCTATCTTTTTAATTCAGGAGTATGACCTAAAATATGCAGAGATCCACTTATAAATAATGAATCAGTTTAATTTGTCCTAAAACAGGTGTTAAGAATCCTTTTATTATTTTGTATTCCACCATAAATAGTCGACTTAGCCCAATGCAATAAAACAGACAGACCACACGTGACACCGACTCTAGCTCAGTTTATCAGTGAGTTTATTGGACTTAAATACAGGAGCAGACTCAACTCACATGCAGATACCTCACGAAAAATCTCACCCCCTTTTGTGTATTATGTAATTTCAGGAGACATTCTGTGAGTTTGAGGCCAGCCCGGTCTACATAGCAAGTTTCAAACCAACCAGGGTCTCGGAAGGAAAATGATTTACCACTGACATGAGAAGGAACAGTCATCATATCACTCGGGAGGGTCTTATGTCTGTCCCTTCCCCTCTTTAACAAGAAATGTTAGTAAACCGGTGGGTGTGCTGAAGTCACTTACAAGAGTATGTGTGACTCAAATATGGCTGCATTCCTAAGGAGCCCACCCCTGAAGAGTGGGTGACTCTCCTAGAAGCTGCATCTCTGGAGCTGTGTGTAAAGCTAACAGGCAGCTGGGCTAGTCAAAGACTCCTCTGTCAACAGCTGTTTGCTGTTTTCCATAACTTTGTGGAGTGGCCTACTGAAAGTTGTTCTTTTCAAAAAAACTTCCTGAGTTTCATGGGTTATTTACTTCTTGAATCTCATGAGCTCCCAATACAGAATGTTCTGGTTTGGAGGAAATTGCTGTGGCATCTCTCTTGTTTCTCTGTCATGTGTCTAAAAAATAGGTAAAGTTAAATAACTGTACCACTAAATATAATAGGATCCATGAAAATGCATATATATGTGTGTGTGTGTTACTATATATTATATAATATATATTATATAATCATACAGCTTTTCTCAATATGTATATATGTAGATTATTCTACAATTAAATTTAATAGGATTCATGAAAAAGCATGCACATGTATGTACATGCATATAATCATACAGATATCCTCAGAAAGTAAAAGGATTGCAGTAATGATACCTTCTGTGCACCTACACATCCTGGTATCAGGAATATTGCTATCCCAAATCAATATAGGTTCTACATATTTCTAGGTAACTATGAGATACGAGATATGTTTCATATCTTTTTTTTTTTTTTTTTTTTTGAATTTTCGAGACAGGGTTTCTCCGTAGCTTTTTTGGTTCCTGTCCTGGAACTAGCTCTTGTAGACCAGGCTGGCCTCGAACTCACAGAGATCTGCCTGCCTCTGCCTCCCGAGTGCTGGAATTAAAGGCGTGCGCCACCACCGCCCGTCTTATGTTTCATATCTTAAAGCAGAAGTATAAATTTGCTTTCTTAATTCCCTGTCTGTCTTGACTGGTTGCAACACATGGAGCAGAATATTATGGGCCAGAGGCCTATAATCCATGTATACATTGATAAAGATGCATTGTGAATGAGCCTGGACATTCATCTTGTAATCATTATATTATTTATATGTATATTTCTTATCTATCTCAGATGAAGGAAAAACAACATTCATGGTAAAGATGGTGAATGAGAAGATATTGCTGTTTCATTATTTTAACAAGGACCTTACAGGCAAAGTCACACGAGTAGCTGGAATTCTGGGTGAGTTTCACACATGGAACTCATCATCCAAGTATGTTGTTCAAGGACTTGGGTGAGTGCACATTGCTGTGCACAAAGTTCAAAGTATGTTTGTACATCTGACTCTGCTCTTCATGTTGATAAACCGCTGAAATGGTGATGGACAGCTTTAACGAAAAAACTTAGCACAACCCAAAATCCCCTGGAAAGGGTCTGAGTGGGTGATTTCCCACAGCTGACTGGCCTATGGGGGTGCCTTTGTTGATTATTGACATAAGAAGGCTCCACCCACTGTGGACAGCACCATATCCTGTCAGGGAGATGATGTTCTGTGAGCTTCAACTATCAACTGGCACAACCCAGAATCCTCCAGAAAGGGTCTTAGTGAGAGATTTCTTCATCAGACTAGCCTGTGGGCGTGTCTGTGTGAGTGTCTTGAATAATGATTGACACAGCAGGGTTTTTCTCACTGTGGTCAGCATCATTTCCTAGACAGACCATCCTAAAATACATCAGGCTAGAAAAATCTGGACAAACACAAGCACATATTCTTGCATTATTTGCTCTCTGTTTCTGACTATGGATGTAGTGTGTCAGCTCCCCCAAATTTCTCCCACTGGAATGGGCTATACCCAGGAAGTTTACTGCAGAAATAAACCCTTCTCTGCCGTAAATTATTTTTGTAAAAGCATTTGGTGAGAATATTGGACAATGAAATTGAAATATTGAAAGTTTATCTGTTAAAAAATAAAGACATGTTTCTTCTGTTGAGACATAGAAGATTTTAATAAAATTTCCACACATTTTTCCAGCATTTCTGAATTACACATCAACCAATCATCATGCCAAGTCTCATGTCAGTGTAGTCTGGGATTTCCATTTTCTAACAAAGCAGGAATTGAAATATTGCTTGACCACATTAAGTGGGTTTGTTGTTTGAATTCATGTCAACTGAAAATCCTGGCTTTCAGCATTATTGTTTCTACTTTACAAATCCTGACTCGCAAATCTTAATTATCACGTAGCCTGAAATCTGATTGTACTGGATTCTGTCAGTTTCTCATAGATGCAAACCCCCCTGAGCTGACAAATTCCTAACCCTGCCATCAAAGGTACTGGAGTATATTGTATAAGACCCTCATTTCCTACCAGAATTGGACCTTTATTTCTTAAGTTAATATGAAGGGATTATATTTCATGGGGGCATGTTACTCATCATCAAAGGTTTATTTTTTATGCGTCCAAGTGTCTTACCCACGTTTAGGTGTACCATGTGCATTCTTGGTGCATATGATAGCCATGAGATGGCATCAGATCTGGTCGTGCAACACTGTGTGTGTGCTGGGAACTGAATGTAGGTTCTTACCCATTCTTAACCACTGAGTCCAATGTCACCCCTCTAATATTTATGATGATTTCAGATCAGTTGGTTCAGCAATCTGAGGAAGCATCAAGAACCCATAAGAATGGATTCTAGAATATAAATGGAAACAATATAATGACATATAGTTATGATAGTAAAAATGCTACTGCTCAATCATTGTTTTCTGGAATTGCTTTGGAGGCTGTGTAGGAGTTTGGTATATTGAAGTCTACACCCCCTCAAAAAAAACTGTCGATATTACAAGAACAAGAGAACATGGCATGTTACATCCTGGGATCCACACAATATTTTCATTTCTCAGAAGGACATAGTTTCTTGTTATCAATCTAATGGAAGAAATGTGAGTAATATTGTGCTTTGGCTCCTCCAGGAGACTCAGCATTGAAAAGTATTTTATTAATGTGCTGGAGCTAATGTTGTGCCATCTTCTTCCTTATACACCATGAAATGCAGCAAAAGCCAAACAACTGACTAAGGATGAGATGACACAGTATATGGACTTTGTGGAGGAAATTGGCATTGAGGATGAGAATATACAACGTGTCCTGGACACAGGTAAAATAGCAACCTGAGTGTGTGTGACTTTTCATGTTACATTTTATAATAAATGTTATTTTTTTAATTATCTGGGGTATTGGAAAATGGCTCTGGGGGAAAGTGCACACAAGCATGAGTACCTGAGTTTGGATCCCATGTAGAAAGCCAAGAACACCAGAGCTAGGAAGGGGTGAGGACAGAAGGAGTCCTGGAGCTTTCTGGCAACAAATATAAGTGAAAGATCTTGTTTTAAAACTAAAAATATGTAGGATGAATAAGGAAGATGTTTAAAAGTCAGCCTCTTGCCTATATATGCATGGACTGCACACAGGTGAACTCACATGACCATGGACACACCAATGAACCATGAACATAAAAAAATCTATAAGAGGGGGCTGGAGAGATGGCTTAGAGGTTAAGAGCACTAACTGCTCTTCCAGAGGTCCTGAGTTCAATCACATGGTGGCTCACAACCATCTGTAATGAGATCTGGTACCCTCTTCTGGCCTGCAGGCATACATGGAGGCCAAATGTTGTATACATAATAAATAAATCTTTAAAAAAAATCTATATGTAATGAAACTAGACTCCTACTATCCTCTGTTCAGATATTCTACTCAAAACCTCGCTTTTCTTTCTGCACTTTCATAAAAATAGCACATATATAGTAATGATATTGTTAATATTATGTTAAAATAATGTATTGCTTTAAATATTATTATATTAAAATAATACATTACTTTGATAATAATATTATTATAGCAAAAATAATATTATTTTTCCATTTTCACTTTCTTGCAGACACCTGTCCAAAGAAGATCAGGATTAGGTGAGTGTTTAGCTCATTTTATATCTTGATGTCTAATTCATTTCTGTTTTTTAATAATACTTTATTTGTTGTTTAACTTTTCATATATGTATACATTTTGATCCTATCCACTCCCCCACACACACACACAATCCCTCCAACTATCCTCAAGACCCACCAATATATGTTCTTCTATACTTTATGGCTTTTAAAATTATTACTATTATTATCATTATCATTATTATTATTCGCTATATAAGTCCAATTAGTGCTATTTATGAATGGGGATGAGGCCATCCCAGGAGGCATGGGCAGTGTAACGTGCCAGGGGTCACACCTTTAGAGAAAAAAAAGATCCTTCCTCCCCTGAAGCCATCAAAGCCTCTAGTTAGGGGTAGATACTCCCAAACCCCTCCCCACTCCACTCCATTCTAGACTGTTGACTGGCTTTACCTTACCTGGGCCTTCTGCAGATGATCATGGTTGCTGTGAGCTCTCATGATTGTAACAGCCATGTCGTGTTCAAAAAAACAGAATTTCACAGCTCTCCTTCCAACCCGGGTGACCATTGATAGCTGAGCATCCACTGTCACTTATCCTCAGCACTTTGACCACTCCTCATTGTAAAAAGTTGATGTGAGGCCAGTTTTGGAGTCATACACCTTTAATTCCAATACTTGGGAGATGAATCTCTGTGAGTTTGAGGCTAGCCTGTTCTATATAGTGAGCTCCAAGACAGCTAGAGTCTCTGTCTAAAAATAAAAAATAAAAACAAAGTAAAAAATGGACTTGACCAAAGTTTAAGGCGGCATAAATCTATGAGTATTTTTTAAGATGATAGCTTGGAAACACGACAGTTTATCATCACTGATCTTCCCCTTAGGCTCCATGATCTCCATTGTCTTGGGTTTTTGACAAGAATTACAATAGAAACCCACACCTACTCCTATTCTTCTCTAGACATGAGAGCTCTCCCATGGAGTTGGCATCAAATCCAATCAGAAAGTGGTTGATTCCCCTAGGATGTCCTTTATTGCACCAGTGGGCATAAAGCTGCTTTTATAGCATGCAGGGTCATCATGGTGACATCATCAATGTCTTACTGCCATATACACACCCTACAGAGTGCCTCCAAGTACTGTAGAAACCAGCCAGCAGGGAGAGAATTTCCTAGTTTATTTGAGATTGATTTCTTGATGCCCTACAACCACAGCATGTAGTGTCTTCAGCAATGGTGTCTTACCATTTAGTTATGGTAAACAACCAAGAGATACAGCAATAATCAATTTGTTTTGGTGACTCCAGGACCTCCCTTTACTTATAAATGATAAGGTGGTACCCCATAACTGGAAATGAGATTTTCATTGAAAAATCCACATCGTCTGGAAACAGTACTGTGTCTTTGCAGGGTTCCTCTAATGAAACTCTCTTGTTTTATGCTACATTTTAATTAGCTTACAAACTAGTCGATTTTTGTAGTTTTGGTTGACCCACCCTACCTCTGTTCTTACCTATGGCCCAACCACATCTACATCTGCTCCTTTAACCCACAGCATGCCACCTCTAATCTTGTCTGTCACCTTTGTTCCATTTTCCATTTTAATTATACCTTACACTTTTGTTACAAGGTCATAGGTTTCCATATGATTTTTTTCCTTTTTTCCCCATATAAATTTTTAATAACCCTTCATTTTAATTAAACCTTCCATCATGCCCTGATTTTGTCGTTTTACATTCTACTCTGTGATTGAACCTTCCCGCACCAGTATTCTTCTTTATACTTTCATTTTAGTTGCATTTAACTATCTCTCCTCCCTTGACGGGTCTGTCCCTAATGACCTGTTTCTAATTACCTGGCTTCCATATATACTCCAAATTAAACATACAAAATAAAAAGTACAATTCTAAAATCAACAAGTTAAATAACATGCACAGTTTGTCTTTCTGAGCCTGGTTTATCTCCTTGAACATATTTTCCAGCTTCTTCTATTTACTTAAAAATTTCATTTTTCTCTATGGCTGAGTAATATTCCATTTTATATACTACAACACTTTCCTTATCCATTCATCAGTTGATGGACATCTAGATTAATTTCTAGCTATTGTGAACCTTACTGCAATGAGCATGGATATTCAAGAGTCTCTGTAAGAAATATTAAAGTCCTTTGGGCACGTATCTAGAAATGGTATAGCTGGGTCATATGGAAATTCTGTTTCAGGTTTTTGAATTTTTTGTTTGTTTGAGGTTCACGGTGTTTTATTTGTTTGTTTTAAGTTCTTTGTATATTGGAGACATTAATCCTCCATTAGATGTGTAGCTGGAAAAGATCTACATTACCTAAAATGACTCTGTATTTAATTGACAGCTTGCTTTGCTATAGTTTTTAATTCCACAATATTTGACTATTTCTGTTCTTACTTTGCCACAAAATCCTATTCAGTGTAACATGAGGGCCTGGTGCTTGTTGGGGTTTCTGTACCGCCCCGTCCCCCAGTCATTTAGTCCCAGAGAAAATCACACAGAGGTCTCCTTAAGATTATAAACTGATTGGCCCATTGGCTCAGGTTTCTTATTAGCTCTGGTAACTTATATTAACCCATTAACCTTATCTATGTTAGCCACATGGCTCAGCACCTTTTTCAGTGGGGCAGGTCACATCCTGCTTCTTTGGTGATTTGGGCAGGAGTGGCGGAAGAGCTTCCTTCTTCCCAGAATACTCCTGTTCTCTTTGTTCTACCTCTACTTCCTGTCTGGTTTTCCCACCTATACTTCCTGCCTGGCCAATCAACATTTATTAAAATATAATTGACAGAATACAGACAATTATCCCACGCCAATTCAGAAAGTTCACATCTGTGCCTATGATCAAAGTACACTTCTTGCTTTCTCCTCTATCAGTTTCAGACTACCAGGTCTTACATCACAGTTCTTGATCCATTTGGAGTTGAGGTTTTTGCAGGATGAGAGGGAAGGCTCCAGGGCCATTTCTCCACATCTTAAGATCCACTTCTCCCAGTTTGTTTATTCTTTAGGAATTCTATCTCTTTATTTAGTTATATATACTTAGTTTAGGTCATTTTCCTTCTTTCATTGGAGACATTGACAATATCCAGAAGGTGACTAATTCTTACTAGTGTTGAAATGGAATTCTCGTCTATGGGCCCAGTGTTGGGGAATGCAAGTTCTACCTCTAGTCTTCCTCCGAGAGTCAGTCTGGGACTTTGAGTGAGATTCCTGCTGTAGGGTACATGGGGTAAACCTCAATGTTAAAGAACCTTGAAACAAGCAATTGTATTAGTTTAGATTCATAGAAACTATGGACTCAAATAAGTACATAACTATATAAGTTCTAAGAAATCCCAATTTAGGTCTTGATGTAAGATCATTTATCATATTAAACTTTTCCATATATAGGAAATTTCAATTAAAAATTCATGTATATTATCAAACATACAAAGTCCTGTAAAATGTTTTTGCATGATACATCTTGTCTCTTTAATATCTTGTATTTCTTGTATTTTCCACTCTCATCAAATATCATGCATTACTATCTTAAATATATTAAATAATTATGTTCCAACCTTACAGATGACAGCATCAGGAATTTTCCAGTAAATCCTTCCTGAAGCCTGAAACATCAATATGAAGATGGAGCTTCTTTTCTCTTAGAAGATCACATCTCCCTATTTACTGTATATTACAATTCCTGTCTCCATATTTTCTCTTTCATCTGTCCTTTCCTGTGTTCTAATCAGGGTTAGTGCATCTTTGAATATTTAAATAAATTTTTTTCACTTGCATACACGTCTTTGAAGAAAGAAAGCTAAAGCACAATGCACATAAGTATCTATTGGACTTTTTTAAAAGGAGAGGAGGGTTGGATAGATTGATTGAAGGTTAAGAGTGCTTGCTGCTCTTCCCGAGGAAATGTGTTCTATTATTAGTGCCCACATTGAGCAGCTCACAACTGTAGTTTCGGGGAACCTGGTACCCACTTCTGGAGTCCTTGAGCACTGCACACAGATTACAGAGAGAGAGAGAGAGAGAGAGAGAGAGAGAGAGAGAGAGAGAGAGAGAACATAAATCTTATTCAAGTATGGATTAATAATAGCAGATAATTTTGTATTAAGAACAATGTCAGGGATTAGGGTGTAGCTCATTTGTTAAAGTGTCTGCCATGTAAGGGTAAAGCCCTGTGTTCAATTCTTAGAATCCATGTGAAAAGCTGTATTGTGGGCCATGTCTGTAAATTCAGCACCAAGGAGGGAGGTTGAAACAGATGAATTCTCTGATGTCACCAGGAGTGAGCCTAGACTACTTAAAAAATCACAGGCCAGTGACAAAAAAATAAGGTGGATGTGCATTAAAGAATGAAAGATGACACCTGACTTTGTTCTTTGTCCATCGATGTTCAGATGCACACACGTACATCCATAAGCACAGGTGTTCAACTATACACACACACACACAAGAATTTCATCATTCCTGCATGTTAAAGACTCTGCTACTATTGTTTTGACTTTTAGAAAGGAAGATAAATTATCATATTAGCTGCTAAAGGTGAACATTAAAAGGTTTGTTTGCTGTTAGTTTTATATCAATTTGGCACAAGCTATAGTCACTTCGGAAGGGAGATACTCAATTGAGAAAATGTTTCCACATGGTTGGACTGTTGAAAACGTTCTGGGATATTTTTTTGATAAATGGTTGATATGGGAGGGCCCAGCTCACTGTGGAAAACACTGCTGAGCCAGCCAGTAAGCAGCACTCCTACATGGCCTCTGAATCAGATCCTGCTTCCAAGTTCCTGCCTTGACTTTCCAAGGTGATGGGCAACAAGCTGTAAGATAAAAATAAACCCATTCTTCCCCAACTTGCTTTTGATCTTGGTGATGTTGGAGATCTTAGATTAAAATACATTTTTCTTCTTTTCCTTCCTCCTTTCTTTCTTTCTTTCTTTTTTTCTTTCTTTCTTTCTTTCTTTCTTTTTTGTTTGTTTGTTTTTCACGACAGGGTTTCTCTGTGTAGCCCTGGCGTCCTGTAGTTTGCTCTGTAGATCAGGCTGGCTTTGAACTCAAAGATCCACCTGCTTCTGCTTCCTGGGTTCTGGGATTAAAAGCATGCATCACCACCACCCAGCCCCAAATAATTTCTTAAAAGAAAGTTGAAACCAAATGCTCAAATACCCAACTATATGGAAAATGTAACCTAGTACAGAACACCATAATCATCTGATTTTTATTTTTCTATTGAATACACCATGCTCACATCACTTCTCTCTTGGTATCTTCATTTCCAGGAATTTGTCAAAAATATATCTTTCCAAAATCTTTTACTGTCCTTCTTAATTACTGTATTTACTCATGTGGAACCATTCATCAAATACCTAAAATAAACGCATATATGTACTTGTTCATA
