# Supplementary material for: Comprehensive Analysis of Rodent-Specific Probasin Gene Reveals Its Evolutionary Origin in Pseudoautosomal Region and Provides Novel Insights into Rodent Phylogeny
Source: Biology (Basel). 2025 Feb 27;14(3):239. doi: 10.3390/biology14030239 (PMC11940140; doi:10.3390/biology14030239)
Supplement: Supplementary file 1 [file biology-14-00239-s001.zip › Suppl Data Files/gPBSN/gPBSN_Microtus richardsoni.docx]

>JAGDQN020000290.1:22500000-22518000 Microtus richardsoni voucher IRGN JQ59JK4JDI isolate JQ59JK4JDI 290, whole genome shotgun sequence

CCCTATCCCTTTTAATTCTTGATCTCCAGTAGTTGATGCAGCTTGGGAAGACTAAAGAGGAGTGGCCTTGCTCGAGGAAATATGTCACTGGGTGAAGGGATTTGAGGTTTCAAAGTCTTGCATCATTTTTATTTGCTCTGTGTTTTTCACTTATGCACCAAGATATGAACCCTCAGTTTTTGGCTCCAGCTACTGCCATGATGGTGATGGAACCTCATACCTCTGACACAGTGATCTCCAATAAACTTTCCTTCTGTAGGTTGCTCTTTCTATTTTCACAACTTTATTTTTGTAGTGCTCCAATGAGCACCAGAGACATCAAAATTAAAAAAGGAAAAGACATATTGACTTGAATAATTTTTGCACTGTAGTATACATCTTGATGATTTCCAAATTGTATTTAAGAACTTTATTCTTGAGATGTTATGCAGCTAGGTAATGATAACTAAGTACAATCTTTATGACCCACTTAGTAGAAGGTGAGAATCAGTTCCAACCAGTTATATTCTGGCATCCACACACATGCCAGCGTATGAACTTATCCAGTCCCATGTACAAAATAAATAATAAATTAAAATTTTAAAACACATTGTACAAGTAAACTAGTAAGGACATTTGATTGCATATATGACTAAAAATCAAATTAATTGCAGTCAATATTTTCATGATTTTTTTCAAGTGCAAATGGATGCAGAAGTCAAAACCAGACATCTCCTTATCTAGCTGTTTTATTTTTTTGGTTTTTTTTGTTTTGTTTTGTTTTCTAGCTGTTTTAAATTACACATGATTCTATTATTTTATATTAAACACTATTAAGAAGTCACAAGTTTAATATTGAATCTCAACCTTCAGACTGATAGATTCTAACATGTGAAAGGGTTGCTTTGCCCAAAAGTTTCATGAGCACAGGCTCCTGAATCCTGGGGCTTGTGAAATGACCCTTCAAGTAGAGGTGATTGCTGCCAGGTCTGGAAACCTGAGCCTAAACACCCAAGTTATACACACATGCACTCAAAACAAAGAAAAAAGTATAAAATTATTAACCACATATACCCTGACTATATAATAAACTTTCTAGATTTTTCATGATGGAAAGGCTTTTAATTTTAAGTGATGGTATGTAAAAAAAAGAACATTAAATTATATCTACAGAAGCAAAACCTTATCCTGGGAATCAAGACTGTGTGCTCCGTGTTTAAATAGACAAAGGTTAGAACCTCGAGTTCCACAAACAAATGTGAGTTCTCTTTCTGTTGGTTAAACGCCATGTCATTGGTCATGTCAACAATGTGATTTGAGCCTACATTAGTGGTGTATCCACAAGTGCATTTGAGCCTCCGTAGTATTACCAATATTTCATTCATGGTTCAAGTTCATGGGTGTTGAAAACGTGATTGAAAAATGGTAAAACTCAGTATTCTTCCCCCAATATCTGATTGGCAAAAAGGATAATAGGGAATAGATTCTAAAATGTTGATTGGCACAACACACAAAAAGGTTCAGTTGGGAGAAATCCACACATAGGTTTACAAAAATATAGGAACTGGTTGTGCCCAGAAAATAAAGTCCAAGTAGAGATGAGAAATTGTATTTTTGTGTAACTATCAGCTACATGTAAGTTTTAAGTTTCAAAATCAAGTTTTAACAGTGCCCCTTCCAGTTAAAATAACATGATATCATCTTGGGTTTTGTCTTCCGTTTTCTTAATAGGCACATAAAGTCAATGAATAAAAATCTATCTGAAACATGGGACAGCAATCAAGCATCAGAAACTGATAATAAAAGTTAGTTGTCCATCCCTAGTGAAGTTCTCCAGGAACCAATCTGCATAGTAAATGACCCAATGTCAACGCCAGCACACAACTGCCAACTAGGATGCTGGACACTGCTCCAGCCAATCATTCTGAAAGGCTGCTATAAAAGGCAAGGGGGTGACTCTGAGCCTTATCAATCAGTGTGCTCTGGGCACCTGCAGGGCAGACACAGTCACTCACACACAATGAAGGTCATCATCCTCCTACTTATGCTCAATCTGCTCGGTGTCTCCAGTGTGATGATGAATAAAAATCTCAAAAAGAAGGTAGTAGGCCTGCATGGGATGGGGCTGTGGGGGGCGGGTGTTCTTGTCAGAGACAGACACAGGCAGAGAGACAAGAAGACACAGAGAGAGGCATGGGGGACCGTGGGGCAAGACAGACAGAGAGAAAGAGGCAGAAAGACAAAGGGGAAAGTTGGTATTTTCTATGTCTGGGAGTGGAGAGTGACAGAGGAGTAGTCTAATGGCTGGTTTTCTGAAAGAAAAAAAACAGAGGGACAGAGACGAAGAGATAGAAACAGAAAGTAGAATGCTCTGGAAGTTAGCATTCTATACTTGTGAGAGTAGAAAGAAGGAAAGAGAGAAAATAGTCTTAAAGATGGTTTTTGGGGATAGAGACCTAGAGAAATAAAGAGGCTAAGAGATAAAGAGAGAAAGACACAGATTAATGCTTCTGAAGGTAAGTTTTCAGAGTGTGTTTGTGTGGAGAGAGATGGGAGAGAGGGAAGAATGTTTTGGAAGTAAGTATTCTTAATGTCTCTGTGGAGAGTGGCAGAGAGAGAGAATGGACTAAAGAGTGGTTTTTCAGAGAGATAGAGACAGAAACCAAAAGAGACACTGATAAGAGACATGGAAAGAGAGACAAAGAGAGAATGTCCATCAGTCTTGCACCAAGGGTGAGTTCAACTTTCTACTAAAATCTGTACACCTTCATCTCTTTGACATTTCTACTCCTGTCCCTCTTCCCATGAACAAATGGAAACCATGATGATAGATACAATAAAATAATTGAAAGAGGCTGAATTGTACTAATGTTTCTGTTTGCAGTGGCCACTATTTACTTTGGCCTGTTATCTAAAATTTAAAAGTAGGACTCACACAGATCTGCAGAAAAATCAAGATGACTCAATTCAAGCAAGAGTTCAGATTACAGACAGACACATTTCCAAGATTGACAGTAGGCCACATGACTGACAGGACTCAAGGGCCCCAAGTTATCATGTGCATCTCATCCAGGGATCCCAAAGGCAAACAAGCTGGTTACTAAAGGTCCTTTTTTATTTTGATTAACAGATTAAATCATTTATTTCTCCAACTGCATGACTTTAATCATACATATGCCCAATATTACACAGAAGCATTAAGTGACCAGTAGGGTAAAGTTATTTGAAAAGCATGTTTTTGTCTTACTGGACATACACTCAAAACCAGATCAGTGTGTCTATTCTTCCTACCTGAATACATTGAGAATGTACTTGAATAAAAACTGTCCAGACTTGATCATTGCTAGCGATTTGGGAAATCCTACACCATCTTCAGAGAGAGATGTATGAGTAGCCAAATGCAGGTGGGAAGGGAGTTATTAATGGGTTCTTATGTGAATATCACTACACAGTTGTTCACAGATGGGTATGAGAGTAGCCTGACCCAGATGAGTAGGGAAGAGAAAAGATTGATATATGGGGAACTCTATACCATTATTCAGAGAGGGGTTTGTGAGTAGCCTGAAACAAGTGGGAGGGGATAAAATCATTATTAGGTTCATTGCTCAGAATATGTACAAACATAAAGAAGCTAAGCCACCTAATGCATTATTACAAGGCCAAGTCATGGATTACCCCGTTCAAGCCAGCCCAGGTCACCAAACTATCACCTACTCTCAACTGCCTTGTTTTCAAAGCCACTTTTTTTTCAGATTGAAGGGAATTGGCAAACTGTTTACTTAGCTTCTAGTTCTGTCGAGAAGATAAAAGAAGACTCACCCTTGAGAACCTACTTTCGTCGCATTGAATGTGGGAAGAAATGCAAGCAAATCTACCTCTATTTTTATGTCAAGTAAGATATAAAACAAAATAGAACTATCCAGGTTGTGGGATGTCAGCAGAGAAACATGATGTTCTCAGTCAACATTCATGTCAGTGAACACCAACAGCACCAAATAAGGATTCTTAAATCATTTTGCTCTGTATGAGCCAAAAAATCACATATGACAGCCATACTTTTAATTTAGAATGAGTTTATTGAGTGTGTGACAAGCCAAAGTAAAATAATAAATATCAAAAGGTAGATGATAATTACATATAGATGGCATATAAATACATAGATAATAGATATATCAGTACATAGGTGATACATGCATATGGAAGTAGATAGATAATAATTAGATACATGATATATACAGACATACAGATAGACAGACAGACAGACAGACAGACAGATAGATGATATATAAGTGGGTAGATAATGGATAGATGGGCAGATACATACATACATACATACATACATACATAGGTAGATATAAATAATACATGAATAGATAATAGACCCATCTATCTGGGGTGATCAAAGAAAAACACATTAAGCTGTAAAATGCATGTGGGAATCCCTATGTTCAAAACCAGCTCCTAGGGGAAGTTAAACAACAGCATGACACAATATCAAAAAATATGCATTACAATTGTTTAATTTTCTATTTATCAGTGCTCAAAAACACCGTTGGGGAGAAGGTAAATTGTCCCAAATAATAATTTCAGTGCTTTTAAACTATCTGGATATAAGTGAAATTATCCATTGAGAATATACATCTATCTGTGATCATGGAAGCTTTTATCAAATGTAGTTTATATTTGAAACTTTTCATTCATTAATGATTTATTTATAATATCAGTTTTCTGATATAGAGTCTCTGTATATCATGCTGGCTTTGAACTCAACACAGAATAGAAAACAAACTTCTTAATTTTTTATTTATTATGTATGTTGGTTTTTCAAGACAAAATTTTTCTGACTATCCTGGAACTTGCTCTGTAGACCAGGCTGGCCTCAAGCTCAGAGATGTGCCTGACCCTACCTCCTGATTGCTGAGATCAAAATTATGTACCACCACATGCAGCCTAATTTTTTAAGTTTCTTTTTTTATTATTATTACTTTTGAGAATGGCATTGAACCTCCTATCCTGCATCAGTGTCCCAAGTGTTAGGATGACAAGCAATACAAATATACTTGGCTTGATATTTAAATTTAACACACCTGAAATAATTTAAAAGAGCAACAGTTCAAAATCAATTAGGTGAACACACTAACATGTTGAAAGAGAGATAAAATTTTTATTTTTTAAATACTTTTTTAACTAAAACATACTTATTTCCTCCTTCCCTTTTCTCCCTCCAAAGCTTCTCATATACTCTCCTCCCTTGGTTTCTCTCAAATCTTATGTTCCCTTTTCTTCAATTGTTCATATACACATATGGTCTTACATATACAAATATAACATGTTCATTTCATATACTGCTATTGTACAAATATGATCTCAGGGCAATCACTTGGTATTTAAAAACCAAGTGGTTTTGGGGGAGGGAGGGCTTTTCCTCAGGAAAGACTATAAAATATCCTAAAATTCATATGGAACCAGAAAAGACCCAGGCTAGCTAAAACAATACTGATCAAAAATAATAGTGCTGGAGGGATCACTATTCCAGATACAAATGGTATTTCTTAAAGCACTGAAAATGGACCCCAAGACTTTCAACAGGCATTATGGAACTACATCCCCAATCTAAACTGTTAGTTCTGTCTTTTATCTTTTTGGTTGTGAGCCTAGCCTTTAATGGCTGAGCCATCTCTTCTGCCCAATTATTCCCATTTTTAAAAGATAAAGACAAATCTAAACTGTGGCTGTCAGCTAATCATCACATGGACCTCTGGTAAGTGAATGTAGATCCAAGTACATGGCCATTCTTGTGACTCTACGGATCTCCTAATAAGTTAATGGACATCCCAATCTAAATCTCAGAAAACAACTGAAGGCTGATTTTGAGGCTATTTAAATTCACAAGGCAATTGCTGCTACAGAATCTATAAGATCAGTGATATTTATGAACAGAAAATTTTAAAACGTGATTACAGTGTAAATATGGAAATTAGACTATAATGTATGGAGGATGCTGCATTGTACTCTGTACCAGGTGACCTCATACCCTATTAATCCAATGTCTACAAAAATTCCTCATTTCAAATTGGGGTGGAACCTTTGGTCACTACTTTGGTTTTTTTTATTTTATCAATAGTTGTTCTTTGGGTATTGGGGATTCAACTGTGGCCCAGCATGTACCACAGATCCTAGGACTCCATCCTCAGCACACAGGTGTGGCCCTATATGGCCGACATCCCAGGGCTTGGTGAGTGGAGGTGAGATCAAGTTCCAAGGTCATTCATTGGTCAGGGGTCAGGACAGGTGACAAATAGAGGAACTGATTCTGTACCATCAAAGATACCTGCAAACCCCAAACAGGGTTATTGATGACATTTAAGTATGCTATTGATTATATTTACAGATGATGTCATACAACAACGTGTGCTTCTGACAGATCTACAGATGAAAATGTGGTCAGCATTAAACTGTCCTGTCATTCATGTAGAATCCAAAATCAGGACCCTGGAGCAATCCAAAATAAGACAACATCCATGCATTTGTTTTTCAGGAGAGGGACCAACTGCCAGAAGTATGAAGTCATAGGAAAGAGAAGGCAAGAATTTTACCAGGCACAGTGTGAGTAGATGATTCCAGGGCAAACGTACGACCCTAATTTTTTTTAATTCACAATCTTAGTTGTATTTTGCATATCTGAATATCTACATTCCAGTGCAAACATCTCTACAAATTAAAAGTGCAATCTTGGAGAAAGCCCCTTCAATTTCTTCCTTGTCATTTTGGATCTAATAGCAGACTTTGTTTTGCTGTTATGGTTGTTGCTATTTGGTTTTGGTTTTGTTCTTAGGTGACATTTTAAAACATTAAACTGTGTTTGTACCAGGCACACTTCCCTCCTGTAGCTTTAGGCGTAAACTAAATCACAGACATGAGTCTGAGAATGGTTAAAAGAATCAAAAAGTGATTTGAGCATTTAAAAAAATGATGCAAAATAAAAACTAGAATTTTCTCATTGATTAAAATGACTCCCAGAAATTTTCAAAAATGAATCTTGTAATGCATAATTATAAACTAACTAAATTTAATAATTTTACTGGGGATAAAAATTATTTACAGTATATAAGTGTAATCTTCATGAATAGTCCTATCTTTTTAATTCACGAGTATGACCTAAAATATGCAGAGATCCACTTATAAATAATGAATCAGTTTAATTTGTCCTAAAACAGGTGTTAAGAATCCTTTTATTATTTTGTATTCCACCATAAATAGTCAATTTAGCCCAATTCAATAAAACAGACAGACCACACATGCCACCGACTCTAGCTCAGTTTATCAGTGAGTTTATTGGACTTAAATACAGGAGCAGACTCAACTCACATGCAGATACCTCACCAAAAATCTCACCCCCTTTTGTGTATTATGTAATTTCAGGAGACATTCTGTGAGTTTGAGGCCAGCCCGGTCTACATAGCAAGTTTCAAACCAACCAGGGTCTCGGAACGAAAATGACTTTACCACTGACATGAGAAGGAACAGTCATCATATCACTCGGGAGGGTCTTATGTCTGTCCCTTCCCCTCTTTAACAAGAAATGTTAGTAAACCGGTGGGTGTACTGAAGTCACTTACAAGAGTATGTGTGACTCAAATATGGCTGCATTCCTAAGGAGCCCACCCCTGAAGAGTGGGTGACTCTCCTAGAAGCTGCATCTCTGGAGCTGTGTGTAAAGCTAACAGGCAGCTGGGCTAGTCAAAGACTCCTCTGTCAAGAGCTGTTTGCTGTTTTCCATAACTTTGTGGAGTGGCCTACTGAAAGTTTTTCTTTTCAAAAAAACTTCCTGAGTTTCGTGGGTTATTTACTTCTTGAATCTCATGAGCTCCCAATACAGAATGTTCTGGTTTGGAGGAAACTGCTGTGGCATCTCTCTTGTTTCTCTGTCATGTGTCTAAAGAATAGGTAAAGTTAAATAACTGTACCACTAAATATAATAGGATCCATGAAAATGCATATATATGTGTGTGTTACTATATATTATATAATATTACATATTATATAATCATACAGCTTTTCTCAATATGTATATATGTAGATTATTCTACAATTAAATTTAATAGGATTCATGAAAAAGCATGCACATGTATGTATATGCATATAATCATACAGATATCCTCAGAAAGTAAAAGGATTGCAGTAATGATACCTTCTGTGCACCTACACATCCTGGTATCAGGAATATTGCTATCCCAAATCAATATAGGTTCTACACATTTCTAGATAACTATGAAATACGAGATATGTTTCATATCTTTTTTTTTGAATTTTCGAGACAGGGTTTCTCCGTAACTTTTTTGGTTCCTGTCCTGGAACTAGCTCTTGTAGACCAGACTGGCCTCGAACTCACAGAGATCTGCCTGCCTCTGCCTCCCGAGTGCTGGGATTAAAGGCGTGCGCCACCACCCCCCGGCTTATGTTTCATATCTTAAAGCAGAAGTATAAATTTGCTTTCTTAATTCCCTGTCTGTCTTGACTGGTTGCAACACATGGAGCAGAATATTATGGGCCAGAGACCTATAATCCATGTATACATTGATAAAGATGCATTGTGAATGAGACTGGACATTCATCTTGTAATCATTATATTATTTATATGTATATTTCTTATCTATCTCAGATGAAGGAAAAACAACATTCATGGTAAAGATGGTGAATGAGAAGATATTGCTGTTTCATTATTTTAACAAGGACCTTACGGGGAAAGTCACACGAGTAGCTGGAATTCTGGGTGAGTTTCACACATGGAACTTATCATCCAAGTATGTTGTTCAAGGACTTGGGTGAGTGCATATTGCTGTGTACAAAATTCAAAGTATGTTTGTACATCTGACTCTGTTCTTCATGTTGATAAACCGCTGAAACGGTGATGGACAGCTTTAACGAAAAAACTTAGCACAACCCAAAATCCCCTGGAAAGGGTCTGAGTGGGGGATTTCCCACAGCCGACTGGCCTATGGGGGTGTCTTTATTGATTATTGACATAAGAAGGCTCCACCCACTGTGGACAGCACCATATCCTGGCAGGGAGATGATGTTCTGTGAGCTTCAACTATCAACTGACACAACCCAGAATCTTCCAGAAAGGGTCTTAGTGAGAGATTTCTTTTTTTTTTATTGAAAAAAAGGAAAAAAAAAGTTTCCGCCTCCTCCCAGCCTCCCAATCCCCTCACCCCCCCCCCCCCCCCNNNNNNNNNNNNNNNNNNNNNNNNNNNNNNNNNNNNNNNNNNNNNNNNNNNNNNNNNNNNNNNNNNNNNNNNNNNNNNNNNNNNNNNNNNNNNNNNNNNNNNNNNNNNNNNNNNNNNNNNNNNNNNNNNNNNNNNNNNNNNNNNNNNNNNNNNNNNNNNNNNNNNNNNNNNNNNNNNNNNNGGAGGGATATGGGAGGCTGGGAGGAGGTGGAAACTTGTTTTTTTTTCCTTTTCTCAATAAAATATATATATAAAAAATAAAAAAAATAAATAAACACATGCTTCTTCTGTTGAGACATAGATTTTAATAAAATTTCCACACATTTTTTTCATCATTTCTGAATTACACATCAACCAATCATCATGCCAAGTCTCATGTCAGTGTAGTCTGGGATTTCCATTTTCTAACAAAGCAGGAATTGAAATATTGCTTGACCACATTAAGTGGGTTTGTTGTTTGAATTTATGTCGACTGAAAATCCTGGCTTTCAGCTTTATTGTTTCTACTTCACAAATCCTGACTTGCAAATCTTAATTATCACCTAGCCTGAAATCTGATTGTACTGGATTCTGTCAGTTTCTCATAGATGCAAACCCTCTTGAGCTGACAAATTCCTAACCCTGCCATCAAAGGCACTGGAGTATATTGTATAAGCCCCATATTTCCTACCAGAATTGAACCTTTATTTCTTAAGTTATTATGAAGGGATTATATTTCATGGGGGCATGTTACTCATTAGCAAAGGTTTATTCTTTTATGCGTACAGGTGTCTTACCCACATTTAGGTGTACCATGTGCATTCTTGGTGCATATGATAGCCATAAGATGGCATTAGATCTGGTCGTGCAAACTGTGTGTGTGTGCTGGGAACTGAATGTAGGTTCCTACCCATTCTTAACCACTGAGTCAAATGTCACCCCTCCAATATTTATGATGATTTCAGATCAGTTGGTTCAGCAATCTGAGAAAGCATCAAGAACCCGTAAGAATGGATTTTAGAATATAAATGGAAACGATATAATGACATATAGTTGTGATAGTAAAAAATGCTACTGATCAATCATTGTTTTCTGGAATTGCTTTGGAGGCTGTGTAGGAGTTTTGGTATATTGAAGTCTACACCCCCCTCAAAAAAAACTGTCGATATTACAAGAACAAGAGAACATGGCATGTTACATCCTGGAGGCCAAATGTTGTAATATTGTGCTTTGGCTCCTCCAGGAGACTCAGCATTGAAAAGTATTTTATTAATGTGCTGGAGCTAATGTTGTGCCATCTTCTTCCTTATACACCATGAAATGCAGCGAAAGCCAAACAACTGACTAAGGATGAGATGACACAGTATATGGACTTTGTGGAGGAAATTGGCATTGAGGATGAGAATGTACAACGTGTCCTGGACACAGGTAAAATAGCAACCTGAGTGTGTATGACTTTTCATGTTACATTGTATAACAAATGTTATTTTTTTTAATTATCTGGGGTATTGGAAAATGGCTCTGGGGGAAAGTGCACACAAGCATGAGTACCTGAGTTTGGATCCCATGTAGAAAGCCAAGAACACCAGAGCTAGGAAGGGGTGAGGACAGAAGGAGTCCTGGAGCTTTCTGGCAACAAATAAAACTGAAAGATCTTGTTTTAAAACTAAAAATACGTAGGATGAATAAGGAAGATGTTTAAAAGTCAGCCTATTGCCTATATATGCATGGACTGCACACAGGTGAACTCACATGACCATGGACACACCAGTGAACCATGAACATAAAAAATTCTATATGAGGGGGCTGGAGAGATGGCTCAGAGGTTAAGATCACTAACTGCTCTTCCAGAGGTCCTGAGTTCAACCACATGGTGGCTCACAACCATTTGTAATGAGATCTGGTGCCCTCTTCTGGCCTGCAGGCATACATGGAGGCCAAATGTTGTATACATAATAAATAAATCTTTAAAAAATTCTATATGTAATGAAACTTGACTCCCTACTATCCTCTGTTCAGATATTCTACTCAAAACCTCACTTTTCTTTTTGCACTTTCATAAAAATAGCACATATATAGTAATGATATTATTAATATTATGTTAAAATAATGTATTGCTTTAAATATCATTATATTAAAATAATACATTACTTTGATAATAATATTATTATAGTAAAAATAATATCATTTTTCCATTTTCACTTTCTTGCAGACACCTGTCCAAAGAAGATCAAGACTAGGTGAGTGTTAAGCTCATTTTATATCTTGATGTCTAATTCATTTCTGTTTTTTAATAATACTTTATTTGTTGTTTAACTTTTCATATATGTATATATTTTGATCCTATCCACTCCCACACACACACACAATCCCTCCAACTATCCTCAAGACCCACCAATATATGTTCTTCTATACTTTATAGCTTTTAAAATTATTACTATTACTATCATTATCATTATTTGCTACATAAGTCCAATTAGTGCTATTTATGAATGGGGATGAGGCCATCCCAGGAGGCATGGGCAGTGTAATGTACCAGGGGCCACACCCTTAGAGAAAAAAATGATCCTTCCTAACCTGAAGCCATCAAATGTCAATAGCCTCTAGTTAGGGGTAGATACTCCTGAGCCCCTCCCCACTCCACTCCATTCTAGACTGTTGACTGGCTTTATCTTACCTGGGCCTTGTGCAGATGATCATGGTTGCTGTGAGCTCTCATGATTGTAACAGCCATGTCGTGTTCAGAAAACAGAATTTCACAGCTCTCCTTCCAACCCGGGTGACCATTGATGGCTGAGCATCCACTGTCACTTATCCTCAGCATTTTGACCAGCTATGAATGAACCACTCCTCATTGTAAAAAGTTGATGTGAGGCCAGTTTTGGAGTCATACACCTTTAATTCCAATACTTGGGAGATGAATCTCTGTGTTTGAGGCTAGCCTGTTCTATATAGTGAGCTCCAAGACAGCTAGAGTCTCTGTCTAAAAATAAAAACAAAGTAAAAAATGGACTTGACCAAAGTTTAAGGCAGCATAAATCTATGAGTATTTTTTAAGATGATAGCTTGGAAACAGGACAGTTTGTCACCACTGATCTTCCCCTTAGGCTCCATGATCTCCATTGTCTTGGGCTTTTGACAAGAATTACAATAGAAACCCACACCTACTCCTATTCTTCTCTAGACATGAGAGCTCTCCCGTGGAGTTGGCATCAAATCCAATCAGAAAGTGGTTGGTTCCCCTAGGATGTCCTTTATTGCACCAGTGGGCATAAAGCTACTTTTATAACATGCAGGGTCATCATGGTGACATCATCAATGTCTTACTGCCATATACACACCCTACAGAGTACCTCCAAGTACTGTAGAAGCCAGCCAGCAGGGAGAGATTTTTCTAGTTTATTTGAGATTGATTTCTTGATGCCCTACAACCACAGCATGTAGTGTCTTCAGCAATGGTGTCTTACCATTTAGTTATGGTAAACAACCAAGAGATACAGCAATAATCAAGTTGTTTTGGTGACTCCAGGACCTCCCTTTACTTATAAATGATAAGGTGGTACCCCATAACTGGAATTGAGATTTTCATTGAAAAATCCACATCGTCTGGAAACAGTGCTGTGCCTTGCAGGGTTCCTCTAATGAAACTCTCTTGTTTTATGCTACATTTTAATTAGCTTACAAACTAGTAGATTTCTGTAGTTTTGGTTGACCCACCCTACCTCTGTTCTTACCTATGGCCCAACCACATCTACATCTGCTCCTTTGACCCACAGCATGCCACCTCTAATCTTGTCTGTCACCTGTGTTCCATTTTCCATTTTAATTATACTTTACACTTTTGTTACAAGGTCATAGGTTTCCATATGATTTTTTTCCTTTTTTCCCCCATATGAATTTTTAATAACCCTTCATTTTAATTAAACCTTCCATCATGCCCTGATTTTGTCTTTTTACATTCTACTCTATGATTGAACCTTCCCGCACCAGTATTCTTCTTTATACTTTCATTTTAATTGCATTTAACTATCTCTCCTCCCTTGACGGGTCTGTCCCTAATGACCTGTTTCTAATTACCTGGCTTCCATACATACTCCAAATTAAACATACAAAATAAAAAGTACAATTCTAAAATCAACAAGTTAAATAACATACACAGTTTGTCTTTCTGAGCCTGGTTGATCTCCTTGAACATAATATTTTCCAGCTTCTTCTATTTACTTAAAAATTTCATTTTTCTCTATGGCTGAGTAATATTCCATTTTATATACTACAACACTTTCCTTATCCATTCATCAGTTGATGGGCATCTAGATTAATTTCTAGCTATTGTGAACTTTGCTGCAATGAGCATGGATATTCAAGGGTCTCTGTAAGAAATATTAAAGTCCTTTGGGCACGTATCTAGAAATGGTATAGCTGGGTCATATGGAAATTCTGTTTCAGGTTTTTGAACTTTTTGTTTGTTTGAGGTTCACGGTGTTTTATTTGTTTGTTTTAAGTTCTTTGTATATTAGAGACATTAATCCTCCATTAGATGTGTAGCTGTAAAAGATCTACATTACCTAGGATGCCTCTGCATTTAATTGACAGCTTGCTTTGCTATAGTTTTTAATTCCACAATATTTGACTATTTCTGTTCTTACTTCTTTGCCACAAAAATCCTATTCAGTGTAACATGAGGGCCTGGTGCTTGTTGGGGTTTCTGTACCACCCCGTACCCCAGTCATTTAGTTCCAGAGAAAATCACACAGAGGTCTCCATAAGATTATAAACTGATTGGCCCATTGGCTCAGGTTTCTTATTAGCCCTTGTAACTTATATTAACCCATTAACTTTATCTATGTTAGCCACATGGCTCAGTACCTTTTTCAGTGGGGCAGGTCACATCCTGCTTCTTTGGTGATTTGGGCAGGAGGGCGGAAGAGCTTCCTTCTTCCCAGAATACTCCTGTTCTCTTTGCTCTACCTCTACTTCCTGTCTGGTTTTCCCACCTATACTTCCTGTCTGGCCAATCAGCATTTATTAAAATATAATTGACAGAATACAGACAATTATCCCACGCCAATTCAGAGAGTTCACATCTGTGCCTATGATCAAAGTACACTTCTTGCTTTCTCCTCTATCAGTTTCAGACTAACAGGTCTTACATCACAGTTCTTGATCCATTTGGAGCTGAGGTTTTTGCAGGATGAGAGGGAAGGCTCCAGGGCCATTTTTCCACATCTTAAGATCCACTTTTCCCAGTTTGTTTATTCTTTAGCAATTCTATCTCTTTATTGAGTTATATATACTTAGTTTAGGTCATTTTCCTTCTTTCAGTGGAGACATTGACAATATCCAGAAGGTGACTAATTCTTACTAGTGTTGAAATGGAATTCTCATCTATGGTCCCAGTGTTGGGGAATGCAAGTTCTACCTTTAGTCTTCCTCCGAGAGTCAGTCTGGGACTTTGAGTGAGATTCCTGCTGTAGGGTACATGTGGTAAACCTCAATGTGAAAGAACCTTGAAACAAGCAATTATATTAGTTTAGATTCATAGAAACTATGGACTCAAATAAGTACATAACTATATAAATTCTAAGAAATCCAAATTTAGGTCTTGATGTAAGATCATTTATCATATTAAACTTTTCCATATATAGGAAATTTCAATTAAAATTCATGTATATTATCAAACATACAAAGTCCTGTAAAATGTTTTTGCATGATACATCTTGTCTCTTTAATATCTTGTATTTCTTGTATTTTCCACTCTCATCAAATATCATGCATTACTATCTTAAATATATTAAATAATTCTGTTCCAACCTTACAGATGACAGCATCAGGAATTTTCCTGTAAATCCTTCCTGAAGCCTGAAACATCAATATGAAGATGGAGCTTCTTTTCTCTTAGAAGATCACATCTCCCTATTTACTGTATATTACAATTCCTGTCTCCATATTTTCTCTTTCATCTGTCCTTTCCTGTGTTCTAATCAGGGTTAGTGCATCTTTGAATATTTAAATAAATTTTTTCACTTGCATACACTTCTTTGAAGAAAGAAAGCTAAAGCACAATGCACATAAGTATCTATTGGACTTTTTTAAAAGGAGAGGAGGGTTGGATAGATTGATTGAAGGTTAAGAGTGCTTGCTGCTCTTCCAGAGGAAATGTGTTCTATTATTAGTGCCCACATTGAACAGCTCACAACTATAGTTTTGGGGAACCTGGTACCCTCTTCTGGAGTCCTTGAGCACTGCACACAGATTACACACACACACACACACAGAGAGAGAGCATAAATCTTATTCAAGTATGGATTAATAATATCAGATAATTTTGTATTAATAACAATTTCAGGGATTAAGGTGTAGCTCATTTGTTAAAGTGTCTGCCATGCAAGGGTAAAGCCCTGTTTTCAATTCTTAGAATCCATGTGAAAAGCTGTGTGGTGGGCCATGTCTGTAAATTCAGCACCAAGGAGGGAGGTTGAAACAAATGAATTCTCTGATGTCACCAGGAGTGAGCCTAGACTACTTAAAAAAATCACAGGCCAGTGAGACACCATAACAAAAAATAAGGTGGATGTGCATTAAAGAATGAAAGATGACACCTGACTTTGTTCTTTGTCCATCGATGTTCAGATGCACACACGTGCATCCACAAGCACAGGTGTTCAACTATACACACACATACACACACACACACAAGAATTTCATCATTCCTGCATGTTAAAGACTCTGCTACTATTGTTTTGACTTTTAGAAAGGAAGGTAAATTATCATATTGGCTGCTAAAGGTGAACATTAAAAGGTTTGTTTGCTATTAGTTTTATATCAACTTGGCACAAGCTATAGTCACTTCGGAAGGGAGATACTCAATTGAGAAAACGTTTCCACATGGTTGGACTGTTGAAAACGTTCTGGGATATTTTTTTGATAAATGGTTGATATGGGAGGGCCCAGCTCACTGTGGAAAACACTGCTGAGCCAGCCAGTAAGCAGCACTCCTACATGGCCTCTGAATCAGATCCTGCTTCCAAGTTCCTGCCTTGACTTTCCAAGGTGATGGGCAACAAGCTGTAAGATAAAATAAACCCATTCTTCCCCAACTTGCTTTTGATCTTGGTGATGTTGGAGATCTTAGGTTAAAATACATTTTTCTTCTTTTCCTTCCTCCTCTCTTTCTTTCTTTCTTTCTTTTTCGTTTGTTTGTTTTTCACGACAGGGTTTCTCTGTGTAGCCCTGGCTGTCCTGTAGCTTGCTCTGTAGATCAGGCTGGCCTTGAACTCAAAGATCCACCTGCTTCTGCTTCTCGGGTTTTGGGATTAAAAGCATTCATCACCACCACCCAGCCACAAATAATTTTTTAAAAGAAAGTTGAAACCAAATGCTCAAATACCCAACTATATGGAAAATGTAATCTAGTACAGAACACCATAATCATCTGATTTTTATTTTTCTATTGAATACACCATGCTCACATCACCTCTCTCTTGTTATCCTCATTTCCAGGAATTTGTCAAAAATATATCTTTCCAAAATCTTTTACTGTCCTTCTTAATTACTGTATTTACTCATGTGAAACCATTTATCAAATACCTAAAATAAACACATATATGTACTTGTTCATAAATAAAATACAGTATCAAAGAATCAGTGGAAGTCCCTTTTCCTATTGATTTTCATGTGACATGTTGAAGAGTTTACAAAATAAAATTGCACATTCAGATAGCATTTTTCCTTTAATCCCAGCTCTTGGGAGACAGAGGCAGGTAGATCTCTTTGAGTTCAAAGCCAACCTGGTCTACAAGACCTAGTGCCAGGACAACCACCAAAGCTACAGAGAAACCTTGTCTCGAAAAAAAAAACAAAAAAAGAAAAAGAAGAAAATAAATGCAAAGGAAAGGAAATGGGTTCTGTTGAAAACAGTGTCAAAATGAGCTGTAAATTCAGTCGTTTTGGAAATGATCCAGTAGTAGAAAACCTTTGGGGATCAGTTGATGGGTTTGTTTGCAAAAGCCTATTGCTATCTAAATACTTAGAGAAAAGCTGGGAAATAGTACTGTATTTTGCCAAATCATTTGAGAATGATGCATGAGCATAATGTAGTAGCATCATTCCTACCCTTCCTCTTACCCTCTAACTCTTGTGTCCCTCCACAACTCTTCAAATTCATGATTTCTTCTGTAATTACTTTTGTTTTAGTTAGATATATGATATATACATGGATACATAGACGCATACATATATATGTGCTTAGTCAGTCATTAACTATATAGATACATATATAACAGATAGGTGATTGATAGGTAAATAATTGATAGATTATCAGGTAGATGATGATAGATAGATAGATAGATAGATAGATAGATAGATAGATAGATAGATAGATAGATAGATAGATAGATAGNNNNNNNNNNNNNNNNNNNNNNNNNNNNNNNNNNNNNNNNNNNNNNNNNNNNNNNNNNNNNNNNNNNNNNNNNNNNNNNNNNNNNNNNNNNNNNNNNNNNNNNNNNNNNNNNNNNNNNNNNNNNNNNNNNNNNNNNNNNNNNNNNNNNNNNNNNNNNNNNNNNNNNNNNNNNNNNNNNNNNNNNNNNNNNNNNNNNNNNNNNNNNNNNNNNNNNNNNNNNNNNNNNNNNNNNNNNNNNNNNNNNNNNNNNNNNNNNNNNNNNNNNNNNNNNNNNNNNNNNNNNNNNNNNNNNNNNNNNNNNNNNNNNNNNNNNNNNNNNNNNNNNNNNNNNNNNNNNNNNNNNNNNNNNNNNNNNNNNNNNNNNNNNNNNNNNNNNNNNNNNNNNNNNNNNNNNNNNNNNNNNNNNNNNNNNNNN
