# Supplementary material for: Comprehensive Analysis of Rodent-Specific Probasin Gene Reveals Its Evolutionary Origin in Pseudoautosomal Region and Provides Novel Insights into Rodent Phylogeny
Source: Biology (Basel). 2025 Feb 27;14(3):239. doi: 10.3390/biology14030239 (PMC11940140; doi:10.3390/biology14030239)
Supplement: Supplementary file 1 [file biology-14-00239-s001.zip › Suppl Data Files/gPBSN/gPBSN_Nannospalax galili.docx]

>NW_008352538.1:c162778-140000 Nannospalax galili isolate Female #2095 unplaced genomic scaffold, S.galili_v1.0 scaffold3005, whole genome shotgun sequence

GGTAGGATTTCAGGGTGGTGCCTGAGGCCGTTGAGCTCTTTCATAGATGTCAAAATTTGGGTTTTTGGACTATAATGGAACAGGATTAACATGGAAGTGAGAGAAGAGTAACTTTGTCCCAGCAATTAAAGGCTATATTCTTGTCATGTGGAAACTGTCATCACAGCTGTTTGAACAGTCAGGAGGACTAGACCACGATCCTGTGCAAACGACCCTGCACGGCCTCTGGATGCGTTGAGCACGGTGCTCTCGGTGCTTTTCCCACTGTGACCTGTGACTTGGTGTACAGGGATTCGCACAGGTGTGTGTGAGTTTTTCCACTCCTGCGGCTAAGCCTCAGGCACACGTCTTCAAAAACTGACCTGAGCGTGTCAGGAGAAGCCAATATGCACCTTGTGTCCCAATGCCGTTTACCGTGAGGACAGAGATTCGGGGACAGATGCCATCACCTCCAGTCTGTCGACTCAGAAGGACACCAGGCCCCGGATTCTGAAGTGTGGATGGGCATAAAGCCTCAGGGTTGAGAGTGCCCGGCTCTAATCCCGGCACTGGGGAGGCAGAGGCAGGAGGACCTCTGTGAGTTCGAAGCCAGCCCGGTCTACAGAGCGAGTTCCAGGCCAGCTGGGGCTGCGGGGAGGCCCTGTCTCCAAAAAACATGTTTTTTTAAAGCAGAGAGACAAGGCTGAGTTTGGAGGAATTCCTGGCTGTCGGCACACGCAGCTGTGAACACGCAGGGCACGAATCATGCCCAGACACGGACATGACACGGACATGACACGGTCACATGCCAGAGCACCCGGACCTTCAGGGCTTGGAACCGCACAGACAGAGCTTGGGGTGTTCGGTGCACAGCATTGAACTGTGCGTCTCCATTAGACATCCGAGAACGCTCCCTGCCCAGCTTCACGGGTCGGTGCACACGGGCCGACCAGGATGTAAGGAGACCTGGTGCTTCGGGCCACACAGGCCGTCAGAGCGAGGAGAGGAGCGGCCGGCGTCCGGCTCCGTGCCAGCGGCCAGCGGCGGGGCCGCAACATGAAGCTCATCCTGCTGATGGTCGCGTCCGCTCTGCTCTGCGTTTCCTGCAGACAAAATAAGGACATCATGGAAAAGGTACCGGACACTGCTGTAAGGGGACCGTGAGAGAGGGGGNNNNNNNNNNNNNNNNNNNNNNNNNNNNNNNNNNNNNNNNNNNNNNNNNNNNNNNNNNNNNNNNNNNNNNNNNNNNNNNNNNNNNNNNNNNNNNNNNNNNNNNNNNNNNNNNNNNNNNNNNNNNNNNNNNNNNNNNNNNNNNNNNNNNNNNNNNNNNNNNNNNNNNNNNNNNNNNNNNNNNNNNNNNNNNNNNNNNNNNNNNNNNNNNNNNNNNNNNNNNNNNNNNNNNNNNNNNNNNNNNNNNNNNNNNNNNNNNNNNNNNNNNNNNNNNNNNNNNNNNNNNNNNNNNNNNNNNNNNNNNNNNNNNNNNNNNNNNNNNNNNNNNNNNNNNNNNNNNNNNNNNNNNNNNNNNNNNNNNNNNNNNNNNNNNNNNNNNNNNNNNNNNNNNNNNNNNNNNNNNNNNNNNNNNNNNNNNNNNNNNNNNNNNNNNNNNNNNNNNNNNNNNNNNNNNNNNNNNNNNNNNNNNNNNNNNNNNNNNNNNNNNNNNNNNNNNNNNNNNNNNNNNNNNNNNNNNNNNNNNNNNNNNNNNNNNNNNNNNNNNNNNNNNNNNNNNNNNNNNNNNNNNNNNNNNNNNNNNNNNNNNNNNNNNNNNNNNNNNNNNNNNNNNNNNNNNNNNNNNNNNNNNNNNNNNNNNNNNNNNNNNNNNNNNNNNNNNNNNNNNNNNNNNNNNNNNNNNNNNNNNNNNNNNNNNNNNNNNNNNNNNNNNNNNNNNNNNNGAGAGAGAGAGAGAGAGAGAGAGAGAGAGAGAGAAATAGAATTTTTTAAAAAAGAGGTGGTTTTCTCTAAGTCTGGAATGACAGAAAATGTTATCAAATGTAGTTTTTTCTGAGTCTGAGGAAATGAGACTGGGGGTGGAGAGAGAGAGAGAGCGAGGGAGAGACAGACTGAATGTAATAATGTTGTGATTTCTAAGGACTTTGCTCTTAATCTTTTTCTGTGCCAGTTAAAGAACTAAAAGGTGCGGGGGAAACTGTCAGATTCAGGGAAAAAAAGCAACAGCAGATTAGAAGCTGTGTAAAGGGTGCAGATCCAACACGACACCGCAGTCACACGTGTAGACAGAGGACCGAGAAGCTGAACCCCAGACCCCAGCACTGGTCGTAGGGTCCCCTCCCCGGGCCCGTTTCTCCCGTTCATCAAAGAAGGCAGCACCCAGCACGTCCCCCTCACGGCGCCAAGGATCTGTCCCTGTGTGGCCTTTGCGTCATCTAAACTAGTCTCGACCGGTCCCAGGGGTGGGGGTGGAGGGGTGTCCGTGCTTCTGTCCAACCAAGAAGCAGGAATTTGGTATTTCATTGTTTGTGAGTCTGCCAATCGGTACTTAGTCCCTTACAATGAAGGGGGGATGTCTCTGTGTCTGAGAGAGACAGAGCGAGAGAGTGAGACAGAGATGGAGAGAGAGACATAGGGAGACAGGAGAGACACAAAGAGACAGGGAGACAGAGACAGACAGAGACAGAGAGACAGAGAGAGACACAGTCAGAGAGACAGAGAGATAAAGATAGATGGAGAGACAGAGACATAGACAGAAACAGAGATAGAGAGACAGAGAGACAGTCAGAAAGACAGAGAGATAGAGACAGAGACAGAAAGACAGACAGAGAGATAGGTGGAGAGAGACAGAGACAGAAAGAGACATAGAGATACAGAGTGAGAGACACAGAGAGACAGAGAGATAGATGGAGAGAGACAGAAAGAGACACAGACAGAAAGAGAGACAGAGAGACAGAGAGAGACAGACAGATAGGTGGAGAGAGACAGAGACAGAGAGAGATAGAGATAGAGAGAGAGAAAGAAAAAGACAGATGGAGAGAGGCAGAGACAGACAGACAGAGACAGGCAGAGAGAATGAGAGACATGCAGCCAGAGAGGTGGACAGAGAGAGGCAGAGAGACACAGAGACACAGAGAGATGCTGTAAGGAGTGGTTTTCTTTCAAACACAGAGACAGAGAGGGATAAATAGACACAGAAAGACACGCAAATAGAAGCAGACAGGGAGACAGAGAGAGGGAGAGAGAGACAGGAAGAGACAGGGACAGAGAGAGACAGGGAGAGAGGGGGGAGAGAGAGGGGGAGAGACAGGGAGAGAGAGGGAGAGAGGGAGAGAGGGAGAGAGACACGGAGAGAGAGGGAGAGAGGTAGAGAGAGAGGGAGAGACAGGGAGAGAGAGGGAGAGAGACAGGGAGAGAGAGGGAGAGAGAGGGAGAGAGAGGGAGCGCCTGGAAAAGGTCATTTTCTCTGTCTCACAGAAATCAAGAGAAAATGGGCTAAGCAGTGGTTTTGTCTATTTCTGCAGAGAGAGAGAGAGACAGAGACACAGAGAGAGACAGAGAGACAGACAGCGAGGCGAGAGAACATCGTAACGTCTGTTTTTCTCTCTCTCTGAGCAACTCAGAGACAGAATGGAGACTAATCTAAGGAATCATTTTGTCTGCGTCTGAGGGAGACGGACAGACGCCCAGACTGCAGGGGTTGTGAGAACTGGGTTTTCTCTTAGTCAAGAGAGAGATGGAGACAGAGAGAGACACAGACAGAATGATGGTGAGAGAGGGGGGGGGAGAGGGGGGGAGAGAGGAGAGAGAGGGAGAGACAGAGAGAGAAGAGAGAGGAGAGGAGAGAGGAGACAGAGAGAAAGGGACAAAGAGGAGAGAGACAGAAAGGGAGAGAGAGGACAGACAGAGGAGAGAGAGGGGGAGACAGAGAGAGAGGAGAGAGAGAGAAAAGGAGAGAGAGGAGACAGAGGGGGAGACAGGAGAGGAGAGAGAGGAGGGGGGGGAGGAGAGAGAGAGGAGGGGGGGAGAGAGGAGACAGAGAAAGGGAGAGAGGACAGAGAGGAGAGAGAGGGGGAGACAGAGAGAGAGAACACTAAAAGATGTGGTTTGTCTAAGTCTGGAATGACAGAAAAGATTACCAAATCCGAAGAGGCGACGGCGAGGGGGGGCAGGGTCGGGAGGAGGGAGGGTGAGGGAGGAGACAGAGAGAGGGAGAGAGGACAGAGAGGAGAGAGAGGGGGAGACAGAGAGAGAGAACACTAAAAGATGTGGTTTGTCTAAGTCTGGAATGACANNNNNNNNNNNNNNNNNNNNNNNNNNNNNNNNNNNNNNNNNNNNNNNNNNNNNNNNNNNNNNNNNNNNNNNNNNNNNNNNNNNNNNNNNNNNNNNNNNNNNNNNNNNNNNNNNNNNNNNNNNNNNNNNNNNNNNNNNNNNNNNNNNNNNNNNNNNNNNNNNNNNNNNNNNNNNNNNNNNNNNNNNNNNNNNNNNNNNNNNNNNNNNNNNNNNNNNNNNNNNNNNNNNNNNNNNNNNNNNNNNNNNNNNNNNNNNNNNNNNNNNNNNNNNNNNNNNNNNNNNNNNNNNNNNNNNNNNNNNNNNNNNNNNNNNNNNNNNNNNNNNNNNNNNNNNNNNNNNNNNNNNNNNNNNNNNNNNNNNNNNNNNNNNNNNNNNNNNNNNNNNNNNNNNNNNNNNNNNNNNNNNNNNNNNNNNNNNNNNNNNNNNNNNNNNNNNNNNNNNNNNNNNNNNNNNNNNNNNNNNNNNNNNNNNNNNNNNNNNNNNNNNNNNNNNNNNNNNNNNNNNNNNNNNNNNNNNNNNNNNNNNNNNNNNNNNNNNNNNNNNNNNNNNNNNNNNNNNNNNNNNNNNNNNNNNNNNNNNNNNNNNNNNNNNNNNNNNNNNNNNNNNNNNNNNNNNNNNNNNNNNNNNNNNNNNNNNNNNNNNNNNNNNNNNNNNNNNNNNNNNNNNNNNNNNNNNNNNNNNNNNNNNNNNNNNNNNNNNNNNNNNNNNNNNNNNNNNNNNNNNNNNNNNNNNNNNNNNNNNNNNNNNNNNNNNNNNNNNNNNNNNNNNNNNNNNNNNNNNNNNNNNNNNNNNNNNNNNNNNNNNNNNNNNNNNNNNNNNNNNNNNNNNNNNNNNNNNNNNNNNNNNNNNNNNNNNNNNNNNNNNNNNNNNNNNNNNNNNNNNNNNNNNNNNNNNNNNNNNNNNNNNNNNNNNNNNNNNNNNNNNNNNNNNNNNNNNNNNNNNNNNNNNNNNNNNNNNNNNNNNNNNNNNNNNNNNNNNNNNNNNNNNNNNNNNNNNNNNNNNNNNNNNNNNNNNNNNNNNNNNNNNNNNNNNNNNNNNNNNNNNNNNNNNNNNNNNNNNNNNNNNNNNNNNNNNNNNNNNNNNNNNNNNNNNNNNNNNNNNNNNNNNNNNNNNNNNNNNNNNNNNNNNNNNNNNNNNNNNNNNNNNNNNNNNNNNNNNNNNNNNNNNNNNNNNNNNNNNNNNNNNNNNNNNNNNNNNNNNNNNNNNNNNNNNNNNNNNNNNNNNNNNNNNNNNNNNNNNNNNNNNNNNNNNNNNNNNNNNNNNNNNNNNNNNNNNNNNNNNNNNNNNNNNNNNNNNNNNNNNNNNNNNNNNNNNNNNNNNNNNNNNNNNNNNNNNNNNNNNNNNNNNNNNNNNNNNNNNNNNNNNNNNNNNNNNNNNNNNNNNNNNNNNNNNNNNNNNNNNNNNNNNNNNNNNNNNNNNNNNNNNNNNNNNNNNNNNNNNNNNNNNNNNNNNNNNNNNNNNNNNNNNNNNNNNNNNNNNNNNNNNNNNNNNNNNNNNNNNNNNNNNNNNNNNNNNNNNNNNNNNNNNNNNNNNNNNNNNNNNNNNNNNNNNNNNNNNNNNNNNNNNNNNNNNNNNNNNNNNNNNNNNNNNNNNNNNNNNNNNNNNNNNNNNNNNNNNNNNNNNNNNNNNNNNNNNNNNNNNNNNNNNNNNNNNNNNNNNNNNNNNNNNNNNNNNNNNNNNNNNNNNNNNNNNNNNNNNNNNNNNNNNNNNNNNNNNNNNNNNNNNNNNNNNNNNNNNNNNNNNNNNNNNNNNNNNNNNNNNNNNNNNNNNNNNNNNNNNNNNNNNNNNNNNNNNNNNNNNNNNNNNNNNNNNNNNNNNNNNNNNNNNNNNNNNNNNNNNNNNNNNNNNNNNNNNNNNNNNNNNNNNNNNNNNNNNNNNNNNNNNNNNNNNNNNNNNNNNNNNNNNNNNNNNNNNNNNNNNNNNNNNNNNNNNNNNNNNNNNNNNNNNNNNNNNNNNNNNNNNNNNNNNNNNNNNNNNNNNNNNNNNNNNNNNNNNNNNNNNNNNNNNNNNNNNNNNNNNNNNNNNNNNNNNNNNNNNNNNNNNNNNNNNNNNNNNNNNNNNNNNGGTTTTCTCTCTGAGACAGAGAGAAAGAAAGAGAGAGAGAGAGAGAGAGAGAGAGAGAGAGAGAGAGAGAGAGAGAGAATGTTCTAAATGGTTGTTTTCCTTATTTCTGAGACACAGACAGAGACCAAATGTGCTAAGTGGTGATTTTTCTGTAAGTCTGGGACAGAGACAAAGAGCAAGAGCAACAGAGAGAGAGAGAGAGAGAGAGAGAGAGAGAGAGAGAGAGAGATATTGTAAGGGGTAGTTTTGTGTATGTCTGAGAGACAGAGACAGAGAATATTATAAGAGGTCATTTTTCTTTAGGCCTGAGAGAGAGAGAGAGAGAGACAGAGAGAGAGAGAGACAGAGAGAGAGAGAGACAGAGAGAGAGGCTTCCTCTGTGTACAGGGTGAAGACAAGTCAGATTGCTAAGCAAGCTCAGCCTCTCGACTTTGGATTCATGAAACAACGAGGTTGGTCTGTCCGTGCAGGAAAACCCCGGGAACCAATTTCCTCTACTACTTAATAACTCGGTGCTGACATTTCAGAAAACTCTGCATCCCAGCGCCTCCAGGCAAATCAGCAGAACAGTCTCACGTCTGTGCTGCAAGGCACATCCCTCACCCCGCGCGGCAGTACCAACCCCAGAGTCGAGCTTGGGTGTTTCTAGGTTCTTGGTGTTTTATTCTAAGATGTGAAAAATAAGAGCTCACAGACATCATCAAAAGAAGCCGCATAATTTTACAGAGATATTATAAAAGAATGACTATATATATGAATGAATGAATGAATGAATGACTATATATATGAATGAATGAATGACTATATATATATGAATGAATATATATATGAATGAATATATATGGGGTGGCTCCCAAGACATGTGATCTGACTGGCTGTCTGTCTTGACTGATAGATGAGGTCATACGTTCACGTTATTCCGCGTGTCGTTTCCTGTAATGTGTCCAGTCTTAATCATACGCATATCCGATGGTGCGGAGGATGGTCGGTAGCCTTCCTTCATCACCCAAAGAACGCCACGTGTCCCGCTGGACGCACACCCAAACCCAGCTCATGTGGGATTAGCGTTTGCCTTTTTCCCACCTGGATAGATTGGGAATACACTCGAATAGAAAATGTATAAACTTGATCACCACGGGGCGTGTGGAAGCCCAGGGTATTATTGTTCAGAGACAGGTGTGTTCAGAGCCTGGGGTAGCTGAGGGGCTGCTGAGGGAATTCTTTGCAGAAGGGGGTCTTTGTGTGGTACAGATAAAGGGGCTTCTGAATGACCCTCATCCAAGGACACCCCGGGTTACTAAATACTGATGATGCCCCCTGCATCCCCTCGCATCTTTCTAGATTACGGGGAACTGGACCACTGTGTACTTGGCATCAAGTGCTGTGGACTTGATCAAAGAAGACGGACCCTTGAGGACCTACTTTCGTCATATTGGATGCAACAATAAATGCAAGATTCTCAACCTGTCTTTCTATGTCAAGTAAGTAAGACAGAAACAGAAAGAGACACTCCGCCGCAGCCAGCTGGGCACGGCGGGTCCTCACACAGTATTTCCGTGATGACGCTCGGCAATCACCGTTTTCCTAGATTGATTCGAGCGTTGCTGAATTTGGTTAAGCAAAAATGTGTGTCTCTGAACTTAGTGTTTTCAGATATAGTCAAACATATCAACACACGTGTGAAGATAAATAAGTATGAGACAGAATAATGCTTTGATAATAGGTCTTCCACCCACCTCCGACAGTGTCTTTTATGTCCTTAAGGAATGATGTTTTAGAACCAAGACATGGACGGAAGGTATAAAAATCCAAAGGCATATATGTTGGAGTCACAGCGAGGCCAGAATGGTTTGATGTGCAGCGTGCTGGTGTTCAAACTGCAGGAGCACTGTTACAGAGCATTGCGCTACAGTCAAAACTCGGGTTGAGACGGATAGTTCCCCAACACTACATTTTTCTGAAGCGTCTCCCTGTAGGCCCAGCTGGGCTGGCCCTCGCTGTGTAGACCAGGTTGACCTCCAGCTCACAGAGATCCACCTGCTTCAGCCTCCCGAGTGCTGGGATCAAAGGCGTGCACTGCCATGCTTGGTGCGCTGTGACTTTTCTGTGTCTCTTCTAGGGACTCAGTGAAAAGGACTGGGGAGCTTTGTTCTGTGTGTCCTGAGAACGAGCTCTGACGGCCACGCTTCGGGTGTTAAATGCAAGAGTTTATCAAATACACAGCAAACCACGCTTTAAGAAATACACAGCGTGTGGTAGATACAGATAGAGGATTCAGAGACGCATAGAAGATAGAGACAGATTAGATAGAAGATAGATGGAAGATAGATGATAGATAGATAGATAGATAGATAGATAGATAGATAGATAGATAGATGATAGATAGATAGATAGATGANNNNNNNNNNNNNNNNNNNNNNNNNNNNNNNNNNNNNNNNNNNNNNNNNNNNNNNNNNNNNNNNNNNNNNNNNNNNNNNNNNNNNNNNNNNNNNNNNNNNNNNNNNNNNNNNNNNNNNNNNNNNNNNNNNNNNNNNNNNNNNNNNNNNNNNNNNNNNNNNNNNNNNNNNNNNNNNNNNNNNNNNNNNNGATATGCTCATATGTAACAAATGCAATAGATGTTAAGGCGTGTCTGCCCTGTGCTCAAAGACCAAAAGCTAAAAGCTTTCTGAAGGATCAGGAACAAGAGGTATATGTTCAACCTCCACATTTCCATGAACCTTCATAGTTGGTTATACAATTCCAAATGGTCAGCCATAAACATATGTACACATATGCATTGCTACATGAACTCAGTAACTATACAGATAGCTACATGATAGATACAGAGATAGATAGATAGATAGATAGATAGATAGATAGATAGATAGATATCAAAACCTCATGAAATCAACCACTGAACACACCCAGCAGAAACTGCCTGGCAAAGGTCCGTGTCAGGCGTCCAGCTGCCCTCCCCAGGAGAGAGCACCCACAGAGCAGTTCTCCCCTCGGGAACGTCCAAGCAAAGAAACAAACAACAGCCAGGGAGTCCCGCCATCCAATCCTGGGTTTTCGTGCTCAGAGAAGAAAGCAGGTGGGGACACCTCCCTGGGGAAACACCTGGGCTTGGTAAACCATTCGGGAGCCGTCAGAGGGTCCCTGTGGCCGAGTCCTCCAAGGTGACCTTTCTGTGTTCAGGGGGCAGGGGACGCCCCATGAGCAGAGCAGTGTTTGGAGGAGACGGTGAGACCGTCACTTTTGAGTCTGAAATGTGGGAAGACGCTCCTGAATCAGCAACAACTTCCCCACGTTATCACTGCTCATCACTCCTCAGGGAGCCATGTGGGAGCGAGAACACACTGACAGCACTGTGGGAAGTACTGTGCATATGAACAAAATTACCACGGGGACGAGGGTCCAACTCTCCTAGACTGAGGAACTCAGTCAATCGCATACCGATATTTGTGTTCTATTTTTTCATTTATTGATTATAATTCTGTTTTCGGGAGACTGAGTCACGTGTAGTTCACATTGGCTTTGGAGTAGACGACACGTCCCTTACACGTATGTCCCAAGCATTCGCATGACGGGATGCACTACGAAGCTCGCCTTCATAGTCAAACTCAGCACAGCTAAAATGATTAAACAGCCGATACCTCGAAATGTGATTAGACGTTCACACAAATATATAAGAGCTAAAAACGTCCTTTCTTCTTTTTTAAAAAACTATTATCATTTTTATTTTATGTGTATGACTGTATTGCCTACATGCGTGTAAGTGCACCTGTGTCGTGCAATGCCCACGGAGGTCAGAAGAGGGTGTCGGATCCCCTGGGACAGGAGTCGTAGCCGGTTGTTAGCCGTCTTGCGGGTGCTGGGAACGGAACCTAGGTCCTCCACAAGAGCAGCTAGTAATCTTAACGGCTCCAGTACAAACGTCCTTTCTTCTTTTTTAAAAAACTATTATCAGTGTTAGTTTATGTGTATGACTGTATTGCCTGCATGCGTGTAAGTGCACCTGTGTCGTGCAATGCCCACGGAGGTCAGAAGAGGGTGTCGGATCCCCTGGGACAGGAGTCGTAGCCGGTTGTTAGCCGTCTTGCGGGTGCTGGGAACGGAACCTGGGTCCTCCACAAGAGCAGCCAGTAATCTTAACGGCTCCAGTCCAAACGTCCTTTCTTCTTAAGTGTTGAGAATGCAATCCGAGCTCTTCAACCCATGAGGCCAGCACCCTGACACTGAGCCGCACAGCCAGGCCAAGTGGTCCTTGGCTTTTTTAAAGTCATAAACATGAATCTAAAGTGTGGTTGGAGATGCCGATCCTCATACTGTAGGTCACGGGCGATTGCTGGGTGAACAGTCGTGTGAATTGTCAGTGTTTCTGTCTTACTTCTTCTGTCTCCTGCTGTCTGTACTGACTCGAACCTTCTTAGCAAGCAACCACGGGGCGAATGCGGAGTCATGTCCATGATCATTCATGGAAGGCTTACTATCGAATCTCCAAAATCAGCAACATTTTAGAGACAGGTAGAAACCGTGTGATTTCAATGGACATAAAGTAAGCCAGAATTTCATGTATCTAAAAAGGAAGAAGAAAAAAACTCGCACTGTACCCTACAACACACAACAGCACGACCTTTAAGTCCTCATAAATGCCGATCAGCGTATCAAGTTTGTTTGCTATTTTTATTTTTATTCAATTTTTCTGAAATTGTTACAAATCTCACGCAATATACTTTGAACATATTTAGATCCCCCCCATAATTGCACACCCCTTACTAATCCCCATCAAATTTCTGTACTTTAATATGTGTGTGTACATGTGTGCGTGTTAAGCCCCATAGAGTCACGTATGCGCCCATTTCCCTGTTTGTCCACCGGAGCCTGGTCAACCTACTAAGAGTCACACCCTTAAATGAGACCGACTCACCCCCTCCCAGCTGTCATCTGTTGCCAAAAGCACTTGCCTAGCATGGAGCCCTGAACTCCATCCTGAGTACCACATGTAACAGGTGTGGTCATGACTGAATCTGACCCCGGCACTCATCAGGCGGAGGCAGAAGGATCAGTAGTTCACGGTCGTGTGACGGGAATGCTCAGAAATGCAGCAGGTGTCCCACGGCACACAGAAAGGCCGTCACCACTCCAGCAGACATTTATCCAAACCCAAGGCCGTAATTGATTTTTTTTAATGTCATAATACTATAACAAAGGCACCGTAAGCTATGTACAAAAGGACTATTAACTTGAAAATATATCTTATTGTGAGCACTTGACACGTGTTCTAACTCTCGTGGTTTCTGCCATCCCGAGACGGGGCTCTAATAACAAACGATAAAAAATGTCTGTTGCTTTGTTTTTAAGGAAAGGGGGGAAATGCAAATTCTTCAGAATCCCAGGAAGAAAAGGAGATAAAAACGTTTACGAGGCATCATGTGAGTAAGAATTGCTGCTGTTCTGGTCTTAACTGTATTTCTGTATGTCTAAACATGTAGGTTCTAATGCAGCTTTTATTAGAAATTAGACATGCAGTCTTAGAAGAGCTACTTAAATCCCCACCTATACTTTTTAGGTACAGTAACAGGATACTTTTTTAGTAATTTATTATGTTTATTTTATGTGCATGACTCCGTGGCCTGCACGTGTGTAACAGCAACCCTGTCATGCAGTGCTGAGGAGGTCAGAGGAGGGTGTTGCATTGCCTGGAACTGGAGTTACAGACGGTCGTTGGTCGGCACGTCACGTGGGTGCCGGGAATCGAACCCGGGTCCTCTGCAAGAGCAGCCAGTGCTCTTAACCCCTGAGCCATCTCTCCAGCACTGAGAATACTTTTAAAATTTGAAACAAGGGTCCTCTGCAAGAGCAGTCAGTGCTCTTAACCCATGAGCCATCTCTCCAGCACTGAGAATACTTTTAAAATTTGAAACAAAAAAGGTTTTGCACTTATTTATTTATATTTATTTATTGTGTGTGTGTGCATGTGTATGTGTGTGTCTGTGCATATGGAAATCAAACTCAAGTCATCAGACTTGCTGGCACGCACTGTGTTTAAAATTCTGACAGGAATATGGCCTGGCCATGGCTACTTACAGTCTAACTTAAAATACGTGGCGATCCTTTTATCATGGCGTCTTTGCTGTTTCATGGCAAAGTAAAGTGATCCTTATCTCCAATTCCATTTTAAAGGCTCCATTCACCCCAAATACAGTCAAAACCAAAACACCAAACATCCCACCAGCCCCATTTGGGTTTATGAATCAGTTTATCGGAATTATTGATAAGAGCACAGACGGCTTACGAGATTGCTAAAATATCCTAAGCCCTACTCCTTCTCAGCCTGTCGCTCACTAAAACCTTTTAAATGTGCTTTTGTTTTATGAGCGGTTCACCTGCGTGTATGTAAGTGTGGCGTGTGTTTGCAATGCCCACAGAGGCCAGAAGAGGGCGTCAGAGCTCCTGGAACTGGAGCCACAGGCAGTTGTCTCAACCTCTTCTGTACTTCAGCATTTGTTATAAAATTACAGACACGATAAGCAAGAGAGAGGTGATCATATCTCAGAGGCCAGCCTGGCGAGGGTCCTGGGACCGTCACTTCGTCTTCTGCAAGAAAAACATATTAGCAGGCCCATTACAGCAAATCCACCTTTTTCCTGTTCAGTTGTTGACCTGTCATCATGACCGCTGGGCCAAGTCTCCTGTAGGTCCAGCTTAGGGAGACCCTGAGTTTCTTGGAGCTACTTACAGGAGGGCGACTGACGGGTGACTTATAGGAGGATGGGCCCCTCGAAAACCCAGGTGACAAGTCGTGAGAGCTGCGCACGTGGAACTGTGTGTGCCACCCGCAGGCAGCAGGGGGTCGTCGGACAGTGTCCTCCCCCAGCAGTTGTACGCTGCCTTGGCACGGGGCCTCGAGAGTCTCATTAGTTATTGAATTCTCGAATCTCATCCGGGTCCCTCTTCCCTCCATGAGGGAATGTTTCAGTCCGGAGAGAACTTCTAGGTGACAGGCCCTCTTTATGGTCTTTTCCTATTCCTCACTTGAGGGAGACCTGGTAGGGTTCGGTATTTCCACCACGAAGTTAGATGGGAGGCGTACACATTTTCCTTTTGTTATACAGCTCTGTTGAGCACATACAGGCGCATGGCAAGACTCCACCATCCACATAACTACGCACCCCGACATTAGGAACATTGGAATTCCAAGTCAGTATCCATCCCAGCACTGTGTGGGCACCTGCAGAAAACCGATATCGTTCCTATTTTAAGGAGGGAGTACAAACTCACTTTCCTGATCCGTGGTCTTTCTCTTCACACACTGTACGTAGAGGTAGGAATATTAGTGAGGCAAGAATTACGCTTCATGTCAGTGTCAACAAGCGTTTACTAAACATGGGACCTGCGCAGTCACCTTTGCAAAGTTCGCTGCATCAAATCCACACTTTTTTTTTCATGTATCTCAGTTGAAGGAAAAAATCGTTTCATGATAAAGCTGGTGACTAAGAAGATGTTGCTGTTTTATTACTACAACGAACAGAACAGCAACGTTGGACGTGTTGTCGGAGCTCTCGGTAAGCGTTGCGTGGAATAAAGTGCGAAGTTTATTGTGTGTGTGTGTGTGTGTGTGTGTGTGTGTGTCATTCGAGGGAGATGGGTGTCTGTGCATTATCTTACAGAACAGCAATATTGGACGTGTTGTCGGAGCTCTCGGTAAGCATTGGGTGGAATAAAGTGCGAAGCTTATTTTGTGTGTGTTCATTCGAGGGAGATGGGTGTCTGTGCATTATCTTACAGAACAGCAATATTGGACGTGTTGTCGGAGCTCTCGGTAAGCGTTGCGTGGAATAAAGTGCGAAGCTTATTGTGTGTGTGTGTGTGTGTGTGTGTGTGTGTGTGTGTGATTCGAGGGAGATGGGTGTCTGTGAGCTATCTTCCACTGAGTTCATGGGTGCCAGAGTCTGCTCGGGAATGAGATTGAAGTCTTCATCTTCGTGGGTCAGCGATAGTGTGACGGTTGTTTTTGATTATGAAGTTGACACAATCTCCAGTCAGCTGGAGTCTCGTGGAGGGACCGGCTGTATCAGGCCGTCCTGTGGGCCTGTCTGTGTGGACTGCCTTAGTAACTTAACGTAAGGAACCCCGCTGCGTGCAGTGTGAGCGGCACCGTTGCCTAGGCCTCAGGTTAACTGTCAGCTTGACACAGCCTAGAGTCACCTGAAAACAGTGTCCATGAGGGATGGCCTACACCAGGTCGCCCTCTGACCCCGTCTGCAAAGGCAGTCGTGACTTGCTGGGACAGAAGACGTTCTTTATGATTAGTTCAAATCAGTTGGCTCTGTCCTATGATGACGAGTCCAGAATCCCAAGGCGCGCATTCTAGAACGCAAATACAGGCAAAGTAAGAAGTTGGAAGGGAGGCGGTGATCAGTCAGCCACAGCCTTTCTCATTTTTGGATGTTTTGGAAGCTGTATGAACCTTAGGGGCACTTGAGAACACAAGAAATTCTTAATTCCCTAAGAATCAAAAACTGATGGAAACTGATTTCACAGCCATGGTCTGGCCCTATATTTTCATATCACATGTGGACACGGGTAAACATCATCCGTCGGCCCCCAGACAGAGAGGCAGTGTTGTGACATGTCCCGTCCGGTAGGCTCACGTCCGGTAGGTCCTCTGTAAGCGTGCTGCGTCCTGACTGTCGTGTCTTCTCACATCCCAGCCAGAGCCAAACAGCTGACTGAGAAGCAGATGGAAAAATACATGGACTTAGTTGCAGAAATCGGCATCGAAGATGAAAACGTCCAACGTGTCGTCGACACAGGTAAAGTCACCACACGACATGGTGCGACACCGGCATTTACCTGACGGGTTCTGGTGATGACTACATGGGCTGTGCAAGCACCGGGGCTCGAGGTTTCCGGTACCCAGGTTTTTAAACAAAAAAAGTCAGAAATTCGAGTTCCCAGAGGGGGAGATGGCAGAAGCCCTCGCAATCGCCGGCTGGCTAATCTTTCTGAATTTCCAAGTTCCAGGTTGGGCGAGCCCTCTATCCAAACAGTAGGGTGGGGAGGGATTCACAAAGGCACTGGGATGTTCACCTCGGACTTCCACGTGTGTGGGGTGCACCTGTACACACAAACACACAGAGAAAAGAAAAACACGGGATCACCCACTCGACCACATGCACACATATAAACCGCACACACACAAGCACACACAACATTTATCTGCACTGAAAGCAGCTTTCTAAGGTCAGAAGTTCTATTCAAATTCTTGCGTTGTGTCTGAGGGCAAATGAAAATTCCTCACCGGTTGTAATAAACTACAAGGATCTCATTTTGTACCTTTACTGTCTTCCAGACACCTGTCCTCCATAAAGACGATACTTGGGTGAGTTGAATCGTGATCGTTTTATCTCCTGATATCTAATCAATTTGGGTTTTTTTTAGATAATACTTTATTTTGTGAAAATTTAGTATTTGTGTACAGCGTGTCTTGATGTTATCTGAACCCCCAGAACATTCAACTCCCCTAGGGACCCGAGAACACGCCTCCCTCTCAAGTTCACACAGTCCGTGACCGTGTGCAGACAGGCGTGGGATCTCCCAGCGGAGTGTGGACAGAGTAAATAACAGGGCCATCCCCCCAGAAAACTGGCTGTCGCTCTCTCAGCAGCCATCCAATGCCAGTATCTCCTCAGCTAGGCGTAGAACCCGAGGAATCTTTTGTCGGAGGCCTGCAACCCACAGCTGCTGTGACACGTTCCGGTGTGCAGTGGCCGTGTCAGGTCCCGAGGACAGCATCTCCCAGCTCTCCTCCCGCATCCTCTGGCTCTCACGGTTTCTCCTCACTCTTCTGAAATGTTCCCTGGCTCTTGGGAGACCAGAGGTTCCTGTAGACAAACCATTTACAACTGAGCAAAAGTTTGAGCAGTTATAGGTCCGGACTAAGCATTAAAAAAAGGAAAAAAAAGTTTCTCAGACCAAGATTGAAATCAACACCGGTTTATGAGTGTAAATATAAATATTAAGAAGGTAGTGTGGGGACAGAGCAATTCACGGAAACAGCACCAGGTTGTCCCCCACCCCGAGCCCACCGTCTCCGCAGTCGGTTTTGTCTAGGCTGACAGTACCAGACGTGGGATCCTCCGTTGACTCTCCCTTGTAGAGAGGTGTCGTGGTTGCAGGAGCCTAGACTGTCATGAAATGACCTACGTCTTCCAGTAGCAAAGTTGCTACATGTATGTGAACTCTTCCTTATGCATCATACTATCAATGTTATCTGACAAACTCTTGCATCTGCATCGAGCTTTGTCGGACACCCCCGGCTGCGTCTCCCCCACCCCCACCTCCCGCTCTCTCGTCTCCCCGTTCCGTGATTACACCCTGGATGGGTCCTTGCCCAGTGGCCTGTTTCATAATTTCATTCTTCTTCACGGCCTAGCAATATTCCATTTACTTTCCCGTCAGATGATGGACGTCTAGGCTGTTTCCGTAGGCTCTCCAGAGTCATCCCTCGCTTGGTGCTGGGGGGTTACGGGTTCTAATTTTCCTGAAGAAAAATGCCGTGCCATAGGTAGGTTTGGTGCAGAACGTGACGCACGCTGGGACGTTGGGGTAAGCAGCCGTGACACCGTGAGGGCTGGGCTCGTAGGTGCAGCAGGTNNNNNNNNNNNNNNNNNNNNNNNNNNNNNNNNNNNNNNNNNNNNNNNNNNNNNNNNNNNNNNNNNNNNNNNNNNNNNNNNNNNNNNNNNNNNNNNNNNNNNNNNNNNNNNNNNNNNNNNNNNNNNNNNNNNNNNNNNNNNNNNNNNNNNNNNNNNNNNNNNNNNNNNNNNNNNNNNNNNNNNNNNNNNNNNNNNNNNNNNNNNNNNNNNNNNNNNNNNNNNNNNNNNNNNNNNNNNNNNNNNNNNNNNNNNNNNNNNNNNNNNNNNNNNNNNNNNNNNNNNNNNNNNNNNNNNNNNNNNNNNNNNNNNNNNNNNNNNNNNNNNNNNNNNNNNNNNNNNNNNNNNNNNNNNNNNNNNNNNNNNNNNNNNNNNNNNNNNNTATATACGTCTATAATTCGTGTATATCCAGACACATAATTCTAGGAATTGATTTGTGCATGATAATCTCTCCATTGGTCATCTCCCCAATAACTTGCATTTTTTTATCCTTTCTATTCTGAGCAAATACCATGTGATTACCATCTCAAATACATTAAACGTATACTTTTCTCCAGCCTTACAGTTGACAAGGACATCGGGAATTTTTCAATCCCTTCTTCTGGAAACCAGGAATCTGGATACGAAAACGAGGCCAACTGTTTATTCAGATCCTACAATCTTCTTTCCCGTGAATTTAGGTCGCCCTGTCAGTTCTTCCTTCCGTCCCTCCCTTCCCGTTTGATGACTGGGGTTAGTGGTTCTGCAAATGTTTAAATAAACTTGCGGTGTGCACGCTTGAGTCTTTGAAGTAAGGAAGCTAAAGTGGAACTATTTGAACGTCAGTACGGCTTCATAATACTGGGTAATTCTGGGGAAGATTAATTTGAGGGGCTACAAAAATGGCTTAAGGGTTAAGAGCACCAGCTTCTCCCACAGAGGACCCGAGTTCAATTCCCAGCCCCCACAGGAAGGTGACAACCACCCGTAACTCCAGCTCCAGGGGATACAGTGCCATTTTCTGGACTCCACAAGTACCGTACTCCCATGGTGTGCGTGCCCAATCCTGGTTCCATTCCCAGCACGCACACGGTGGTACCGTGGACTGCATGGAGGGGTGACCACACATAACTCTCGCTGCAGGGCATCCAGTGTCCCCTCCTGGCCTCCATGGCATTGCATATACACGTGGAACAGACACATACATGCAATTTAAAATAAAATAAGTTTGTGAGGCTGCGGACACAGCTCACACTCACTCACAACAGCACGCACTCACTCACTGGCCTTCGAGAGGACTCGGATTTGGCTCCGACACCACGTGGAGGCTCAAGGTCATCCATAAGTCAGTCCCAGGGGATCCAATGTCGTCTTCTGGCTTCCACAGGCATTGCATGACATGAGTGCACTTATATACATTCTACAGGCAAAACACCCGTAGCCATGGTCATGCACTGGAGCACGGTTGAGATATCTGGCCCGGCATCATTAAAGAAAGACTGACTCTCCTTCTCCTGGTATCAATCAACTGGCAACATCTCCTCACCTAACAGTGGGATGCTATGGCCTCCTCTTCCCTACATGCTGGGATTTTCTATCACTTGCAATAGCAAAGATTTAAATAAAAAAAAAAAAAAGGCCGGGCATGGTGCTGTTCACCTTTGATCCCAGAGCCAGAAGCAGGCGGAGGTCTGTGAGTTCGAGGCCAGCCTGGTCTACATAGTGAGGTCTAGGCCAGCTGGGGCTACAAGGAGACCCAGTCTCAAAAAGACCCAAAAAAAAAAAAGGACAAAAATTTGGGGATACAAAAGGGGGGTTGGAGCTGGAAAGGGCGGAATCTGGTCAAAATACATTGTGTAAAACTCTCAAGGAACTAATTAAAAAAAGGGAGAAAAAGCTTTGCTTGTGTGCAGAAATACGGCAGCTTCTGGACCCATTTTGGGAAGCAAACTAAAACTACTGGAAACAGATTTTTTTGGAAATATTTCATTTACTGTTTTTACTTTACGTGTATGAGTGTATTGCCTGCGTGTATGTGAGGGCACCCGTGTTGCGCAGCCCCGCGTATAGGTCGGGAGAGGGCTTCGGATTCCCCTGGAACCGGAGTTGAGGATGGTTGGTAGCCGCCACGCGGGTGCTGGGAATCGAACCCGGGTCCTCTGCGACAGCAGCCAGCACTATGTCTTAGTGACAACCTGAGTTCAATATAACTTTGTTAGGTTCTCCTTCCAGCATGTAGATTCCAACTAACAAAGCTGTTCTCTGACCGCCACGGCTCATTGTGGCATGTCCACACACGACACACACACAAATATTTTGTTTTCTTTTTTTTCAAACAGCTCTGTCTGGCCTCGGCCTCTGCCTCCCCAGTTCTAGGATTAAAGGCATGTGCCACCATGCCAGACAATAAATGCAATTTTTTAAAACTTTAAATAAATAGGACCCAGGGTATAGAGCTCAGTGGTATGACACTTGCCTAGCATACTCCAGGCCTAGATTAAAACCACAGCATTAGCCAGGCGTGGTGGCATACACCTGTAGTCCCAGTATCTGGGAGGGAGAGACAGGAGGATCTGAGTTCAAGGCCAGCCTGGTCCATATTGCGATTTCCAGGCCAGCTGGGGCTACAGGGAGACCCTGTCTCAAAAAGTTAAAACAAAAACAAAACAAAACAAAACAAACATTGCACAAACTAACAAAAACAAAAATCCACTAGCGTTTAACAGATAACCAAGGGGAAACAGGGAAAGGCCAGACACCAAAGCCAAAAGGGGCAGTCGAGCCTCAGCCTTCTAACAAAGCCCAACCAGCCGAACACTTTTCACCTCCCACAAGTGGGGCTCAGAACCACAGGTTCTCCGCTACTTGCCAGAATTCTGAGTAAAGAGAAGCCCCCATTTTCCTGAGAACACATTATATCAACAGTCCCTTCCTGATAGCTGAAGCTTTGGAAGCAAGCTTTCTATGCCATTACTAAAAGGCTACTATGAGAGCGGGATACAGGGCTGGGGAGACTGTTCATGCAGAGGACCCAGGTTCGGTTCCCGGCAGCCACATGGCAGTTAACACCCATCTGTAAGTCCAGCTCCAGTGGCTTCTTGATGGTCTGTTCTGGCCTTTTGGGGCATTGCACACACATGATGCACTTTGCAGGGTGCAGGGAAAACATTCATGCGGATAAAATAAAAATAAATAAATAAATAAAAATAGTTAAATAATAAATATAATAAATCAATAAAGATAAATATTTGAATAAGTGAAAAATAAATATAATAAATCAATAAATAATAGAGAAACAGAATGTATGCCTAAAAGTATTTGCTGTTAAAACACGGTCCTGAACAGAGTCTCCCTGTGACCCCGGCTGGCCCGGAGCTTGCTGGGTGGACGGTGCTGGCCTGGAGGTCAGGGAGATCTGCTGGGCTGGGATCTAGGACGTGTGTCACCACGCCCGGCTTTAGTCTTTGATTTCTTATCCCTACTTTTCCACCCTTAAAACTTCATCGTACATATATTTTTATGCAACCAGCTTCAAGTTATTCTGGAAAAAAATGTGTTAAAAATTGCCTCTCTTTGGAATAATGAAAATATTGTTAAAAACAATAATGTTTTTGGAGGAAAAAAAAAGATCCTGAAAATTGAAGCCAGATCCCACTTTGAAAGAATCAAACAAATCTCTCTCTCCTTTCTCTCTGTCTCTCTCCTCTCTGTCTCTCTCCTCTCTGTCTCTCTCCTCTCTGTCTCTCTCCTCTCTCTCTCTCCTCTCTCTCTCCTCTCTCTCCTCTCTCTCTTCTCTCTCTCCTCTCCTCTCTCTCTCCTCTCCTCTCTCTCTCCTCTCTCTCCTGTCTCTCTCCTCTCTCCTCCTGTCTCTCTCCTCTCTCTTCTCTCCCTCTCTCTCTCTCTCCTCTCTCTCCTCTCTCTCTTCTCTCTCTCCTCTCCTCTCTCTCTCCTCTCCTCTCTCTCTCCTGTCTCTCTCTCCTCTCTCTCCTCTCTCTCTCTCCACTGAGCCTGGGAGATTAGTGAGCTTTCAGGAGTGTGAGTCGTGTTCTACAAGTAGTGAGTTCCAGGACAGGTGTGGCTACAGGGGAAACCCTGTCTCANNNNNNNNNNNNNNNNNNNNNNNNNNNNNNNNNNNNNNNNNNNNNNNNNNNNNNNNNNNNNNNNNNNNNNTGGTATAAGAAATTAAAAAGAAAAAGATAAATACATGATTGACTGACTGACTGACTGACTGACTGACTGACTGACTGATTGACTGTAGTAAGGAAACAATGTTCATGTATGTAAATCCTGAACATGGGTGCTGGGAACTGAGTCGGGGTGTTTTGCTAGAGCAGTTCAGGCATCCCCACCGCTTAGCCAAGTCTAGGGTGCCCTGAGTTTTTAAACCTGGAATGTGTTCTCAGGAAGCTTCCAGCCCCATAACACTGTCCGCCTAGTCTGTTCTTTAACAATCCAAGGCCAAGTGCCAGGGCACAGGTTCCAGGAACCTGACGGAACCGGTCCGGCGGCCCAGTGTCCTGAATCTGCCCGGAGCAAGAATTGGGTGACAGTCCTGGGGCCCAGTTCCCTGCTACTCGCTTTTGTTTGTGTGTTTGCAATGAGAAAACCTTTTAAAGCATTTGCTTCTCGTCCGGGTGAAGGGACAGTGTTCCCACAAAACACATCCTCCTGGCACAGACCCGGGGAGACCGACTCCTGCTCCGTCCACACTGCAAGCACAGCCCTGACGTCGGCCTCATAGACACAGACGCTATTTGAGGAATGGCTTGGACGCTTCCAGGATGGCCCTGGGGCAAGGACACCACGGTTTCTGTAAAAGTCCAGAGCCTGTTCCCTTCTTGAACCACGTGGGTCCCAGGGACCGAACTCAATTTGTCTGACTTGGCTGCAATCACCTTTAACTCGCTAAGCCATCCCGCCGGCCCCAACTGCCAACTTTCAGAAAGTTCCATGCACAGCACTCGGGAGGCGGAGGCAGGGGGATCTCTGTGAGTTCGAGGCCGGCCTGGTCTACATGCTGAGTTCCAGGGCAGCTGGGGCTACAGGGAGAGCCTGTCTCAAAAAGACAAAAAAAAAGTTAGAAGTAGATCTCCCACATGACCCAGCCATACGTCTATCATCGATCGTGCATCCATCTGCCCGTCTACTCATCTATCATCCGTCATCTATTGTCTATCGTGATCAATGGATCACGTATCGTCTATCTATCAATCATCTCTCCTACAGCTATGAATCCATCTATCATCTATTACGTATCTATCTACCATCTATGCATGTAACCCTGTCCGTCTGTGCACCCATTTGTCTGCAACCTACCTGTCCATTTATCTGTGTATTTATGTGTGCATCTATGTAGCGATTGATCTGTCACCTATCATGCATTCCTGCATCATTTACCAATGCATCCACCTCTCCCTCCAGCCATACATTTATGTATTCATATATCCATGCATGCATCTGTCCATCCATCCATCCGTCTGTGCGAGTTTTGATCCATGCGTGTACCCGTCTATCCATCCATCCGTCTCTGTCTATCCATCGACGCATCCGTTCATCCTAACATTTTCCACCTATGTATACAGCTCTATATACATCTATCCGTAAATCCGTCCGGGCATCATCTTTCTTTTAATCGCTCCGTCTCTCCACGCGTCCATCCTTCTATCCATCTGTCCACCTATCCATGCGTCTATTGATCCACACATGCATGTAGGTAGAGAGCAACATAGCTGTATAGCCATCTGTGCATACATCCGTCTATCTATACGTGTATGCGTTCATCTATCCATGTATCCCTCTGTCCATCCATCCATCAGCTGTCCACCCACACATTTACCTGTCCACATGTCCAGCCATCCATCAATCTGCTATCGACCCACAC
