# Supplementary material for: Comprehensive Analysis of Rodent-Specific Probasin Gene Reveals Its Evolutionary Origin in Pseudoautosomal Region and Provides Novel Insights into Rodent Phylogeny
Source: Biology (Basel). 2025 Feb 27;14(3):239. doi: 10.3390/biology14030239 (PMC11940140; doi:10.3390/biology14030239)
Supplement: Supplementary file 1 [file biology-14-00239-s001.zip › Suppl Data Files/gPBSN/gPBSN_Phyllotis vaccarum.docx]

>JARWLC010000013.1:87278000-87293000 Phyllotis vaccarum isolate GD2095 Scaffold_13__4_contigs__length_150834676, whole genome shotgun sequence

AGGAATGGATAATTGGGACCGAATTCTAAAATGTTGATTGACACAAAAAATGAAAGGGACCAATTTGGAAAAATCCACACACAGCTTTATAAAAATATAGGTGCCAGTTTTTTCCAGAAAATAAAATCCAAGTAGAGATGAGAAACTGTATTTTTGTTTAATCACCAGCTACATGTAAGTTTTAAGTTTAGAAATCAGGTTTAAACAATGTCTCTTCTAGTAAAAATAATATGATAGCATCTTATTTTTGTCTTCTGCTTTTTTGATAGGCACATAAAACCAATGAATTAAAAAAAACTACCTTAAACATAGGACAGGCCCTGGGCATCAGAACTGACAATAAAAGTTAATTGTTTATTTCTAGTAAAGTTCTCCAGGAACCTATCTGTATAGCAAATGACCCAATGTCAATGTCAGCACACAACTGCCAACTGGGATGCAGGACACTGCTCCAGGCAATCATTCTGAAAGGCTGCTATAAAAGGCCAGAGAGTGACTCTGAGCCTCATCAATCAGTGTGGTCTGGGCACTTGCAGGGCAGACACAGTTATTCATACAAGATGAGGATCCTTGTCCTTCTGCTCATGATGGATCTGCTTGGTATCTCCAGTGTGATCATGAATAAGAATCTCAGGAAGAAGGTATCAGGCCTGCATGGGAGAGGGCTGTGTGGGGATGGATGTTCTGGGCATAAATAGATACAGACAAAGAGACAGAGAAACAGACACAGTTAGAGGTGAGGGCTGTCAGAGGAAACAGACAGAGAGGCAGAGGCATAAAGACAAAGAGAGGGAAGAACATTCTGGAAGTTGGTCTCTGTCTGGGAGTGGAGAGTAACAAACTGGGTAGGAGTAGTTTAATGGTGTGTTTGCTGAGAGGGGAAAAAACATAGGGAGAGAAACCAAGAGATAGGAACAGAAGGAAAGAAAGAGAGAGAGAGAAGGAAGGAAGGAAGGAAGGAAGGAAGGAAGGAAGGAAGGAAGGAAGGAAGGAAGGAAGGAAGGAAGGAAGGAAGGAAGGAAGGAAGGAAGGAAGGAAGGAAGGAGAGAATGCTTCTGTGTGTCTTTGTGTGACAGAGAGAGATTACACAAAGGGAGGATTGTTTCTGGAAGTAAGTATTCTCTGTGTCTCACAGTGGGGAGTGGTGGGAGAGAGGAGAGAGAGAGAAAAAACAGTTTAATGGGTGTTTTTTCAGAGATATAGAGACAGAGACAAAGAGATACACAAAGACAGATGCAGAGAGACAAAGAGAGAGTGCCAACCAGTCTGGCACCAAGGGTGAGTTCAACTTTTCACAAAAATCTGTACACCTATGTCACTTCCTACATTCCCACTCATAACCATCACCCTATGAAGAAATGGAAACCTTGATGTTGGATACAATGAAATAATTGAAAGGATGTGAACTGTACTAATATTTCTTGTTAGAGTGTGGCCACTGTCTACTTTTGACTTTTCAAAAGTAAGAACTCACACAGATCTGTACAAAACCATGATTACTCTATTCAAGACAAAGTATAGTGCAAATTACAGATTGACACTGCCAAGATTAACAGTTGGCCACATGACTGACTGGACTCAGAGGCCTGAAGCTATTATGTGCATCTCTTCCAGGGGTCCCAACAACAGACAAGATAGTTAGTAATAGGCCTTCTTTATTTTGATTAACAGATTAAATCATTTATTTCTCCAACACATGTGTCCCTTGACATAATGCATCTGACTTTAATCTACATATGCTCAATATACACAGAAGCCAAAAGTAGACAGTGGTTAATGTTATCTGAAGAACATGTTTCTGTCTTACCATGCATACACTCAAAACCCGTTCAGGCCAGACCTGTATTTCTATTATTCCTACATGAATACATTGAGAATATACTTCAATGGAAATTGTCTGGACTTTATCATTGCTAGGGGTGTGAGAAAACCTATACCATCTTCAGAGACATGTGAGCATCCAGATGCAGATGGAGAGAGAGTCATAAAGGGGTTCTTTTAGGGGAAGCGCTGCACAATTGTTCACAGACAGGTATGAGAGTAGCCTGATGAGATGGGTAGGAGACAGAAGAGGTTATTATATTGGGGAAGTCTATCCCGTTTTTTAGAGAAGGATTTGTGAGTAGGCTGAAAAGGTGTGTTCTAAAATAATTTTTAGGTCCACTGCTCAGAATATACATAAACATAAAGAAGCTAAGCCACCTAATTATTATTGCAAGATCAAGTCATAAATTACCAAATCCAAACTGAGTCCCAAGTTACTAAACTGTTCCCTACTCTTACGTGCCTTGTTTCCAAAGCCACTTTTCTTTCCAGATTGTGGGGAATTGGCAAACTGTTTACTTAGCTTCCAGCACTGTGGAGAAAATAAACGAAGGTGCACCCTTGAGAACCTACTTCCGTCGAATTGAATGTTGGAAAAGATGCAGGAAAATCTACCTCTTTTTTTATGTCAAGTAAGATATAAAGCGAAATAGAACAATCCATGTGGTGGGCTGTGCACACTCATGTTAGTGAATATCCATAGCACTAAGTAAGGATTCCTAAATCACTTTGCTCTGTATGGTCCAAAGAATGACATATGACAGCTATACTTTTAATTTAGAATGAACAAATGTATTTAGTGTGTGGCAAACCAAAGTAAAATAATAAATATCAAGATAGATGATGATTGCTTATAGATTACAGATAAATACAAAGATAATAGATATATCAATAGGTAGATGATATATGCATATGTATACAGATAATAGATAATATACATACATACACATACATATGTAGGTATATATAGATAGATAAAGATAGAGATATATAAGTGGATAGATGATAGATGGAAAGATAGGCAGATAGATAAATAGATACATACATGCATAGGTAAAAAATACATCCATATATCTGGGGTGATCAGTGAAAACAATGAGAAAAACAGGCTTGGGTATAAAATGCATGTGGAAAACTGTTCAAAACCAGATCCTCAGAGAAGTCTAACAGCACAAGACAATTTACCAAGACAATATACATCACAATCATTTAATTTTCTATCTGTCACTCCTCAAAAACAAAGCTAGGGGAGAAGATAAATCAAAAAACAATTTAACAGCTTCAGTGCTTTTAAACTATCTGAATATAAGTGAAAGTATCCATTGAGGATGTATTTATATCTGGGATCATGGAAGTTTTTAGCAATTACATGCTGATATTTCATTAATTAATTATTTATTTATTATTTCAGCTGTCTGAGACAGTCTCAGTACAGAGTAGAGAACAAACTTTTTATTATATATATATATGTTTTTCAAGACAGGGTTACTCTAGCTGTCCAGTTGTCTGGGAACTTGCTCTGTAGACCAGGCTGGCCTCAAGCTCAGAGATCCACCTGACCGTCCCTCCCTCTTGAGTGCTGGGATCGAAAGTGAGTACCACCACACTCAGCCTATCTTTTATTGTTTTTTTTGAGAATGACATTAAACTTATCTTCCTGCATCAGTGTCCCACTTGTTGGAATGACAATTGATACCACCATGCTTGGCTTGATATTTAAATTTAACTTACCTAAGATTATTTAAAAGTGTAATAGTTCAAAATCAATTGAGTGAACACACTATCATATTGAAGGAAAGACAAGATTTTTATTTTTTGAATACTTTTTGAAACTAGAACAAAATTACATCATTTTCCCCTTCCCTTTTTCCCTCCCATACTTCCCACACACTCTCCTCCCTTAGTTTCTCAAACATCCTGTCTCCTTTTCTTTAATTGTTTATATACATATATATATACTTAAATATATAAACACAATTGTTTATTCCATATGATGTTATTGTATAAAAATGATCTCAGGACAACCACTTGGTATTCAAAAACCAAGTGGTTTGGGGAGAGGAAGGGCTTTTCCTCCAGAAAGACTATAAAATGTCCTCAAATTTATATGAAACAAGAAAAAGCTCAGGGTAACTAAAACAATACTAAGCAAAAAGAAAAGTGCTAGTGGAATCATCATCATTCCAGACATAAATGATATCTTTCAAGGCACTGAGAACGGACACTAAGATCTTCAATGGGATTTATGGAGCTACATCCCCTGTCTAAACTGTTATTCCCATTTTTTCTTTTTGGTTTTGAGCCTAGCCTTTAATGGCTGAGCCATGTCTTCCACCCAGTTATTCCCATTTTTAAAGCATATCATTCCCATAATGGTAAGTCTAAGCTGTGGGTGTCAACGATTAGCCTAATTGACCTCTGGTGAGTGAATATAGATCTGAAAAATGGCTATATCTGTGGTTCTATGGATCTTCTAATCAGTCAATGCACATCCCAATCTAAACCTCAGGAAACAATTGGAGGCTGATTTTTAAGCTACTTAAATGCACAAACTAATTGCAACTGTAGAATCGATAAAAAAAAATAGTGATATTCTTGAGAGAGGCAAAAAATTAAAAATATGATTACAATGTAAATACGGAAGGTATGGAGGATGCTGTGGTGTACTTTGTACCAGGTGGCCTTGAACTCTTTTAACCTAATGTTTACAAAGATTTCTCATCTTGGATTGAAGTAAAATCCTTGTTCAGTACTTTGTTTTTTTTTTCTTTGATCAATAGTTGTTATTGGGTACTGGAGATTCAGCTGTGCTGGGAGAGTACTTACCCAGCATGCACCACAGATCCCAGGGCTCCATCATCAGCACACAGTTGTGATCCTATATGCCTATCATCCCAGCACTTAGTGAGTGGAGGTGGGTAGATGAGGTGTTCTAGGTCATTCATCAGGCAGGATTCAGGACAAGTGACAGGTGTCACACAGAGGGGCTGAATCTGGGCCATTAAAGATATTTGCAAACCCCTAACAGGGTGATTAATGGCATTTAAGTATGCTATTGATTATATTTAGTGATAATATCATACAACAATATGTGCTTATGATAGATCTACAGATGAAAATGTGGTTAGCACTGAACTATCCTGTCATTCATATGAAATCCAAAATGAGTACCTTGGAGCAATTCAAGATAAAATAACATCCCTGCATTTTTTTTTCAGAAAAGGGGGCAGATGCCAAAAGATTGAAATCATAGGAAGGAGAAACCAAGAATTTTACCAGGCACAGTGTGAGTAAAAGATGCCAGGGTGAATGCATGACCTTAATTTTTTTTAAATCATTGTATTTTGCATATCTGGATATTCCAGTGCAATCATCTATACAAATTAAATATGTAACCTTGGAGAGAGCTCTTGCAATTTCTTCCTGGTCATTTGGATCTAATAACAGACGTGCTTTGGTGGGTGTTGTTGCTGCTGCTGCGCCTGTTTGTTCTGTTTCCATTTTAAGTGATTTTTTTAAAACCTTATTTATTTAGTGTATTTTTTACCAGCACACTTCCTTCCTGTAGCTTTAGGCCTAAATTAAATCAGAGACATGAATTTGAGAATGGTTAAAGGAGTCAAAAAGTGATTTGAGCATTTTTTAAATGGTGCAAAATAAGAACTAGGATTTTATCATTGATCTAAATGACTCCCAACAATTTTTTAAATATGAATCTTGGAATGTAAAATGATGAACTATCTAAATTTTATAATTTTATTGAGGAGAAAAATCATTTACAGCATGTAAATGGAATCTTCATGAGTAGTCCTATTTTTTTAATTCTGACAGAAATATGATCTAAAATATGCAGAGATCATCTATAAATATTAAAGATATCAGCTTAATTTGTCATGAAACAGGTGGTAGGAATACTTTTATTATTTACTATCTATCATAAGGGGTCTATTTAACCAAATACAATACAACTGACAGACCAAACATTCCACAGACTCTAATTCAGTTTGTCAATGTGATATTTGGACTTAAATACAGGTACAGACTCAATTTACATGGGGATATCTCATCAAGAAGCTCATCCCTCTCATGTTTTATGTAATTTCAGGAGGAATTCCATGAGTTTGAGGCCAACCCAATCTACAAAGCAAGTTTTAGGCCAACCAGAGCTACATAGTGTGACCTGTCTCAGAAGTAAAATGACTTATCATTGACATGAGAAGTAACAGTCATCATATCACTAGGGAGGGTCTTATGTCTGTCCCTTCTCCTCTTTGACAAGTGTTAGTGAACCAGTGAGTGTATTGGAGTCACAAGAGATGTGTGACTCAAATATGGCTGCATTCCTGAAGAGCTCACCAGAGCGCATATGACACACCTAAGAGCTGCATCCCTGGAGCTATGTCAGGCTAACAGACAGCTGGGCTAGTCATCAAGTCTCTTCCCTCAATTGTTATTTGCTATTCCCCATAACTTTGTGGAGTGGCCTACTGAAAGCAGTTATTGAGACTTGTAAGTTATTTACTTCTTGAATCCCATGAGCTTCCCATGCAGAATGTTCCAGTTTGGAGGAAACTGCTATGTGGCATGCTCTCTTGTTTCTCTGTCATGTATCTGAAGAAGAATTGGTAAAGTCAGATAACTGCACCACTAAATGCAAATAAAACCATGAAAATTTATATATTATATATTATGTTTTATATGTAAAATCATATATCTTTCCTCAATATATATGTGTGTGTAGATTATTCCACGATTAAATTTAATAGGATCCACGAAAATGCATATGCATATATGTATATACAGGCATGTGTGTATAAGTATAATCATACAGCTATCCTCAGCAAATCAGGGGGTTGTGGTATGCCACCTTCTGTGTACCTACACACCTTGGTATTAGGAACATTGTTATCCCAAATCAATATAGGTTCTACATATTTCTAGGTAACTGTGAGACAATGGATATGTTTCATATCTTAAAGCAGGAGTATGAAATCACTTTCTTAATTCCCTGTCTGTCTTGACTAGTTGCAACACATGGAGTAGAATATTATGGGCCAGGGACTGTATAATCCATGTATGTATTGATAAGGATGCAATGTGAATGAACCTGAAAAATCATTTTGTAATCATTATATATATTTATATATTTATATTTACTTCTTATCTATCTCAGATGAAGGAAAGACAACATTCATAGTTAAGATGGTGACTGAGAATATATTGCTCTTTCATTATTTTAACAAGGACAACAGGGGCAGAGTCACACGAGTGGCTGGAATTTTGGGTGAGTGTCAAACATGGAACTCATCATCCACATGTGTTATTCAAGAACATGGGTGAGAGCACATCACTGTGTACGAAGTTCACAGTATATTCATACATCTTACTCTGTTCTTCATGTTGATAAACTGCTGAAGCATTAACTTAATACAACCCAGAATCCCCTGGGAAGGGTCTTATTAGGAGATTTACCACAGCTCATTGGCCTGTGGGGGTGTGTTTGTTGATGATTGACATAAAAAAGTTTCACCCACTGGAGGCAGCATCACTTCCTAGCAAGAAGATGATGTGCTGTGAGCTTCACCTGTCAACTGACACAACCCAGAATCCTCCAGGAAGAGTCTTAGTGAGAGATATTTTTTGTATCAGACTAGCCTATGGGAATATCTGTGGAAGTGTCTTAAATGATGATTGACACAAGGAGGACTTTTGCCACTGTGGTCAGTAGCATTTCCTGGACAGACCATCCTGAGCTATATCAGACTCAAGAAAGCTGGATGACCACAAGCATGTGTGATTGCATTCTTTGCTTTCTGTTCCTGACTATGGATGTAGTGTGTCCCCAGATTACTCTCACTGGAATTTCCCCTAATTAAATGTGCTATAACTGGGAAATTATTGCAGAAATAAATCCTTTCAGATCTAAGTTATTTTGTCAAAGAATTTAGTCAGAATATTGAACAAGAAAATTGAGAGTTTATCTGTCAAAAATAAGTATATGCTTTTTCAAAAATAAGTATATGCTTTTCTATTGGGACATAGAAGATTTTAATAAAATTTCCACATATCTTTTTTCAGCATATCTGAATTATACATCAACCAATCATCATGTGCTTCACAGCAGTGTAGTCTGGGATTTTCATCTCCTAACAAAGCAAGAATTGAAATATTACTTGACTACATTAAGTGGATTTGTTGATTGAATTCCTGTCAACTGAAAACCATGCCTTTTAGCATCATTGTTTCTAGCAATCTTTACAATCTTGTAAATCTTAATTATTGTGTAGCATGAAGTCTAACTGTCCTGGCTCTTGTCCACAAAATTCATTTTCTTATAGATGAAAACTTCCTTAAACTGACAAAGTCCTAACCCTGTCATCAAAGGCATGGGAGTCTTTCATGAGACCCTCATTTCATACCAGAGTTAAACCTTTATTTCTGAAATTAATAGGAAGAGATTGGATATCATGGGGGCATGTTCCTCATTCACAGAGTATATTTCATGTATTTAAGTGTTTTACCTTCATTTAAGCACAACATGTGTGATTGGTGCATACAGTGGCTACAAGATGGCATCATATCTGGCCATGCACCACTATGTGTGTGCTGGGAGCTGAATGTAGGTTCTTAAGCATTCTTAACCACTGAATCAATGTCACTCCTCTTGTATTTATGATGATTTCAGATCAGTTGGTTCTGCCATCTTAGGAAGATGTATCAAGAATCCATAGGAATGGATTCTATAGCATATATAGAAACAAAATAATGACATACAATTATGATAGTAAAAAGCTAATAACCAATCATTGTTTTCTGGAATTGCATTGGAGGCTGTGTAGGAGTTTGGTATATTGAAATCAACAACAATGAAAAAAGTTGTCGCCATTACAAGAAGAGGAAGACATGGTGTGTTGTACATCCTGGGATCCACCCAGTATTTCCATTTCTCAGGAGAAGGGCATAATTGCACATTATCAATCTAATGATGGAAACAGAAGTGATGTCATGTTTTGATACTTTCAGGAAGCTAAGCATTGGAAAATATTTTATTAATGTGCTATGTATTAATGTTATGACATCTTCCTTATACAATACGAAATGCAGCAAAATCCAAGCAACTGACTAAGGAGGAGATGACGCAGTACATGGACTTTGTGGAGGAAATTGGCATTGAGGATGAGAATGTACAACGTGTCCTGAACACAGGTAAAATAGCAACCTGTGTGTATGCCTTTTCATTTTGGATTTTATAATAAATGTTTTTTTAAATATCTGAGGTGTTGGTAAGATGGTTCTGTAAGAAAGTGCACACAAGTGTGAGGACCTGAGTTTGAATCCCACCCATGTAGAAAGCCAAAATTCCCAGAGTTGGGAAGGGTTGAGAACAGTAAGAGCCCTGGAGCTTTCTGGCAATATATTTATCACAACAATCTTGTTTCAAGAACAATAATGTATAGAATGAATAAAGAAGAGGTTAAAAACTCAACCATTTGCCTACACATGCATGCATGCTTATATGTGCACTCATTAACATGACAATAGACACACCAACGAACTATCTGAGATGAAATTTGGCTCCTAGATCCTCTGCTTAGGTATTCTGTTCAAAACCTAGCTTTTAATTTGTATTTTTATGAAAATAGCTCATATTTAGTAATGATATTATTATTAACATGTTAAAATAATATTTTTATTATATTATATTTAAAATAATATTATATTAAGACAATATACTACTTTAATAATATTATTATAGTAAAAATAATATCATTTTCCATGTTCACTCTCTTTTAGACACTTGTCCAAATAAAATCAGGACTAGGTGAGTCAAAGCATGTTCATTTTATATATTGATGTTTAATTTATTTGTGCTTTTAATAATATTTTATTTATTCATTAACTTTTTTGTATATGTATACAATATATTTTGATCTTTATCTACTCCCACACACACATCATCTCTCCAACTATCCTAGAGACCCATGAACACATCTTTCTCAATACTTAATGGCTTTTAATTTTTTATTATTATTAGTTAAATGAGTTCAATTAGTGCTATTTGTGACTGGGATAAAACTGTCTAGAAGGGATGAGAACTGTGGTATACCATGGGCCACACCCATATTAAAGGACTCTTAACTCCTCCAGAAGCCATCAAATGTCAATATCTCTTTAGTTAGGGGTGGATGCTCCTGACCCCTCCCAACTCCACTCCATTCTAGAATGTTGACTAGCTTTATCTGGCCTAGGTCTTGTGCAGATGATCATGGCTGCTATGAGCTCATGATTGTAAAAAACATGCCATGTTGAGAAAACATAATTTCACAGCTCTCCTCCCCACACAGATAACCATTGTTGTCTGAGCGTCCATTGTCACTTATCCTCATAACTTTGGCCAGTTATGAATTTCTGCATGAAATTTCACTATAAATGTTGACCTGAGGCCAGATGTGGAGGCACACACTTAATCGCAACACTTAGTAGATGGATCTCTGTGAGTCTGAGGACAGCCTGGTCTATATAGTGAGCTCCAAGACAGCTAGAGTCTCTATTTAAAAAAATAAAAATAAATTTTAAAATTGACTTTACCAAAGTTTAAGGAAGCACAAACCTATGAGTATTTTTTCAAGATATTATCTTGGAAATATGGCAGTTTATCACAACTGTCTCCCCACTAAGAGGTTATCACCTCTTAGGCTCTGCTATGTCCATTGTCTTGAGCTTTTGACAAGAATTACAATAGAAACCCATCCCTATTCCTTTTCTTCCCTAGACATGAGATACCTCCCATTGATTTGGCATCAAATCCAATCAGAAAGTGGTTGATCCCCCCACCAGGATAGCCTTTATTGCACCAGTGGGCATAAGGCTGTCTTTGTAGCATGCAGGGACATTATCATGGTGGCATCATCGATGTCTTACCTCACATACACAGCCTACGGAGTACCTCCAATCACTATGGATGCCAGCCAACAGGAAGAGAGCTTCCTTGTCCATTTGGGATTGATTTTCTGATGCCCTGCAACCACAGCATGTATTGTTTTCAGCAACTGTGTCCTACCATTTAGTTATGATAAACAATCAAGAGATATGGCAATAGTCAAGTTGTTTTGGTTTCTCCAAGGCCTGCCTTCACTAATAAATGGTAAGGTGGTACCCCATGCCTGCAAATGAGATTTTTACTGAAAAACTCATGTCATCTGCGAACAGTATTGTATATTTGTAGGGTTCCTCTGATGAAACTTTTTTTTAATGCTACATTTTTAATTAGTTTACAAAGGAGTAAATTACTGTAATTTTGGTTGACTCAGCCCTATCTTTGTTCTTCCCTGTGCCTCCTCATGCTCACTGTTAGTTTTGGTTGGCCCAACCCTACCTCTATTCTTCCCTTTGCTCTGACCACATCCATACTTGCTCCTTTATCTCACAGCATCCTGCCTCTAATCTTCCCTGTCACCCTTGTCACATTTTCTAGTTTAATTATATTTTATACTTTAGTTTCAATGTCATGGGCTTCCATATAAATTTTTAATAACCCCTTATTTTAGTTAAACTTTCTACCATACCCTGATTTCCCCATTCCACAACCAACTAAATGATTGAACCTTTCCATCCCAGTACTCTTCTTTATACTTTCATTTTAATAATATTCCACTATCTCACCTCCTTTCATGACTCTGCCCCTAATGACCAGTTTCTAATTCCCTGGCTTCCACACATACTCCAAATTAAACATACAAAATAAAAGATTCAAATATAAGCTCCAGATGTGAGATACAACACGTACAGTTTGTCTTTCAGGCCCAGATGACCTCGTTGAGTATAGTTTCCAGTTCCTCCTATTTACTTACAAAGTTCATGATTTCATTTTTCCATGTGGCTGAGTAATATTCCATCTTATATATTAAAATGTTTTTCTTATCATGTATGTATGCTCATCAACTGATGAGCATCTAGATCAATTTCATGTCTTAGTATTGTGAACTTAACTGCAATGAGCATGGACATTCAAGAATTTAATAGCTATCAAAGTCCTTTGGGTACATATCTAGAAATGGTGTAGGTGGGTCACATGGGAATTATGTTTCCAGTTTTAGAATTTTTGTCTATTTGATATGCCTAATTTTTTTTGTTTGGTTGGTTTAAATGCTTTGTATATTGGAGACACTACTCTTCCATCAGATGTGTAGCTGGCAAAGATCTTCCCCATTCCCTAGGATGTCTCTGCATTTAATTTACAGATTCATTCACTGTAGTTTTTAATTTCATATTTGACCATTTCTGATATTACTTCCTTACTACAGGAATCCTATTCAGAATTCACACCTGTGTCTATAGTCAAAGCACACTTCCTACTATCTCCTCTATGGGCTTCAGACTACTAAGTTTTATGTCACAGCTCTTTTCCATTTGGAGTTGGGCTGAGTTGCAGGATGAGAGGAAAGGATCCAGGCCCATTTTTCCAAATCTTAAGATCCAATTTTCCCAGTTTTACTCTTTAGCAATTCTATCTCTTTATTGAATTATACATACTTAGTTTAGATCATTTTTCTTCTTTTGGTGGAGAGACAATATCCAGAAGGTGACTAATTCTTACTAGTGTTGAGATGACATTTTACTCTGTGGGCCTGGTAGTGAGGGATTCAAGTTCTACCCCTACTATTCCTCTGAGAGTCAGTTGCTGTCTGGACCTTGAGTGAGACTCCTGCTGTAGGGCATGTGGGATTTGGTAAATCCCAATGTGAAAGACCCTTGAAACAAAAAAAATATTTTAGTTTAGATTCATAAAAACTACAGACCCATCCAAAATACATAACTATATAAGTTGTAAGAAACCTCATTTTAGGTATTGATATAAAATTATTTATCATATTAAATTTCCCATATATGGAAAACTTCAATTAAAAATTCATGTATATTCCCAAGCATACAAAATCCTTTAAAATGTTTTTGCATGATACATTTTGTCAATGTTTGTCTCTTCAATAGGTTGTAATTAATTTATTTTCCAACATCATGAAATATTATGTATTATTATATTAAATATATTAAATAATTCTGTTCCAACCTTACAGATGATGACATCAGAAATTTTCTGGTATATTCTTCTTGGAACCTGAAACATCAATATCAAGATGAAGCACCCTTTTCTCCCAGATTATATATTCCTATTTACCACAAATTACAATTCCTGTCTCCCTGCTTTCTTTTTCAGCATCTTTTCTCATATTCTAATCCATAGTAATGCATTTTTGAATACTTAAATAAATTTATTTCACTTGCATGAATGTCTGAAGAAAGTAAGCTAAAGTGCAATGCACACAGTTATCTATTTGACTTTTTTTAAAGGAGAGGAGGTTTGGAGAGATTGCTTGAAGGATAAGAGTGTTTGCTGCTCTTCCAGAGGACCTGAGCTCACATTGGGCAGCTCACAACTGCAGCTCCAGAGTACCTGGCACCCACTTCTGGAGGCCTTAAGCACTGCACACAGATTACACATACACACACACAAGAATTTCATGATTCCTATACGTTACAGACTCTGCTATTGTGGTTTTCACTTTTAGATTAATTATATTGTTAACTATATACATATTACCTGCAATATGTATATATTATACATAATTATATACTTAAATTATATTGGCTGCTAAAGGTGAACAGTAAAAGGATTGTGTGCTGTTAGTCTTATGTCAACTTGACACAAGCCATAGTCACTCGGGTAGGGAGATGCTGTGGGAAAAGGATCTGGGACTTTTTTATTAATTGATTAAAGGTTGATATGGGAGGTACCAGCTCACTGTGAAAAACACTGATGAGCAAGCCATGAGGAGAGCCAGGTAGTAAGCAGCACTCCTCCTTGGCCTCTGAATCAGACACTAACTCTAAGATCCTGCCTTGACTTCCCCAGGTGATGAACTACAAGCTGTAAGATGAAATAACTCCTTTCCTCCCCAAGTTGCTTTTGGTCATGGTGTTTATCACCGCAATAGAAACCCTGACTAAGACACATGGTGATATTATAGAGCTTAGATCACAAATCATTTCTTTCTTTCTCTCTTTTCTTTTCTTTCTTCTTTCCTCCTTTAATTTCTTCCTTTCTTCCTTCCTCTCTTTTTTTCTTTTTGGTTTTTCAAGGCAGGACTTCTCTGTTTAACCCTGGCTGTCCTGAAGCTTGCTCTGTAGACCAGGCTGGCCTTAAACTCAGAGATCCATATGTTTATGCTTCCCCAGTTTTGGAAATAAAAGCATGTGTCAGCAACACCCAGTAACAAGTCATTTCTTAAAAGAAAGTTGAAAGTACATTCCCAAATACCCACCTCTATAAAAAATGTAATCTAGTATAGAACACAAGAATCATTTTATTTATTTTTTCCTATTGGGTAGACCATGTTCATATCACTTCTTGTCTGGTATCTTCATTTTTAGGAATTTTTCAAAAGAAAATGTCTTTCTAAAAATTTTTTGCTGAACTTCTTAATTAATGTATTTACTTATCTGAAATCATTTATCAAATATTTAAAATAAATGCATACATGTGCTTGTTCATAAAATATGTTATCAAAATATCAGTGGAAGTCCCTGTTACTATGGATTTGCATAGATAACATGCTGAAGAGTTTATGAAATAAAAATGTGCACACAGGTAACAGTTTTCTTCTTTCTGGGAGAAATGCAAAGAAGTGGAAATGAGTTCTGATGAAAACAGTGTCAAAATGAGCTGTATGTACAGTTATTTTGGAAATAGTCCTGTAGCAGAAAATCTTTGTGGATTAATTAAAATGTTTACAGAAGCCTACTGCCATCTAAACACTTAAAGAAAATTTGGAAAATAGTACTGTATTTACCATATCATTTGAAAATGATACATGACTACACTGTATGAGCATCATTCCCAACCCCTCCTTCATTCCCTCCAACTCCTTTTGTGTCCCTCCACCACTCCTCAAGCTCATGATTTCTTTTTATAATTATTTTTGTTTTAGATAGATGATGTATATACATATATACATACATGCATATGTACATAGGCAGATAGTGGTTATATAGATAGATTCACACATACATTGATAAGATATGATAGATGATTATCAGATAAATAACATAGATTTACATTATAAATAGCTCTACAATAGAAAATGATACATAGACAATAGATAAAACATATGACTATATT
