# Supplementary material for: Comprehensive Analysis of Rodent-Specific Probasin Gene Reveals Its Evolutionary Origin in Pseudoautosomal Region and Provides Novel Insights into Rodent Phylogeny
Source: Biology (Basel). 2025 Feb 27;14(3):239. doi: 10.3390/biology14030239 (PMC11940140; doi:10.3390/biology14030239)
Supplement: Supplementary file 1 [file biology-14-00239-s001.zip › Suppl Data Files/gPBSN/gPBSN_Rhombomys opimus.docx]

>REGO01001185.1:c110000-95000 Rhombomys opimus isolate Great_gerbil_Xinjiang_2013_1 scaffold01185, whole genome shotgun sequence

ACCCAATGTCAATGTCAGCACGTAAGTGCCAACTGAGATGCTCATGCCACCATCTTGAAAGGCAGCTATAAAAAGCAGGGAGCAACTCAACGCTGCCAGTCAGTGAGATCCAGGTACCTTCAGGGCAGACACAGTCACTCACACACTATGAGGGTCATCATCCTCCTGCTCACACTGGATCTGCTCGGCGTCTCCTGTGTGATGATGAATAAGAATCTCAGAAAGAAGGTAGCAGGCCTTCATGGGAGAGGGCTGTGTGCAGTGGGTGTGCTGGGCAGAGAGAGACACAGACAGAGGGACAGACACAGAGAGAGGCTTGGGAGGGGCCTGTGTGCAGATGGGGGGTGTAGGGCAACCAAAGACAGAGAAGCAGAGGCAGAAAGACAAAGAGTGGGAAAAGAATGTTCTGAAAGTTGGTATTCTCTATGTCTGGGAGTGCAGAGTGACAAATGACATAGGGAAGTAATCTAATGGATGGTTTTCTGAGAGAGAGAAACAGAGGGACAGAAACCAACTGGTAGAGAGAAAGGTGAAAATGCTCTGAAAGTTGGTATTCTCTACATCTGGGAGTGAAGAGAGATAGAGAAAGAGAGAGAGGGTGGTCTAAAGGGTAGTTTTCTGAGATAGAGTCACACAGAGAGTCCAAGAGACAAAGAGAAAAATAAACACAGAAAGAATGCTTCTGAAGGTAGGTCTTCTGTTTTTCTTTGTATTTGAGAGGGGGAAGAATGTTCTGGAAGTAAGTATTCTCTATGTCCTCAGAGTGGAGAGTGGCAGAGAGAGAGAATGGTCTATAAGGTGGTTTTTTGAGAGATAGAGACTGAGACTAAGAGAGACACAAAAACAAACACATAGATAGACATGCAAAAAGAGACAGAGAAAGAACATCCTTCAGTCTGGCACCAAGAGTGAGTCCAGCGAGCTGGTTACTAAAGGGCCTGATCTATTTTGATCGACAGATTAAATCACTTATTTGTCCAAATTCACATGTCCTTTCCCATAGAACATCTGATTTGAATCATATATAAACCTGATTGCACAGAAACATAAAGGTATCAAGTAGGCTTTGGTCACCTGAAGAACTTATTTTTGTCTTACTATGCATACACGCCAAACCACTTCATGCCAGATCAGTGTTTCTATTCTTCCTACCTGGACACATTTAAAACATACTTGAATGGAAACTGTCTAGACTTGATCATTGCTAGGGGTATGAGAAACCCTACGCCATTATTTAGAGACAGGTGTGTGACTATCCAGATGCAGTAGGTAGGTAATTATGAAGGAATTCTATGTGGGAACCCCTACAGATTGTTCATCTAATGTTTGGGTAGCTTGATGGAGGTGGCGAGGGGGAAGAAATAGTTGTTATGTGGGGAACGCTACACCATTGTTCAGAGAGGGGTGTGTGAGTAGCCTTATACAGGTGGCTGGGGAGGGGTCTTATTAGGGTATTTGTTCAGAGTACGCATACACATAAAGAAGCTAAGGTGTCTAATGCCTTTTTACAAACTCAGATTGGGAACGACCCCATCCAAGCGGATTTCCAGGTTACTATGCTTGCTTGCCTTGTTTCCAAAGCTGCTTTTTTTTTCTCCCCCCAGATTGAAGGGAATTGGAAAACTGTTTATTTAGCTTCCAGTACTGCAGAGAAGATAAAAGAAGGCTCACCCTTACGAACCTACTTCCGTCGCATTGAGTGTACGAAGAAATGCAATAAAATCTTCCTCTATTTTTATGTCAAGTAAGATATAAAAGAAAACAAAACAATCCGTATGATGGGCTGTGCACAGAGAAATAAGATGTTTTCATTCAATACTCATGTCAGTGAACAGCCACAGCACTAAGTCAGGATTCTTACATCACTTTGCTCTGTATGCTCCAGAAAAATGACATATAATATCTGCACTTTTAATTTAGAACATGTGAATGTATTTAGTGTGTAGCAATACAAAGTAAAATAATAAATATCAAGATATAGACAATGATTATATATAAATAATAGATAGATAATTAGATAGATCAATACATACATTCATAGGTAGGTAAATATAGATGAATAGATAAATGATAACTATACCTTTCTATATGGGGTGAGCATAGAACACATGAAAGAAATGGGACTGGATGTAAAATGCAAGTAGGAAACCCAGTGTTCAAAATCAGATCCTGGTTGAAGTCAAACAGCACAAGACAAGTTACCAAAATAATATACATCACAATCACTTAATTTTCTATTTTTCACTCCTCAAAAACCCAGGTTGGGAAGAAGATACATCAAAAAAATTGGGACAAATTGAGTGCTTTTAAATTATATGGAAATGATAAAATTATCTACTAAGGATATATTTCTATCTAGGATCAAGAAAGTTTTTGTTAATTACATGCTCATATTTGAACCTTTTCATTAATTGATTATTTATAATTTTAGTTTTCTGAGACAGAGTCTCTGTACTCAGTTGTTGTACATTGTGCTGGCTTTGAAGTGAATATAGTTTAAAGAATGAACTTTCCAAATTTTATTTATTATATATACTCATATAATAAATAATTTAATAAATATATCAGAGATCTACCTGCCTCTGCATCCACAGTGCTAGGATTAATGATATGTGCCACCACCATCCAGCTAATTTATATACATATATATATATATATATATATATATATGTATGTATATATATATGTGTGTGTATATATATATGTATGTATGTAATAGGTTCTCATTTTACAGCCCTGTCTGGCTTCAAATTCTCTCTACCTGCCTCTTCCTCTGGAGTGCTGGGGTCAATAATGTATACTACCACACCCAGCCTATATTATATGTTTTTATTTATTTTTATTTTTTAATTATTTTTTTCAGAATTACAGGGAACCTATCCTTCTGCATCAGTCTGCCAACTATTGGGAAGACAAGAGATGCCACCAAGGTTTACTTGGTGTTTAAACTTAGCATGCCTAAAATTATTTTAAAAGCTAATAGCTTAAAATCAAGTAGGTGTATACACTAACATGTTGTTGAAAGAGAAATAAAATTTTTAATTCATAAATACACTTTTGAAAATAAAGCAGAATTACATCATTCCCCTTTTCCTTTCTCCCTTCAACCCTTCCCAAGTACCCTCTTCCTTTGGGTTCTTTCAAATTCTAGGCTCCCTTTTCTTTAATTGTTCATGTACAATATTAAATAAATAGATACATCTTGTTAATTCCATATAAAGTTTATATATATATATATTATATTTATATATATGATTTCACAGCAAACGCTGGGTATTCAGAAGCCAATGCAGAGGGAGAGCTTTTCTTCAGGGAACACTAAAAGATATCTTAAAATTCATATGCCCCCCCAAAAGACCCCAGGAGATCCAAAAAAAAATTCTAAGCAAAAAGAACAGTGATAGAGGGAACACCATCCCAGACACGAGTGATTTTTTTAAAGCGAAAAGAATGGATCCAAAGGCCTTCAATGGGCTTCATAGAGATACATCCCTGGTCTAAACTGTTATTCTCATTTCTTTTTATTATTATTATGTGGTTGTGAATTTAACCTTTAATGACTGAGCCATATCTCCAGCCCAGTTATTTCCATGTTTTAAAACCATAAAGACAAGCCTAAGCTGTGGCTGTCACTACTGATGACATGGACCTCTGGTGAGTGGATACAGATCTGAATACATGACCACGCCTCTGACTCTAAAGATCTCCTGATTTGTCGATGCACACCCCAATTTAAATCTTCTCAGAAAGCATTGTAAGCCATTTAAATGCAGAAAGCAATTTCTGTTATAAAACCTATAAGATTGGTGAGATTTATGAGAGAGGCAAAAAAAAAAACCATGATTACAATATAAATATGGAAGCTGGAGTACAGTGTATACAAGACGCTGCAATATGCTCTGCACCAGGAGAGCTTGTCCTCTTTTAACTTAATGTCTACAGGCATTTCTCATTTCAGATTGGGGTAGAATCTTTGTTCAATACTTCAGGGATTTTTATCAATAGTTGTTATTATGTGCTGGGGATACCCCTGTGCTAAGAGAGTGATTACCCAGCATAAGCCAGAGCCCAGGGCTACATTATCAGCACGTTGGTGTGGTCCTACATTCCCATCATTCCAGCACTTGATGTGTGGAGGCAGGAAGATCAGGGTTTTGCATTCATTCATTAGTCAGGGATCAGAATAGGCAACAGGTATCACACAGAGGAACTGACTCTGCCCTATCAAAGACACCTAAAACCCCCTAACAGGGTGATTGATGGTATTTAAGGGTGCTATGGATTATATGTAAGGACAATGTCCTACAACAACATATTTTTATGAAAGATCTACAGTTGAAAATGTGGTTTGCATTAAACTCTCCTGCCATTCATGTAGAATCATGACCCTGAAGTGATTCACAATAAGACAACATCCATCCATTTGTTTTTCAGGAGAGGATCCAACTGCCAACAGCAAGAAATCATTGGAAGAAAAAGGCAAGAATTTTACCAGGCAGAATGTGAGTAGATGATGCCAGGGTGAATGTTTTTGGTTTTTTGTTTTTGTTTTTGGTTTTGTTTTTTTTAATTCATGGACTTAGTTGAATTTTTCATATCTGAATTTCTATATTTCCCCAATGCAATCATTTCTACAAATTACAGGTGCAATCTCGAAGAAAGCAGGTTCATTTTCTTCCTAGTCATTTGGATATAATAATAGATATGGTTTATTTGTTTGTTTGTTAATGACTTTTAAAAAAACTTTTATTTAGTGTGTGTTTGTACCAAGCACACTTCCCTCCTGTAACTTTAGGCCTAAACTAAATCACAGACAGGAGTCTGAGAATGGTTAAAGTGATCAAAAATGATTTGAGCATTAAAAAAAAAAAAAACGATGCAAAATAGAAACGAAGATTTATCATAGATTAATATGACTCCCAGAGATTTTCAAACATGAATCTTGTATATGGAATAATGACTGATTTACATTTTATAATTTTATTGGGGAAAATCATTTACAATATGTAAATATAATCTTCATGAATACTCCTATTTTTCAGTTTGGACAGGAATATGACCTAAAATACACCAAGATCCATTTAGAAATAATAATGGTATCAGTTTCATTTGTCGTGAAACAGGTGGTAAGGAGACTTTCACTATTTTCTATTCCACCATAAACAGTCTTTTTAACCCAACACAATAATACTGATAGGCCAAACACATCACCAACTCTGCCTTGTATTTTCAGTGAGTTTATTGGATTTAAATACAGGAGCAGACTCAACTCACAAACAGATATCCAGGCAAAGAGTCCCATTCTCTCTTGTATGTTATGTAATTTAGTGTGAGGCCAGCCCGTTCTGCACAAGTTTCAGGCTACATAGTGAGACCTGTCTCAGAAGGAAAATGACTTTCCACTGACATGAGAAGGAACATCATCACGTTGTTACAGAGGGTCTTGTGCCTACCCCTTCCCTTTTACAAGAAGAAATGTTAGTTAACCAGTAAATGTATTGAAGTCATTTACAAGAGCATCTGTGACTCAAATATGGTTGCATTCCTGAAGAGCCTAATTCAGCTTGGTGACACTCATGAAAGCTGTATCCCTGGAGCTTTGTGTAAGGCTTACAGGCAGCTGAGCTCTTCAGAAAGCCTTTTTTTTTTTGGAGATTGTTCACTGTTTCCCATAACTTTGTGGAGTGGCCTACTGAAAGTCGTTATTCCCAAAAAAGTCCTGAGACTTGTGATTTATTTATTTTATGAGCATCCAAGAAAGTCCCAGTTTGGAGAAAATTGCTATGTGGCACCTGATCTTGTTTATCTGTCATGTGTCTGAGGAAGAATTAGTAAAGCTAGATAATTACATCACTACATGAAAATCAGATCCATGAAAATACCTCCATATATGTATGTATGTATGTATATATATGTGTATATATATGTATATAGGTAGGTATATGCATATATATTATCATACAGCATTCCTCGATGTATATATACATATATATACATTGTATATATATGAATGTATATACGTATATGTATATATCTGTATATGTACATATATATGTCTAGACAATTCCACCATTAAATGTAATAGGATCCATGAAAGTGCCAGTATATGTACATATATATAAACCATACAGCTTTCCTCAATATGTATATATGTATAACCAATTCCACCATTAAATGTAATAGGATCCATGAAAATGTATATACATATATATACATGTTTGTGTATACATAAAGTCATACAGAGCTGTCTTCAGCAAGTGAAATATTGCAGTGTGCCACCTACTATGCACTTATACACATCCAAGTATCAGGAACACCACTATCCCAAATTAATATAGTTTCTATATTTCTTTTTTTTTAGTTTCTATATTTCTAAGCAACTATGGGATACTAGATATATTTCATATCTTAAAGCTGGATTATACACTTGCTTTCTTAATCCCCTGTCTGTTTTGACAGGTTTTGACACATGGAGAAGAATATTGTGGGGCAGACTATGTACCCATTCATAACGGTGCAGTTAGGATGAGCCTCGACAATCATTTTGTTGACTTCATATGTATTTCTTATCTATCCCAGATGACGGAAAAGTAACATTCATGGTAAAGATGGTGAATGATAAGATATTGCTGTTTTATTATTTTAACAAGGACAAGAGAAATAAAGTCACGCGAGTGGCTGGAGTTTTGGGTGAGTGCCACACATGGAACTTATCATCTGAGTGTATAGTTCAAGGAGAGGGGTTCATGCTCATCTCTGTGCAGTGTGTTTGTGTGTCTGATTTTGTTCTTTATGTTGATAAACCACTGATGCTGTGAAGGTTAGCTTTGAGTACCAATTTGTCACAACACACAATCCCCTGGGAAGGGTCTCAGTAGGGAATTTCCCACAGAAGACTGGCCTGTGGGCTTGTATTTATTGATGATTGACATAAGAAGGCTCTGCCCACTGTGGTTGGAATCATTGCCTAGCAAGAATATGTGCTGTGAGCTTCAACTTTCAGCAGACAAAGCTCAGCATCCTCTGGGAAGGGCCTTATGGAGGCTATTTCTCTCATCAGACCAGCCTGTGGGCATGTCTGGGGAAGGATATTGAATGATGATTGATATAGGATGCTCTGCACACTGACATGAGCAGCATTCCCTGGACAGTTCATCCTAAACTGTATCAGACTGGAGAAAGCTGGATAAATGCAAGCACACATGCATGCATTCTTTGCTGTCTGTTCCTCACTATAGACATGGTGTGTCAGATCCTCCAAATTTCTCCTACTGGAATTCCCCCACTTCAAGGTGCTATGACTGGAAAAGTACTGCATAAATAAACCCTTTCCTTCCATAAATTGCTTTTGTCAAGCCATTTGATCAGAATATTGAGCAAGGAAATTGGGATGTTGAGAGTTTATTTGTTGCAAATTAATAAATATTTGTTTCTGTTGGGACCGAGAAGGTTTTAATAAAATTCCCACACATCTTTTTCAGCATTTCTGAATTATATATCAACCATACATCATGTATCTCATGCCACTGTAATCCTGGATTTCCACCTCTAACAAAGCAAGAATTGATATATTACTTGACCACATTAAATGTGTTTGTTGATTGAATTTATGTCAACTGCAAACACTGGATTTCAGCTGTTGTTTCTGGCAATCTTTAGAACTTCTGACTTTTAAGTCAGAATTATCACATAACATGAGTTCTGACTGTACTGAGACATGTCCACAAAATTCAGTTTTTCATACATCCAAATTCTTTGGAACTGACAAATTCTACAACTCTGTCATCAAAGGCAGAATTAATTTTTGTTGAAATTAATAGGAAGAGATTGTATTTCATGGGGACTTGTTCCTCAGTAGCCAAGTTTGGGGTTTTGTTTTATGTGTACTAGTTATTTACCTGCATGTATGTGCACTTTGTGTGTACCTGGTGCATACAATGACCACAAGATGGCATCGGATCCCCTGAAACTGGACTTACAAGTGGCTGAGCACCACCATGAGGGATCTGGGAGCTGAATGCCGGTCCTTAAGCATTCTTAACCAGTAAGCCAAAAGTCATTCCCCTCATCTTTATGATGAGTTTAGACCAATTGGTTCTGCCATCTGAGGAAGCATCCAGAACCCTTAGGAATGGATTCAAGAATATAAACAGAAGCAACATAATGACATACAGTTATAATAGTAAAAACACTAAGTATCAATCATAATTTTCTGGAATTGATTTGGAGGCTGTGTATGTGTTCTGTATGCTCAAATCTACACAAAAAGTCAATGTTACAAAAATAGGAGTCATGGTATATTATACATTGTGATCCACCCACTACTTTCATTTCTCATGAGAAAGATGCAGTTCCATGCTGCCAATATATTGAAGGAAATGGGAGTGATCTTGTGTTTTGGCAGTTTTTACTAGAATAAGCATTGGCAAATATTTTATTAATGTCCTGGATGCTAATGTTGTGACATCTTCTTCCTTATACACCATGAAACACAGCAAAAGCCAATCAACTGACTAAGGATGAGATGACGCAGTACATGGACTTGGTGGAAGAAATTGGCATTGAGGATGAAAATGTAATACGTGTCCTGGACACAGGTAAAATAGAAACCTGTGTGGGTAACTTCTTACATTGTTTTTTTAGTTTTTTAACTTATTTTTTATTTATTACAAAATATTCACTTTGTATCCCAGCTGTAGCCCTCTCCCTCTTTCCCTCTCAATTCTGCCCTCCCTCCCTCATCTCTTTCCATGCCCCTCTCTAAGTTCACTGATTGGGGAGGTCCTTCTCCACTGCCATCTGACCCTAGCCTATCAGGTCTCATCAGGACTGCCTGCATTGTCCTCCTCTGTGGACTGGTAAGGTCGCCCCCCCTTCAAGGGCAAGTGATCAAAGAGCCAGCCAATGAGTTCATGTCAGAGACAGACCCTGCTCCCCTTACTAGGGAGCTCATTTGGAGAATGAGTTGCCATGGGCTACATCTGTGCAGAGGATCTGGGATATCTCCATGCATGGTCCTTGATTGGAATATCAGTCTCATAAGACACTCCTGTGACCAGATTTTTTGGTTCTGCTGCTCTTCTTGTGGAGCTCCTGTCCCCTCCAGGTCTTTCTGTCTTCCCCTTCATTCATAAGATTCCCTGCACTCTGTCCAAAGTTTGGCTATGACTCTCAGCATCTGCTTTGATACCCTGCTGGGTACAGTCTTTCAGAGGCCCTCTGTGATAAGCTCCTATCTTATTCTCTGTTTTTTCCCTCTTCCAATGTCTATCCTGTTTGCCTTTCTGAATGAGGATTGAGCATCTTACCCAGGGTCATCCTTCTTGCTTAGCTTCTTTAAGTGTACAGATTTTAATATGTTTATCCTATATTATAAGTCTAATATCCACTTATAGGTAAGTATATACCATGTGTGTCTTTCTGATTCTGGGTTACCTCACTCAGGATGATCTTTTCTAGATCCTACCATTCACCTGAAAATTTCATGATTTCCTTTTTTTTTAATTGCTGAGTAATATTCATTGTGTAAATGTACCACAATTTCTGTATCCATTCCTCAGTTGAGGAACATCTGGATTGTTTAAAGAGTCTGACTATTATGAATAAAGCTGCCCTGAACATCTTACCTTGCATTTTTATAACAAATGTTTTTTTAAGTATCTAAGATTTTGGGAACATGCTTCAGTGGGCAAAGTGTACACAAGCATAAGGACTCAAGTTTGGTCCTAGTATCCATTTAGAAAGCTAAGGAACACCAGAGCTGAGAAGGGGTGGGGAACAGGAGGAACCCTGGAGATTTCTGGCCACAGATCTATCTGAAAGATCTTGTCTCAAAAATAATAATGTGTATAATAAATTAGGAAGATGTTAAAAATCCAACCACTAACCTATACATAGATGGGTGCATATATGTACCTCAGGTGAGTAAGGAATGAACCATCTAGACAGATCCACACATATAAAATCCACCTAAAATGAAACCCAACCCTAAGTTCCTCTTTTCAGATATCCTGTTCAAAACCTATATTTTATCTGCATTTTTATGAAAACTGTTCATATGTAATAATATTAGTATAATGTAGTACTTTAACATAATATTATTATATTAAAATCATATATTACTTTAAAATAATGTTGATATATTAAAATAATATTATTTTCCATGTTTCTTCTCTTTTAGACACCTGTCCAAGGAAAATCAGAGATAGGTAAGTCAAACTACATTAATTTATATCTTGATGTTTAATTTAAATTAATTTGTTTTTTAAATAATATTTTATCTGTTTTTAAACCATCTTATATATGAATATAATATATCCTGATCTTACGCTCTGCTCTACACACATCATACTCTCAACTATCCCAAAGATCCACCAACACAACTCCCTCTATACCTCATGGCTTTGAAAATTCTTATTATTATTAACTCAGTGGGTCCAATTGGTGCTATTTGTGAATGACATTGTGGCCGTTCCTGGAGGTATGGGCAATGTAATGTACCAGGGTCCACACCCTTACAAAAAATGAACACTGGAAGGTAGTGCTGCTCCCCTTTAATCCTGAACTGGAGAGGTAAAAGCAAGCAGATCTCTGAGTTTGAATCCAGCCTAGTCTACAGAGGGAGTTCCAGGACAGCTGTGGCTACACAGAGAAATCCAGAAGAAGAGNNNNNNNNNNNNNNNNNNNNNNNNNNNNNNNNNNNNNNNNNNNNNNNNNNNNNNNNNNNNNNNNNNNNNNNNNNNNNNNNNNNNNNNNNNNNNNNNNNNNNNNNNNNNNNNNNNNNNNNNNNNNNNNNNNNNNNNNNNNNNNNNNNNNNNNNNNNNNNNNNNNNNNNNNNNNNNNNNNNNNNNNNNNNNNNNNNNNNNNNNNNNNNNNNNNNNNNNNNNNNNNNNNNNNNNNNNNNNNNNNNNNNNNNNNNNNNNNNNNNNNNNNNNNNNNNNNNNNNNNNNNNNNNNNNNNNNNNNNNNNNNNNNNNNNNNNNNNNNNNNNNNNNNNNNNNNNNNNNNNNNNNNNNNNNNNNNNNNNNNNNNNNNNNNNNNNNNNNNNNNNNNNNNNNNNNNNNNNNNNAGAGAAGAGAAGAGAAGAGAAGAGAAGAGAAGAGAAGAGAAGAGAAGAGAAGAGAAGAGAAGAGAAGAGAAGAGAAGAGAAGAAGAGAAAAGAAAGAGAGAAAAACTCTCCCCCAGACTCCATCAACTATCAATGGCTCTTTGTTTAAGAGTGGGAGCTCCTGAGCCCCTCTCTGCTTCATTCTAGAATGTTGACTGAGTTAATCTGGCCAGGGTCTTGTGTAGGTGACCACAGCTGCTGTGAGTCCATGGTTGTAACTGCTGTGTCATGTTCAGAAAACAGAATTTCATGACTCTCCTCCCGGCCCAGATAACCATCAATGGTTGAGCATCCATCCACAGTCATTTATGCACAGCACTTTGACCAGCAATGAATTTCTGCATGAACCACTTCCTACTATACGAAGTTTACCTGAGGCTGGATATGGAGGCACACATGATTAACCCCAACACTTGGGGAGCAGAAGTAGATGAATCTCCGTGAGTTTGAGGCCAGCCTGGCCTATATAGTTAGCTCCAGGACAGACAGGGTACAGTCTCTATCTAAAAACAAAAAAGGCAAAAAGTTGAGTTGATTTGACCACAGTTGAGAGAAGAATAAATCTATGAGTATTTTTTTGAGACACTAGCTTGGAAACATGACAGTTTATCACCACTTGTCTCCTCCATAGGCTAGAATTGTAATAGGAATCCATTCCTACCTGTGTTCTTTCCTAAATATGAGATATCTTCCATGACATCAGATTTAATCAGAAAGTGGTTGTTTCCTCCAGGACAGACTTTATTGCACCAGTGGGCAAAAGCTGCTTTTGTAGTGTGCAGGGGTGGGGGTGTGGTAACATCACTGATGTTTTATTTCACATACACAGCCTATGCAGCACTTCTAAGCACTGTAGAAGAGTTTTCTAGTACATTTGAAATTGATTTATTGATGCCTTGCAACCACATCATGAGGTGTGTCCAGCAGTGTTGTTCTACCATTTAGCTATCAAAAGATATGGCGATAGCCGGGTTGTCTTGGTGATTCCAGGGTCTCCCCTCACTAACAAATGATAGGGTGTTACCCCATGCCTGGAAATTAGATTTTCACTGAATAACCCGTGTTTTCTGGGAACAGCATTTTACCATTGCAGGGATCCTCTAATGAAACTTTTTAAAATACTATATTTTTAATTAATTTACAAACTAGTGGATTTCTGTAAGACTTCTTCATACACCTTTAGTTTTTGTGACCCACCTCTACCATTGTTCTTCTCTATGCCCCAACCACATCCACACTTGCTCCTTCAATCCACAGCACTCCCCTCTAATCTTCTCTGTCATTTGTGTTCAATTATATCACCTGTCTAATTATATTTTATATCTTAATCACACGGTCAGGGGTTGAATTTTTAATAACACTCCATTTTGGTTAAACCTTCCACCATGCTATGATTTCCCCATTACACATCCAATTCCATTATTGGATTCTTCTTATTTTAATGGCATTTCACTATCTCCCCTCCCTTGATGGACCCACAGTAAATGACCTGTTTCTAATTACGTGGCTTCCACATATAGTCTAAATTAAACATACAAAATAAAAGATTTAAAACTAATATCCACATTTGAGATAGAATGTGCAGTTTGTTTTTCTGAGCCTGGGTGACCTCATTGAGTATATTTTCCAATTCCTTCTATTTACTTGCAAATTTCATATTTCATTTTTCTCAATGTGATTAATATTCCATTTTATATATCATATCTTCCTTATCCATTCATTCATTATTTGATGAACATTTAACTCAATTTTGTTTCCTTGCTATTGTGAACTTAACTGCAATGAGAATAGACACGCAAGGGTCTCTGTAATAAGATATAAAGCCCTTTGGGTACATATCTAAAATGGATATGTAGCTGAGTCACATGGGAACTCTGTTCCTAATTTTGCTTTTGTTTGTTGTTCATAATTTTTGTTTCTGTCTTATTTTGTGGATTTTTTAAGTTCCTTGTATATTGCAGACACTAATACTCCATCAGATGTGTAGCTGGCAAGGCTCTTCACCATTACCTGAGATGCCTCTGCATTTACTTGACAGCCTCCTTTGCTTATAGTTTTTAATTTCATTATATCAGAGCAGTGTTAGTTGTACTTCCTTGCTACAAGAATTCTATTCAGAGAGCCCACACCTGTGCCTATGGTCAACACACACTCCCTACATTCTCCCCTACCAGCTTTCAGAATAGCAGGTCTTTTTTAAAGGTCCTTGATCCACTTGGAGTTGAGGTTTTTTGTTTGTTTGTTTGTTTGTTTTTCACTGTGACAAGGAAGGGCCCAGATTCATTTTTCTGCATTTTGAGGCTCACTTTTCCCAGTTTGTTTATTCTTCAAAAATTGTATGTTTATTGAATTATATATACATATATTTAGGTTAGGTCATTTTCTTTCTTTCAGTGGAGACATTGACATCATTCAGAAAAGGACTAATTCTTGCTAGTGTTGAGATGGCATTCTCCTCTGTGGAGCGGTATTGAGAGCTGCAAGCTCTTCCCCTAGTCTTCCTCTGAGAGTCAGCTACTCTATGGGATCTTGAGTGAGACTCCTGCTGTAGGGTACATGGGGCTTTGTGAACCTCAATATGAAAGTTGAAACAAACAAAAAACAAATGTATATGATTTTAATTTAGATTCATAGAAACTACAAACTCAAGTAAGCACATAAATGCATACCTTCTTTTTAAAAATCCAAATTTAGGTCATAATATGAGATCGTTTATCATATTAAACTTCTCCACATATAGAAAACTTTATGTGTATTTCTAAACATACAAAATCCTGTGAAATGGTTATACATGATACATCTTGTCATGGTTTGCCTCTTTCACGTGTTGTATTTCTTTTATTTTTACTCTCATCAAATATAATGTATTACTATCTTAAATATATTTAATAATTCTGTTCCAACCTTACAGATGACAACATGTGGAATTTTCCAGTACATTCTTCCTAAATTTCTGGAACATCAATATGAAGATGAAGCAACCTTTTCTCTCAGATTGCATCTTCCTATATGCTGCAAACTACAATTACCATCTCCCTACTTTCTCTTCCATTCACCCTTTCCTGTTTTTAAATCAGTTTTAGTGTATATTTGGAAGTTAAATAAATTTATTTCACTTGCATACGTGTCTCTGAAGAAAGGAAGCTAAACTGAAATGCACAGAAATATCAATCTGACTTTTTTAAAAGGAGAAGAGGGTTGGAGGGATGGTTCAAAGGCTAAGCGCACTAGCTGCTCTTCCAGAGGACCTGAGTTCAATTCTTAGCACCCACATTAAGCAGCTCACAATTGCAGCTCCAGGAGACCTGAAACCCTCTTCTGAACTCTTTGAGCACTGCATGCAGATCACACACACACACACACACACACACACACACACACACACAATTTTTAAAAATTCTATTTGAATGCAAGTATGGGTTAATAATATCAGATGATTTTTATTAAGAATAATTTCAGGGGCATGGGTAAGGCTCAGTGGTTAAAGTATCTACCATTTAAACATAAAGTTCAATTATGTGTTCAATTATTAAAATCCAGGTGTGATGGATCATGCCTGTAATTCCAGCACCAGGGAGGGAGGTATAAATAGATGACTTTTCTGATGTCACCAAGAGCCAGTCTAGACTACCTGAAAAATCCCAGGCCAGTGAGACACCATCTCAAAAATAAGGCAGGGGATTAAGAGCACTGGCTGGTCCTACAGAGGACCACACACATGGCAGCTTACTACTGTCTAGATCTGACATGTATGTAGATATACATGCAGGCAAAATGCATAAAATAAAAATAATAAAAGTAATTTTTTAAAGGTAGATGTGCAAGCAGGATAACACCTGACATTGTTCTGCGGCCTTCAACATTCATATGCACACACATACATCCACAAACACAG
