# Supplementary material for: Comprehensive Analysis of Rodent-Specific Probasin Gene Reveals Its Evolutionary Origin in Pseudoautosomal Region and Provides Novel Insights into Rodent Phylogeny
Source: Biology (Basel). 2025 Feb 27;14(3):239. doi: 10.3390/biology14030239 (PMC11940140; doi:10.3390/biology14030239)
Supplement: Supplementary file 1 [file biology-14-00239-s001.zip › Suppl Data Files/pPBSN/pPBSN_Mus minutoides.docx]

>pPBSN_Mus_minutoides

MMRVIILLLTLHVLGVSSVMRNKSLKKKIEGPWRTIYLAASTMEKINEGS

PLRTYFRLILCGRRCNQVYLYFFIKKGTKCQLYKVVGRKKREVYYAQYEG

STAFMLKMVNEKILLFHYFNKNRRNDVTRVAGVLAKGKQLNKEEMTEFMN

LVEEMGIEEENVQRIMDTDNCPSKIKS
